# A multiverse of trophic networks and coevolutionary trajectories among holoparasitic Orobanchaceae and their animal associates: a global perspective

**DOI:** 10.3897/phytokeys.275.192014

**Published:** 2026-06-02

**Authors:** Renata Piwowarczyk, Karolina Wiśniewska, Tomasz Rewicz, Tomasz Olbrycht, Sebastian Salata, Alexander V. Fateryga, Łukasz Nicewicz, Łukasz Depa, Katarzyna Zając, Rudolf Masarovič, Waldemar Celary, Łukasz Mielczarek, Attila Mátis

**Affiliations:** 1 Center for Research and Conservation of Biodiversity, Department of Environmental Biology, Institute of Biology, Jan Kochanowski University, Uniwersytecka 7, 25-406 Kielce, Poland Department of Environmental Biology, Institute of Biology, Jan Kochanowski University Kielce Poland; 2 Department of Invertebrate Zoology & Hydrobiology, Faculty of Biology & Environmental Protection, University of Lodz, Banacha 12/16, 90-237 Łódź, Poland Faculty of Biological Sciences, University of Wrocław Wrocław Poland; 3 Department of Agroecology and Forest Utilization, Faculty of Technology and Life Sciences, University of Rzeszów, Ćwiklińskiej 1a, 35-601 Rzeszów, Poland Faculty of Natural Sciences, University of Silesia in Katowice Katowice Poland; 4 Myrmecological Laboratory, Department of Biodiversity and Evolutionary Taxonomy, Faculty of Biological Sciences, University of Wrocław, Przybyszewskiego 65, 51-148 Wrocław, Poland Faculty of Biology and Geology, Babes-Bolyai University Cluj-Napoca Cluj-Napoca Romania; 5 T.I. Vyazemsky Karadag Scientific Station, Nature Reserve of the Russian Academy of Sciences, Branch of A.O. Kovalevsky Institute of Biology of the Southern Seas, Nauki Str. 24, Kurortnoye, Feodosiya 298188, Crimea Institute of Nature Conservation, Polish Academy of Sciences Kraków Poland; 6 Institute of Biology, Biotechnology and Environmental Protection, Faculty of Natural Sciences, University of Silesia in Katowice, Bankowa 9, 40-007 Katowice, Poland Faculty of Technology and Life Sciences, University of Rzeszów Rzeszów Poland; 7 Institute of Nature Conservation, Polish Academy of Sciences, Al. Adama Mickiewicza 33, 31-120 Kraków, Poland Faculty of Natural Sciences, Comenius University in Bratislava Bratislava Slovakia; 8 Department of Environmental Ecology and Landscape Management, Faculty of Natural Sciences, Comenius University in Bratislava, Mlynská dolina, Ilkovičova 6, 842 15 Bratislava 4, Slovakia Faculty of Biology & Environmental Protection, University of Lodz Łódź Poland; 9 Krakow Municipal Greenery Authority, Forest and Nature Team, Reymonta 20, 30-059 Krakow, Poland T.I. Vyazemsky Karadag Scientific Station, Nature Reserve of the Russian Academy of Sciences, Branch of A.O. Kovalevsky Institute of Biology of the Southern Seas Feodosiya Crimea; 10 Faculty of Biology and Geology, Babes-Bolyai University Cluj-Napoca, Cluj-Napoca, Romania Krakow Municipal Greenery Authority, Forest and Nature Team Krakow Poland

**Keywords:** Co-evolution, DNA barcoding, multitrophic interaction, parasitic plants, phytophages, plant-animal interaction, pollination, species diversity

## Abstract

Holoparasitism, in achlorophyllous, fully heterotrophic plants, is one of the most peculiar symbioses in the plant world. In particular, holoparasites from Orobanchaceae, the largest parasitic plant family, have evolved unique visual and olfactory signals in the plant kingdom, and thus play a key role in the evolution of animal–plant adaptations. Holoparasitism offers excellent case studies of the effects of a specialised interaction on multiple aspects of plant ecology and evolution, including pollination, herbivory, and speciation. In this paper, we present the first global study of these interactions using morphological and molecular tools, summarising almost 20 years of field studies. These observations were supplemented with literature data and internet sources, ultimately encompassing more than 1370 observations from 76 countries in Europe, America, Africa, Asia, and Australia. We found data on animals interacting with 130 species of 16 holoparasitic genera from the Orobanchaceae family. This study represents the first comprehensive study of animals which use these plants as food, shelter, hunting grounds, or part of their development cycles. Our work has resulted in recognising 667 animal species from 34 orders, 163 families, and 434 genera, with a predominance of arthropods (91% of species recorded) followed mainly by gastropods (ca. 4%), mammals (2%), birds and reptiles (0.6% each). Besides the combination of different pollinator and herbivore species, parasitic plants also attract a range of other animals, such as carnivores and parasitoids, creating a habitat with multitrophic and multilayered relationships. Our research sheds light on the intricate interactions mediated by parasitic plants and animals, opening the path for further elucidating the ecological and evolutionary drivers of holoparasite diversity and their broader ecological role.

## Introduction

Plants are an important resource for many animals, which use them for food, shelter or protection (Hesselberg et al. 2023). Coevolution between plants and animals, especially insects, is a major evolutionary force. The relationship has lasted for at least 400 million years, and mainly occurs between plants and herbivores, pollinators (originating 250 million years ago) or seed-dispersing insects, based on various chemical and physical mechanisms (Labandeira 2013; Bruce 2015; Moscrop and Glover 2021). Plant-arthropod interactions are typically complex and can strongly affect plant fitness (directly or indirectly) individually, synergistically, or antagonistically (De-la-Cruz et al. 2022). Insect-plant interaction is not just a bipartite relationship but is mediated by associated competitors, predators, parasitoids, as well as microbiota that interact in either an obligate or facultative manner, temporally or spatially (Sharma G et al. 2021). Much of the research focuses on individual interactions between one insect species and a single plant species; however, in nature, plants are exposed to multiple simultaneous attackers and beneficial organisms, as well as interactions between them (Bascompte and Jordano 2013; Bruce 2015). Still poorly understood, tri-trophic interactions (plant, herbivore, and predator or parasitoid) are very important in maintaining ecological balance and biodiversity (Serdo and Degaga 2023). Moreover, in holoparasitic plants, there can even be a four-trophic interaction with an obligatory host plant. Comparative and long-term data sets of plant-insect arrays remain scarce. Most studies pertain exclusively to the local or regional scale, and research on botanical families in a wider geographical scope and considering the phylogeny of host plants or herbivores is scanty, which limits the assessment of general patterns in herbivore richness (review by Lewinsohn et al. 2005). Beyond the mutualistic interactions among conspecific individuals, most of these interactions are allospecific, involving species, or sets of species, often completely unrelated (Bascompte and Jordano 2013). As much as 18% of terrestrial plant biomass is consumed by herbivores, making herbivory a significant biotic interaction (Cyr and Pace 1993).

Lack of photosynthesis in the plant kingdom is a unique phenomenon, thus, the examination of the evolution of achlorophyllous, fully heterotrophic plants remains one of the most interesting and challenging topics in plant biology and ecology (Suetsugu 2018). Parasitic plants occur in almost all terrestrial vegetation types worldwide (Heide-Jørgensen 2008), and constitute 1.6% of all angiosperms, with ca. 4750 species in 26 families (Nickrent 2020). Parasitism, especially obligate parasitism (holoparasitism, without photosynthesis), is one of the most peculiar symbioses in the plant world, providing the opportunity to study relatively unknown trophic dependencies between partners (Yoder 2001), and their interactions with various abiotic and biotic stresses. Holoparasitic plants absorb water and nutrients from the host roots or shoots, using a specialised organ called a haustorium (Joel et al. 2013). Parasitic plants, although they are quite a large and diverse group, are often regarded as pests, restricted to a quantification of direct effects on hosts, mainly because several species cause damage to agriculture and forestry (Watson 2009; Těšitel et al. 2021). However, they can play key roles in determining community structure and function, and should be considered as both keystone species and allogenic or autogenic ecosystem engineers. In addition, they can considerably impact multiple trophic levels within communities, the diversity and distributions of co-occurring host and nonhost plants, as well as invertebrates, birds, and mammals (Press and Phoenix 2005). In recent years, the number of studies on the interactions of parasitic plants, particularly hemiparasitic ones, with animals has increased. These studies have mostly concerned those that participate in pollination or dispersal, consume their tissues, and use their structures for shelter, as well as parasites that modify herbivory levels of their hosts (e.g., Watson 2001; Belchior et al. 2022). Multilayered trophic interactions involving herbivores, predators, parasitoids, and their symbionts have already been documented for hemiparasitic members of the family, such as *Rhinanthus*, whose impact on plant and animal communities across multiple trophic levels has been extensively described (Chaudron et al. 2021). However, analogous data for holoparasitic plants remain scarce and are only beginning to be documented.

Plant visual and olfactory signals (especially secondary metabolites) play a key role in the evolution of insect-plant associations by selecting insect adaptations (Beran and Petschenka 2022). Having tissues enriched with water and minerals compared to their hosts, parasitic plants attract sufficient numbers of animal visitors, altering or modifying the competitiveness of other plants, matter cycling, and increasing local biodiversity (Watson 2009). However, many fundamental aspects of the ecology of parasitic plants remain poorly studied, and research has been dominated by laboratory or crop pest studies, rather than by observations of natural communities (Pennings and Callaway 2002). There is also a long tradition of humans using parasitic plants for medicinal and cultural purposes. A few parasitic plants, including holoparasitic ones, are even cultivated for their food, industrial or medicinal products (Pignone and Hammer 2016; Těšitel et al. 2021). Thus, studying their herbivores and pollinators may also help in their cultivation. In general, much of the current research focuses on bees, or other insects grouped together as ‘non-bee pollinators’, obscuring the relative contribution of this diverse group of organisms (Doyle et al. 2020). Moreover, most holoparasitic species are rare and endangered plants, because unlike autotrophic plants, they are completely dependent on the host, therefore they may be more affected by environmental changes, such as climate change (Piwowarczyk and Kolanowska 2023a, 2023b), but they also may be dependent on specific pollinators or exposed to feeding insects, aspects which remain largely unexplored. Therefore, there is a need to specifically increase environmental and ecological research on these plants (Mkala et al. 2022).

**Figure 1.**
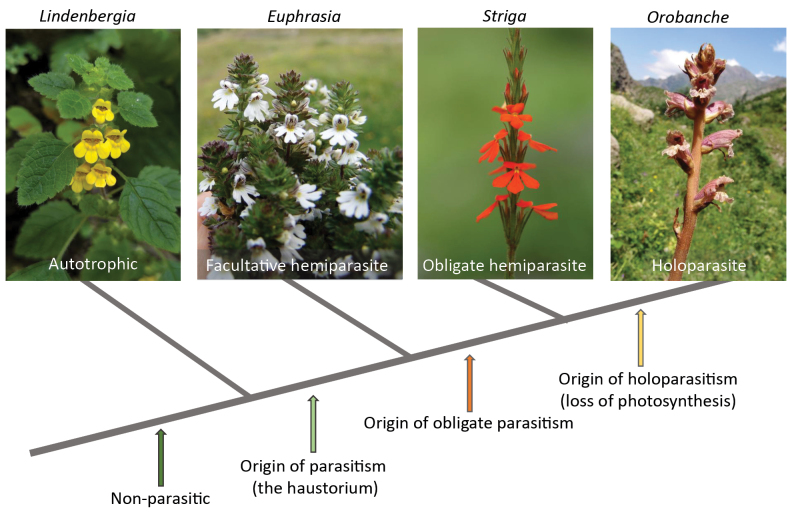
Evolution of Orobanchaceae – most important events from non-parasitic to various levels of parasitism. *Lindenbergia muraria* (India) – photo by Sagnik Dutta Roy from the iNaturalist website (https://www.inaturalist.org/photos/579697675) distributed under the terms of the Creative Commons CC BY 4.0 license (https://creativecommons.org/licenses/by/4.0/); *Striga elegans* (South Africa) – photo by Sharon Louw from the iNaturalist website (https://www.inaturalist.org/photos/248759405) distributed under the terms of the Creative Commons CC BY-NC 4.0 license (https://creativecommons.org/licenses/by-nc/4.0/); *Euphrasia* sp., *Orobanche raddeana* (Georgia) – photos by R. Piwowarczyk.

Orobanchaceae is the largest parasitic plant family, with 102 genera and over 2,100 species (Nickrent 2020). This family comprises non-parasitic to both root hemi- and holoparasitic species (Fig. 1), differentiated in terms of trophic relationships (Westwood et al. 2010), thus constituting a convenient research model, especially with regard to the circulation and interaction of components between partners and the ecosystem.

The family has a worldwide distribution, but for the 19 genera of holoparasites (the aim of our research), the main centres of distribution are the warmest parts of the Mediterranean, western and central Asia, northern Africa, and North America (Piwowarczyk et al. 2023). Specific genera are also associated with subtropical and tropical regions, as well as the far north in cold zones.

So far, little work has been published on animals, mainly arthropods, associated with the holoparasitic Orobanchaceae. Only a few phytophagous insects have been reported to feed on Orobanchaceae; most works list the monophagous fly *Phytomyza orobanchia* (e.g., Linke et al. 1990; Klein and Kroschel 2002; Černý et al. 2022; Dawah and Deeming 2025), a few *Eumerus* species (e.g., Stackelberg 1961; Waitzbauer 1976; Shaumar and Kamal 1978; Al-Khezraji et al. 1987; Piwowarczyk and Mielczarek 2018a; Aracil et al. 2023), beetles (e.g., Zermane et al. 2001; Ray and Dasgupta 2010), aphids (e.g., Borkowski and Dyki 2008; Holman 2009; Boukhris-Bouhachem et al. 2011; Piwowarczyk et al. 2018b), thrips (e.g., Minaei et al. 2007; Qasem 2011), and moths (Lee 1931; Lee and Goseco 1932; Takano 1934; Ray and Dasgupta 2010). Some arthropods were reported as natural biocontrol agents of invasive holoparasites (e.g., Manjunath and Nagarkatti 1977; Klein and Kroschel 2002). So far, bees (including bumblebees) are recognised as the major pollinators with occasional records of birds and mammals. However, only a few papers include random observations of pollinators (e.g., Jones 1991; Ellis et al. 1999; Ollerton et al. 2007; Wester 2011; Abbate and Campbell 2013; Tóth et al. 2016, 2026; Turner and Midgley 2016; Piwowarczyk and Kasińska 2017; Sharma A et al. 2021; León-Osper and Narbona 2022; Niedzwetzki-Taubert 2022; Nishimura and Takayama 2022). Moreover, little information on pollination ecology is available for certain species or genera of holoparasitic Orobanchaceae, and it is necessary to gather data, such as pollinator observations, to elucidate the reproductive ecology and evolutionary history of this group (Joel et al. 2013).

Molecular methods, particularly DNA barcoding using a fragment of the mitochondrial cytochrome c oxidase subunit I (COI) gene, as pioneered by Hebert et al. (2003), offer a powerful tool for identifying organisms, especially arthropods. This approach addresses limitations inherent in traditional morphological identification, which can be time-consuming or impossible for immature life stages. The efficacy of DNA barcoding hinges on comparing generated sequences against well-curated public libraries containing species-identified sequences (Rimet et al. 2021). However, the accuracy of these libraries critically depends on the expert taxonomic identification of voucher specimens (Sheth and Thaker 2017; Cognato et al. 2020). DNA barcoding studies have revealed substantial undiscovered diversity, particularly within ‘dark taxa’ (Page 2016; Meier et al. 2025). When sequence comparisons fail to match known species, this may indicate the presence of species new to science or those not yet represented in reference libraries, thereby significantly reducing the number of individuals requiring classical taxonomic identification (Antil et al. 2023).

In this paper, we present the first global database resulting from a comprehensive study using field, morphological and molecular tools, compiled from bibliographic data and internet sources. Based on these data, we list 16 genera and 130 holoparasitic Orobanchaceae species along with their associated animals, and we identify general patterns and knowledge gaps limiting our understanding of holoparasitic plant-animal biology, ecology, and evolution at the global level. We focused on their associated animals, including mainly herbivores, pollinators, and seed dispersers, as well as carnivores and parasitoids, examining their development cycles at different stages and the relationships between them, to understand how multispecies and multi-layer interactions evolve and coevolve among species.

## Materials and methods

### Study area

The field study was conducted mainly by Renata Piwowarczyk for almost 20 years, 2005–2025, and partially assisted by Karolina (Ruraż) Wiśniewska, Alexander Fateryga, Attila Mátis, in Europe (Albania, Bulgaria, Austria, Czechia, France, Greece, Italy, Montenegro, North Macedonia, Poland, Portugal, Romania, Ukraine, Slovakia, Spain, Russia), western Asia (Georgia, Armenia, Azerbaijan, Turkey), and Central Asia (Uzbekistan, Kazakhstan). The observations were expanded with literature data and publicly accessible databases, finally obtaining data from 76 countries in Europe, America, Africa, Asia, and Australia (Suppl. material 3).

### Study species

Holoparasitic Orobanchaceae belong to 19 genera and include ca. 320 species (Sánchez Pedraja et al. 2016; POWO 2025; Thorogood et al. 2026). However, the taxonomic position of some species is not entirely clear. The dataset comprised representatives of all the major clades of holoparasites (*sensu*McNeal et al. 2013) inferred from previous molecular analyses of Orobanchaceae (Bennett and Mathews 2006; McNeal et al. 2013, and the latest modifications by Schneider 2016; Fu et al. 2017; Li et al. 2017; Piwowarczyk et al. 2021a). Namely, clade III Orobancheae Lam. & DC. includes: *Aphyllon* Mitch., *Boschniakia* C.A. Mey. ex Bong., *Cistanche* Hoffmans. & Link, *Conopholis* Wallr., *Epifagus* Nutt., *Eremitilla* Yatsk. & J.L. Contr., *Gleadovia* Gamble & Prain, *Kopsiopsis* (Beck) Beck, *Mannagettaea* Harry Sm., *Orobanche* L., *Phelipanche* Pomel, *Phelypaea* L., *Phacellanthus* Siebold & Zucc., *Xylanche* Beck, clade VI Buchnereae Benth. includes *Aeginetia* L., *Christisonia* Gardner, *Harveya* Hook., *Hyobanche* L., and clade V Rhinantheae Lam. & DC. includes *Lathraea* L.

**Figure 2.**
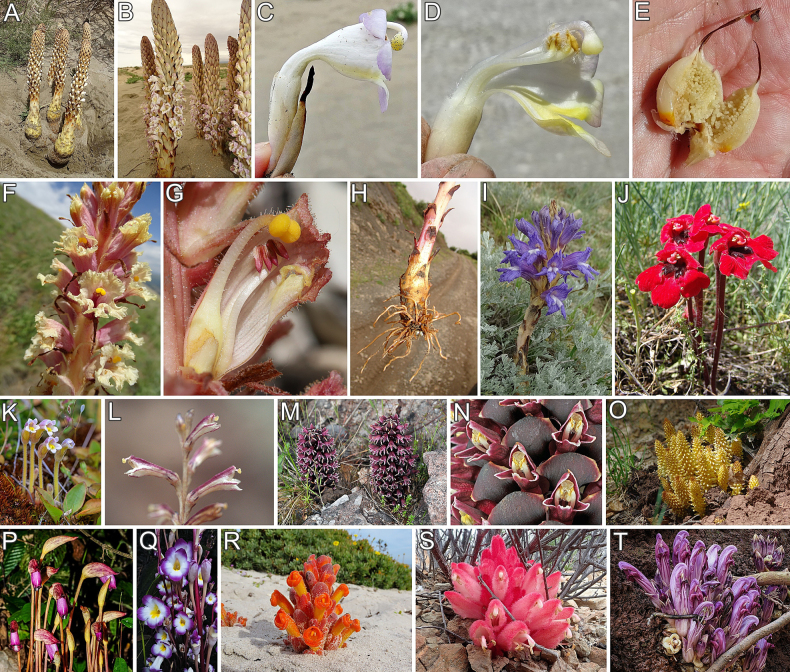
Representative members of the holoparasitic Orobanchaceae (general habit and some morphological details). **A–D**. *Cistanche flava*, Azerbaijan (**A**. General habit with a visible dug-out tuber; **B**. Inflorescences; **C**. Flower side view; **D**. Flower longitudinal section); **E**. Ovary with tiny seeds of *C. fissa*, Azerbaijan; **F–H**. *Orobanche kurdica*, Armenia (**F, H**) and Russia (**G**) (**F**. Inflorescence; **G**. Flower longitudinal section; **H**. Stem with scales, tuber and haustoria); **I**. *Phelipanche caesia*, Azerbaijan; **J**. *Phelypaea tournefortii*, Armenia; **K**. *Aphyllon purpureum*, USA; **L**. *Epifagus virginiana*, USA; **M, N**. *Kopsiopsis strobilacea* – general habit and flowers, USA; **O**. *Conopholis alpina*, USA; **P**. *Aeginetia indica*, India; **Q**. *Christisonia tubulosa*, India; **R**. *Harveya squamosa*, South Africa; **S**. *Hyobanche sanguinea*, South Africa; **T**. *Lathraea clandestina*, UK. Phot. R. Piwowarczyk (A–F, H–J), A. Fateryga (G), (K) photo by Radd Icenoggle from the iNaturalist website (https://www.inaturalist.org/photos/377791106), (L) photo by Tina Marie Camp Scheff from the iNaturalist website (https://www.inaturalist.org/photos/248176981), (M, N) photos by Gerry Carr from the iNaturalist website (https://www.inaturalist.org/photos/456813812, https://www.inaturalist.org/photos/456813810), (O) photo by dontomberlin from the iNaturalist website (https://www.inaturalist.org/photos/19371760), (P) photo by Rejoice Gassah from the iNaturalist website (https://www.inaturalist.org/photos/30116474), (Q) photo by Srinivasan Kasinathan from the iNaturalist website (https://www.inaturalist.org/photos/435427291), (R) photo by Jacobus Retief from the iNaturalist website (https://www.inaturalist.org/photos/232858192), (S) photo by almcinnes from the iNaturalist website (https://www.inaturalist.org/photos/162086523), (T) photo by mervyngreening from the iNaturalist website (https://www.inaturalist.org/photos/186526846); K–O, R–T: distributed under the terms of the Creative Commons CC BY-NC 4.0 license (https://creativecommons.org/licenses/by-nc/4.0/), P–Q: distributed under the terms of the Creative Commons CC BY 4.0 license (https://creativecommons.org/licenses/by/4.0/).

Their habitats are very diverse, ranging from open grasslands and arid zones to temperate, subtropical and tropical forests, from extremely dry to humid, at different elevational levels, from the sea level up to over 4,000 m a.s.l., from anthropogenic to semi-natural and natural ecosystems. These holoparasitic genera include many morphologically, ecologically, and geographically diverse species. They share a similar, completely obligate parasitism on the roots of diverse host species from 62 families (dominance of Asteraceae, followed by Fabaceae) and ca. 900 species of annuals, perennials to trees, monocotyledons to dicotyledons (Hatt et al. 2025). They do not form vegetative organs such as leaves, only reduced scales, while haustoria replace roots, and their appearance is limited to generative stems with short-lived impressive flowers/inflorescences, highly variable in colour and morphology (Fig. 2).

- Clade III Orobancheae [14 genera, ca. 244 species]. The most numerous species are included in the genera *Orobanche* (ca. 120) and *Phelipanche* (ca. 60), which occur worldwide, mostly in temperate regions of Europe, especially in the Mediterranean Basin, western and central Asia, northern Africa, less represented in the rest of Africa, America and Oceania. The next, moderately speciose genus is *Cistanche* (ca. 25 species), which occurs mainly in semidesert and desert habitats across Eurasia and Africa (especially the North). The genus *Aphyllon*, sister to the Old World genus *Phelipanche*, with more than 20 species is restricted to the New World, especially North America. The remaining genera are much less species-rich or geographically limited, often endemic. The genus *Phelypaea*, with three species, occurs in the Caucasus, and the Middle East, and rarely in Crimea and the Balkans. New World genera are also represented by *Epifagus* (1), and *Conopholis* (2), occurring mainly in temperate forests in northeastern North America. *Boschniakia* s.l., which formerly included four species, was recently divided into three genera with distinct geographical distributions: *Boschniakia* (1), *Kopsiopsis* (2), and *Xylanche* (1), their range extending into the farthest north and/or cold areas at high altitudes, e.g. woods and tundra, rocky slopes or cliffs, placed mainly in western North America and extreme northeastern Asia and the Himalayas. *Gleadovia* (5 species) occurs from the Himalayas to southern China. *Phacellanthus* comprises a single species distributed in the woods of eastern China, Taiwan, Japan, Korea, and Russia. *Mannagettaea*, with two species, is known from north-central and south-central China, Irkutsk Province, Qinghai, and Tuva. *Eremitilla* is a new genus with one recently described species endemic to south-western Mexico.

- Clade VI Buchnereae [4 genera, 67 species]. *Christisonia* comprises ca. 24 species distributed in tropical Asia, from India through south-eastern Asia and southern China to the Philippines. *Harveya* comprises about 28 species with a paleotropical distribution, widespread in eastern and southern Africa, Madagascar, and Comoros. *Hyobanche* has eight species and is endemic to southern Africa (from the Cape Floristic Region northward to Namibia). *Aeginetia* includes seven species found mainly in south-eastern and eastern Asia.

- Clade V Rhinantheae [1 genus, 5 species]. One holoparasitic genus *Lathraea* includes five accepted species, and its general native range comprises temperate Eurasia (Zhang and Tzvelev 1998; Wolfe and Randle 2001; Chung et al. 2010; Wester 2011; Piwowarczyk 2015; Sánchez Pedraja et al. 2016; Schneider 2016; Piwowarczyk et al. 2023; POWO 2025; Thorogood et al. 2026; Fig. 2).

### Plant and animal material and sample collection

Animals were collected using entomological hand nets or by hand from above-ground and underground parts of plants between 2005 and 2025. Some larvae, pupae and eggs of flies, beetles and thrips were also incubated at a temperature above 20 °C. Captured invertebrates were preserved in 95% ethanol for further taxonomic identification by specialists (based on standard morphological keys and molecular methods). The collected plant specimens were deposited mainly in the Herbarium of the Jan Kochanowski University in Kielce (KTC). The collected animal specimens were deposited mainly in the Jan Kochanowski University in Kielce, Entomological Collection of the University of Silesia in Katowice (DZUS), University of Rzeszów, University of Opole, Warsaw University of Life Sciences, the Comenius University in Bratislava, the Zoological Institute of the Russian Academy of Sciences in Saint Petersburg, while DNA extracts were deposited at the University of Lodz.

### Molecular identification through DNA barcodes

The DNA processing of 469 individuals was performed in the Department of Invertebrate Zoology and Hydrobiology, University of Lodz. The isolation was carried out from a single leg, piece of tissue, or whole specimen (according to the animal’s size) using the Chelex protocol (Casquet et al. 2012). The initial amplification, quality control and sequencing of the standard gene region for animal DNA barcoding (COI – cytochrome c oxidase subunit I), as described by Hebert et al. (2003) was performed using either approach presented in Rewicz et al. (2024) (standard Sanger sequencing method), or as presented in Srivathsan et al. (2021, 2024) employing Oxford Nanopore Technology (ONT). To increase the success of amplification and sequencing (Sanger method), we performed a second round of amplification for samples in which we initially failed to obtain sequences, and we used the cocktail of LCO1490-JJ/HCO2198-JJ (Astrin and Stüben 2008) + LEPF1/LEPR1 (Hebert et al. 2004) primers. We followed the reaction conditions from Hebert et al. (2004). In all cases processed through ONT, the COI was amplified with primer pair LCO1490-JJ and HCO2198-JJ (Astrin and Stüben 2008) attributed with 9bp tags (Srivathsan et al. 2024). The sequencing results were demultiplexed, and DNA barcodes were determined using the ONT barcoder 2.0 (Srivathsan et al. 2024). Sequences were checked to verify the presence of obvious contaminations using BLAST (Altschul et al. 1990) and were deposited in GenBank under accession numbers (PV416885–PV417158, PX981669–PX981749). We also deposited sequences in the Barcode of Life Data Systems (BOLD; http://v4.boldsystems.org (Ratnasingham and Hebert 2007)). We did this to obtain the Barcode Index Numbers (BIN) clustering together similar DNA sequences based on the genetic distance as tentative equivalents of species (Ratnasingham and Hebert 2013). Relevant voucher information, taxonomic classification, photos, and DNA barcode sequences are publicly accessible through the dataset DS-RPZAP (dx.doi.org/10.5883/DS-RPZAP) in BOLD.

### Diversity and distributional data

The database of more than 1370 worldwide observations was created based on the personally collected material (about half of the total data), and review of the available literature and online resources. It contains an alphabetically arranged taxonomic division (orders, families, genera, and species) of recorded animals. Unfortunately, identifying the animal to species level was often impossible, especially when using sources such as photographs. For each observation of an individual animal, data such as developmental stage, sex, role (if it was possible), parasitic plant species, plant organ, host plant, country, locality, name of observers/collectors, date, and reference source were included (Suppl. material 3).

We also used data from revised photographs from the first author’s field-work repositories and photographs submitted by researchers and amateurs or obtained from public internet databases, such as iNaturalist (https://www.inaturalist.org/); Plantarium (https://www.plantarium.ru/), Global Biodiversity Information Facility (https://www.gbif.org/), Flora of the World (https://floraoftheworld.org/), Index of Orobanchaceae (http://www.farmalierganes.com/Otrospdf/publica/Orobanchaceae%20Index.htm), and others.

Systematic division of parasitic plants was adopted according to Beck (1930) and Teryokhin et al. (1993) with some recently implemented taxonomic changes (e.g., Bennett and Mathews 2006; McNeal et al. 2013; Sánchez Pedraja et al. 2016; Schneider 2016; Li et al. 2017; Piwowarczyk et al. 2021a, 2024; POWO 2025); this scheme has been followed explicitly or implicitly by most researchers. Systematic division of animals was adopted according to GBIF (2025), as well as MolluscaBase (2025).

### Statistical analysis

Biodiversity metrics of identified fauna inhabiting parasitic plant were determined using PAST 4.03 (Hammer et al. 2021).

## Results

### Total diversity of animals interacting with Orobanchaceae

**Figure 3.**
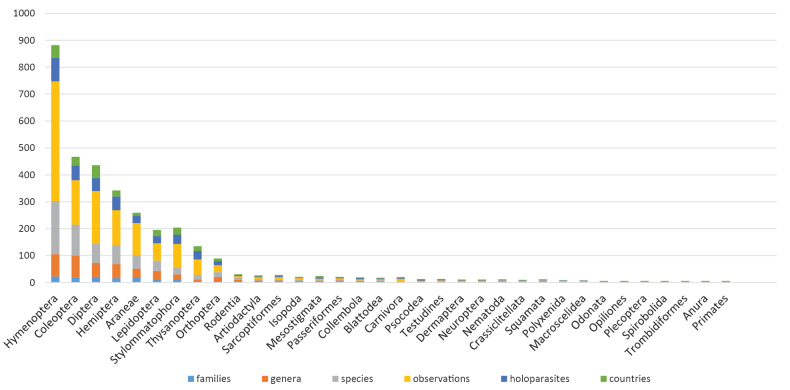
Total diversity of animals’ orders recorded on holoparasitic Orobanchaceae.

**Figure 4.**
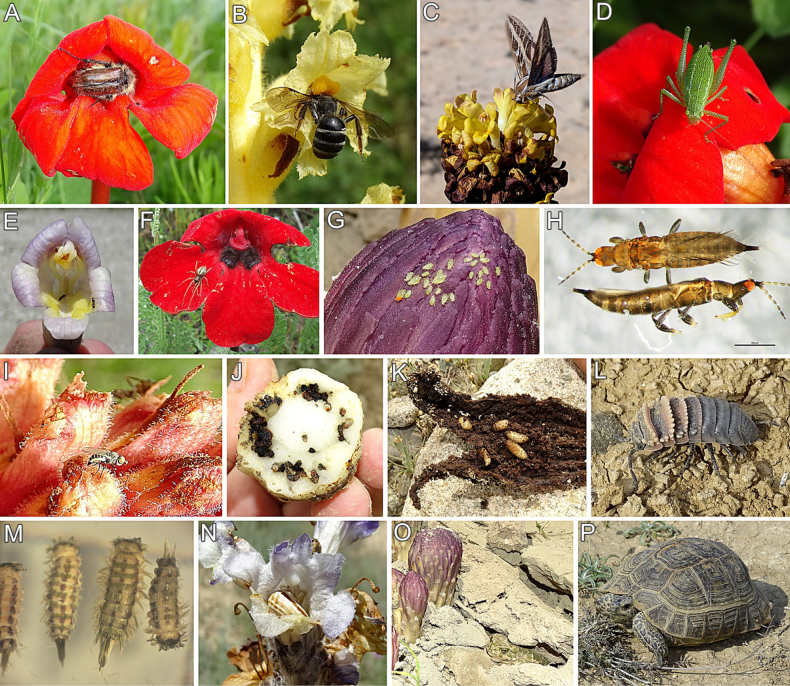
Representative species of orders of animals recorded on holoparasitic Orobanchaceae. **A**. *Pygopleurus psilotrichius* (Coleoptera, Glaphyridae) on *Phelypaea coccinea*, Georgia; **B**. *Lasioglossum zonulum* (Hymenoptera, Halictidae) on *Orobanche alsatica*, Poland; **C**. *Hyles livornica* (Lepidoptera, Sphingidae) pollinating *Cistanche phelypaea* s.l., Canary Islands; **D**. *Leptophyes* sp. (Orthoptera, Tettigoniidae) on *P. coccinea*, Georgia; **E**. *Plagiolepis perperamus* (Hymenoptera, Formicidae) on *C. flava*, Azerbaijan; **F**. *Cheiracanthium pennyi* (Araneae, Cheiracanthiidae) on *P. tournefortii*, Armenia; **G**. *Myzus persicae* (Hemiptera, Aphididae) on *C. flava*, Azerbaijan; **H**. *Neoheegeria cf. gigantea* (Thysanoptera, Phlaeothripidae) from *P. coccinea*, Georgia; **I**. *Phytomyza orobanchia* (Diptera, Agromyzidae) on *O. centaurina*, Poland; **J**. larvae of *Polyodaspis sulcicollis* (Diptera, Chloropidae) in the tuber of *C. fissa*, Azerbaijan; **K**. larvae of *Eumerus mucidus* (Diptera, Syrphidae) in the stem of *C. fissa*, Azerbaijan; **L**. *Hemilepistus klugii* (Isopoda, Agnaridae) under the tuber of *C. fissa*, Azerbaijan; **M**. *Propolyxenus argentifer* (Polyxenida, Polyxenidae) from the *O. laxissima*, Georgia; **N**. *Xeropicta derbentina* (Stylommatophora, Geomitridae) on *C. fissa*, Azerbaijan; **O**. Bufotes viridis subsp. sitibundus (Anura, Bufonidae) in the soil cracks created by the growing *C. flava*, Azerbaijan; **P**. *Testudo graeca* (Reptilia, Testudinidae) near *C. flava*, Azerbaijan. Phot. R. Piwowarczyk (**A, B, D–G, I–P**), C. Thorogood (**C**), K. Zubek (**H**).

We identified animals belonging to the categories of invertebrates (641 species = 96%; 1334 observations = 97%) and vertebrates (26 species = 4%; 37 observations = 3%). In total, these comprised 34 orders, 163 families, 434 genera and 667 species of animals. Our data are based on more than 1370 observations (including field records, molecular identifications, literature review, and open online databases), from 76 countries spanning almost every continent. The animals were recorded exploiting resources produced by 130 species (ca. 41% of all) belonging to 16 (84% of all) holoparasitic genera of the Orobanchaceae family (Table 1, Figs 3, 4, Suppl. materials 1–3). In the course of our research and review of available materials, we gained knowledge of the following holoparasitic genera (number of species in brackets): *Aphyllon* (9), *Aeginetia* (3), *Boschniakia* (1), *Christisonia* (3), *Cistanche* (13), *Conopholis* (2), *Epifagus* (1), *Harveya* (7), *Hyobanche* (5), *Kopsiopsis* (2), *Lathraea* (4), *Orobanche* (58), *Phacellanthus* (1), *Phelipanche* (17), *Phelypaea* (3), and *Xylanche* (1) (Suppl. materials 1–3). The genera most frequently exploited or visited by animals were *Orobanche*, followed by *Phelipanche* and *Cistanche*. Moreover, many genera and species, especially from the subtropical and tropical regions, have limited or no available data on their interactions with animals, which proves how insufficient the knowledge is on the studied subject. For example, no data were found on the diversity of animals visiting the following genera: *Eremitilla*, *Gleadovia*, and *Mannagettaea* (Suppl. material 1).

**Table 1.** Taxonomic structure and total diversity of animals recorded on holoparasitic Orobanchaceae.

Phylum/Class	Class/Order	Families	Genera	Species	Observations	Holoparasites	Countries
** Arthropoda **	Araneae	17	34	50	120	27	11
	Blattodea	3	3	3	3	3	3
	Coleoptera	19	81	115	165	54	33
	Collembola	2	3	3	4	4	3
	Dermaptera	1	2	2	2	2	2
	Diptera	21	52	72	195	48	48
	Hemiptera	16	53	70	130	51	22
	Hymenoptera	23	82	198	445	86	48
	Isopoda	2	4	4	7	2	2
	Lepidoptera	11	32	39	64	27	22
	Mesostigmata	1	4	4	5	5	5
	Neuroptera	1	2	2	2	2	2
	Odonata	1	1	1	1	1	1
	Opiliones	1	1	1	1	1	1
	Orthoptera	5	16	18	25	16	9
	Plecoptera	1	1	1	1	1	1
	Polyxenida	1	1	1	3	2	1
	Psocodea	1	1	2	3	3	3
	Sarcoptiformes	2	5	5	8	4	4
	Spirobolida	1	1	1	1	1	1
	Thysanoptera	2	9	18	57	31	18
	Trombidiformes	1	1	1	1	1	1
** Nematoda **	Nematoda	2	2	2	2	2	2
** Annelida **	Crassiclitellata	1	1	2	2	2	2
** Mollusca **	Stylommatophora	11	19	26	87	35	26
** Aves **	Passeriformes	3	4	4	5	3	2
** Amphibia **	Anura	1	1	1	1	1	1
** Reptilia **	Squamata	2	2	2	2	2	2
	Testudines	1	2	2	3	3	2
** Mammalia **	Primates	1	1	1	1	1	1
	Rodentia	4	6	7	7	4	3
	Carnivora	1	1	2	10	3	3
	Artiodactyla	2	5	6	6	4	4
	Macroscelidea	1	1	1	2	2	1
		**163**	**434**	**667**	**1371**	**130**	**76**

Among animals, the most numerous (percentage of overall observations/ percentage of overall species) were arthropods (91%/91%) with the majority of species belonging to the order Hymenoptera (32%/30%), followed by a significantly smaller number of Diptera (14%/11%), Coleoptera (12%/17%), Hemiptera (10%/10%), Araneae (9%/7%), Lepidoptera (5%/6%), Thysanoptera (4%/3%), and Orthoptera (2%/3%). The remaining orders were usually represented by only a few or several species (1%/3%) (Table 1, Fig. 4, Suppl. materials 1–3). Apart from arthropods, but still within invertebrates, snails and slugs from the order Stylommatophora were moderately common (6%/4%), while among vertebrates, mammals dominated (ca. 2%/2%), followed by low numbers of birds, and reptiles (Table 1, Figs 3, 4, Suppl. materials 1–3). The complete list of orders, families, and species is provided in Table 1 and Suppl. material 3.

The percentage of species of holoparasitic Orobanchaceae regarding particular orders of animals was as follows: Hymenoptera (20%), Hemiptera (12%), Coleoptera (12%), Diptera (11%), Stylommatophora (8%), Thysanoptera (7%), Araneae (6%), Lepidoptera (6%), Orthoptera (4%), Mammalia (4%). The most important animal orders recorded in the largest number of countries were: Hymenoptera (17% of recorded countries), Diptera (17%), Coleoptera (11%), Stylommatophora (9%), Hemiptera (8%), Lepidoptera (8%), Thysanoptera (6%), Mammalia (6%), Araneae (4%), and Orthoptera (3%). The most attractive plants structures were above-ground parts (95%), comprising flowers, and inflorescences with the stem part, and only 5% of observations of animals were made on underground parts (tubers and haustoria). Most often, these were underground representatives of the larval stages of Diptera (Agromyzidae, *Phytomyza*, Syrphidae, *Eumerus*, Chloropidae, *Polyodaspis*), Hemiptera (Aphididae, Pseudococcidae), Coleoptera (Curculionidae, Tenebrionidae, Scarabaeidae), Isopoda, Polyxenida, Collembola, and Crassiclitellata (Table 1, Suppl. materials 1–3).

### Invertebrates

#### Araneae and others Arachnida

The class Arachnida was represented by five orders, of which Araneae was by far the most numerous, supplemented with single observations coming from Sarcoptiformes, Mesostigmata, Opiliones, and Trombidiformes. Based on 144 observations across 28 holoparasitic plants coming from 13 countries, we confirmed a total of 22 families, 45 genera, and 61 species of Arachnida. In the most speciose order, Araneae, represented by 17 families, five families dominated: Thomisidae (5 genera, 8 species, 29 observations), Araneidae (8, 14, 22), Theridiidae (4, 6, 14), Salticidae (3, 4, 9), and Cheiracanthiidae (1, 5, 9). The remaining families (Anyphaenidae, Dictynidae, Philodromidae, Clubionidae, Lycosidae, Pisauridae, Gnaphosidae, Agelenidae, Linyphiidae, Liocranidae, Oxyopidae and Pholcidae) were represented by a single species (1–2) and observations (1–6). The genus *Cheiracanthium* was the most prominent in terms of both species’ richness and observation frequency, followed by *Araneus*, *Tmarus* and *Anyphaena*. More frequently observed (4–8) species were *Tmarus piger*, *Anyphaena accentuata*, *Mangora acalypha*, *Clubiona* sp., *Philodromus aureolus*, *Xysticus* sp., *Cheiracanthium* sp., *Enoplognatha ovata*, *Misumena vatia*, and *Thomisus onustus* (Table 1, Figs 3, 4, Suppl. materials 1–3).

#### Coleoptera

Based on 165 observations of 54 holoparasitic species from 33 countries the order Coleoptera was represented by 19 families, 81 genera, and 115 species. The most commonly observed families were Curculionidae, Scarabaeidae, Nitidulidae, Melyridae, Carabidae, Glaphyridae, and Tenebrionidae. The families with the highest number of species (5–15) were: Scarabaeidae, Curculionidae, Carabidae, Tenebrionidae, Nitidulidae, Meloidae, Melyridae, Chrysomelidae, Glaphyridae, and Latridiidae, while with the largest observations (7–24) were: Curculionidae, Scarabaeidae, Nitidulidae, Carabidae, Tenebrionidae, Melyridae, Glaphyridae, Meloidae, and Chrysomelidae. The most abundant in species (per 4 species) were the following genera: *Pygopleurus*, *Oedemera*, *Oxythyrea*, *Tropinota*, and *Dasytes*. The most frequently observed (4–9 observations) genera include: *Pygopleurus*, *Meligethes*, *Oxythyrea*, *Tropinota*, *Smicronyx*, *Oedemera*, and *Danacea*. The species that were observed most frequently (3–6 times) were: *Meligethes* sp., *Pygopleurus transcaucasicus*, *Oxythyrea funesta*, *Smicronyx cyaneus*, *Meligethes aeneus*, and *Tropinota squalida* (Table 1, Figs 3, 4, Suppl. materials 1–3).

#### Diptera

Based on 195 observations of 48 holoparasitic species from 48 countries Diptera was represented by 21 families, 52 genera, and 72 species. The most frequently observed family was Agromyzidae, with the dominant genus *Phytomyza* (83 observations, mainly *P. orobanchia*), and a less numerous Syrphidae, especially the genus *Eumerus* (11 species, 8 observations), Chloropidae (*Polyodaspis* with 2 species and 5 observations), and Sciaridae (Table 1, Figs 3, 4, Suppl. materials 1–3).

#### Hemiptera

Based on 130 observations of 51 holoparasitic species from 22 countries, we identified 16 families, 53 genera, and 70 species of hemipterans. The Aphididae family clearly dominated, in terms of the number of genera, species and observations (15, 28, 59, respectively), it was followed by Miridae (7, 11, 14), and Pentatomidae (4, 4, 13). The most frequently observed (6–9 times) genera were *Myzus*, *Aphis*, *Dolycoris*, *Macrosiphum*, *Rhopalosiphum*, and *Smynthurodes* with *Rhopalosiphum*, *Aphis*, and *Macrosiphum* also being the most numerous in species number (3–5). The most frequently observed (4–9 times) species were *Myzus persicae*, *Dolycoris baccarum*, *Smynthurodes betae*, *Aphis gossypii*, and *Macrosiphum* sp. (Table 1, Figs 3, 4, Suppl. materials 1–3).

#### Hymenoptera

The order Hymenoptera was the largest group among arthropods, and animals overall. We found 23 families, 82 genera, and 198 species from 445 observations of 86 holoparasitic species from 48 countries. Most observations were of the families Apidae (136), Formicidae (130) and, to a lesser extent, Halictidae (57), and Vespidae (38). The families with the highest species abundance were Formicidae (56, richest also on the generic level with 21 genera), Apidae (38), and Halictidae (30), with a slightly smaller share of Vespidae (16), Megachilidae (11), and Andrenidae (8) (Suppl. material 1). On the other hand, the most frequently observed genus was *Bombus* (97 times), followed by *Lasioglossum* (74), *Apis* (23), *Lasius* (22), *Myrmica* (13), *Polistes* (13), and *Dolichovespula* (12). The most numerous genera in terms of species were *Bombus* (22 species) and *Lasioglossum* (22), followed by *Andrena* (7), *Crematogaster* (7), *Formica* (6), and *Hylaeus* (6), as well as *Myrmica*, *Polistes*, *Tetramorium*, *Anthophora*, and *Osmia* (5 species each) (Suppl. material 1). The most frequently observed species was *Bombus* sp. (35 times), but this morphotaxon may represent a group of species, followed by *Apis mellifera* (23), *Lasius* sp. (14), *B. hortorum* (12), *Lasioglossum morio* (10), and *B. pascuorum* (9) (Table 1, Figs 3, 4, Suppl. materials 1–3).

#### Lepidoptera

Moths were represented by 11 families, 32 genera, and 39 species from 64 observations on 27 holoparasitic species from 22 countries. Among the families identified, the Noctuidae clearly dominated, in the number of genera, species and observations (12, 13, 16, respectively), followed by Sphingidae (4, 7, 23). Geometridae, Erebidae, Crambidae, and Pterophoridae were significantly less diverse, while the remaining families constitute single observations. The most frequently observed genus was *Hyles* (Sphingidae), with four species and 20 observations, as well as, but with less participation (2–4 times), Geometridae, *Helicoverpa*, *Daulia*, *Agrotis*, and *Scotia*. The most frequently observed species was *Hyles livornica* (17), and with significantly lower frequency *Helicoverpa armigera* (3), Geometridae sp. (2), and *Scotia segetum* (2), the remaining species were observed singly (Table 1, Figs 3, 4, Suppl. materials 1–3).

#### Orthoptera

Our research noticed five families, 16 genera, and 18 species from 25 observations of 16 holoparasitic plants from nine countries. The most frequently observed families (3–9 times) were Tettigoniidae, Acrididae, and Gryllidae. The most numerous families, in species and genus level, were Acrididae and Tettigoniidae. The most frequently observed and most diverse in species were the genera *Leptophyes* (dominant *L. albovittata*), *Phaneroptera* (mostly *P. falcata*), and *Oecanthus* (dominant *O. pellucens*) (Table 1, Figs 3, 4, Suppl. materials 1–3).

#### Thysanoptera

Thrips were recorded in relatively large numbers and belonged to two families, nine genera, and 18 species based on 57 observations of 31 holoparasitic plant species from 18 countries. Results confirmed the presence of two families (Phlaeothripidae with 36 observations, six genera with 10 species, and Thripidae with 13 observation, three genera with eight species), but there are additional observations of unidentified thrips assigned only to the order or family, which required further study. The most frequently observed (4–10 times) were the genera *Neoheegeria*, *Frankliniella*, *Haplothrips*, *Thrips*, and *Bolothrips*. The most numerous in species were the genera *Frankliniella*, *Haplothrips*, *Limothrips*, and *Thrips*, while most frequently observed (2–10 times) at the species level were *Neoheegeria gigantea*, *Frankliniella intonsa*, *Haplothrips reuteri*, *Bolothrips bicolor*, *Thrips atratus* (Table 1, Figs 3, 4, Suppl. materials 1–3).

#### Stylommatophora

Our analysis also provided data on gastropods (Mollusca, Gastropoda), where we identified one order Stylommatophora with 11 families, 19 genera, and 26 species based on 87 observations of 35 holoparasitic species from 26 countries. The highest number of observations involved Helicidae (37) and Geomitridae (18) followed by Arionidae (7), Gastrodontidae (6), and Dorcasiidae (5). The greatest species diversity was recorded in the families Geomitridae (5 species) and Helicidae (3). Among genera, the most often observed were *Theba* (30 observations) followed by *Xeropicta* (14), *Arion* (7), *Trigonephrus* (5), and *Zonitoides* (5). The most speciose genera (2–4 species) were *Arion*, *Monacha*, *Xeropicta*, and *Cepaea*. The most frequently observed (4–30 times) species were clearly *Theba pisana* and *Xeropicta derbentina*, followed by *Trigonephrus globulus*, *Zonitoides arboreus*, and *Arion subfuscus* (Table 1, Figs 3, 4, Suppl. materials 1–3).

#### Other orders

Source analysis also revealed several other invertebrate orders, usually represented by a single or a few taxa or defined generally at the higher taxonomic level, such as Blattodea (Blattellidae, Ectobiidae, *Ectobius* sp., Termitidae, *Microtermes* sp.), Collembola, Dermaptera (Forficulidae, *Forficula mikado*), Isopoda (Agnaridae, *Hemilepistus klugii* and *Protracheoniscus verhoeffi*, Ligiidae, *Ligidium* sp.), Neuroptera (Chrysopidae), Odonata (Libellulidae, *Brachydiplax* sp.), Plecoptera, Polyxenida (Polyxenidae, *Propolyxenus argentifer*), Psocodea (Lachesillidae, *Lachesilla*), Spirobolida (Pseudospirobolellidae, *Pseudospirobolellus cf. bulbiferus*), Nematoda, and Crassiclitellata (Lumbricidae) (Table 1, Figs 3, 4, Suppl. materials 1–3).

### Vertebrates

#### Aves

Analysis of literature sources showed that the birds were represented by one order Passeriformes, three families (Nectariniidae, Pycnonotidae, and Zosteropidae), four genera, and four species: *Nectarinia famosa*, *Anthobaphes violacea*, Hypsipetes amaurotis subsp. squamiceps, and *Zosterops japonicus*, based on five observations of three holoparasitic species from two countries (South Africa and Japan) (Table 1, Suppl. materials 1, 3).

#### Amphibia

Amphibians were recorded in only one observation of Bufotes viridis subsp. sitibundus (Anura, Bufonidae) (Table 1, Fig. 4, Suppl. material 3).

#### Reptilia

Based on five observations of five holoparasitic species from three countries (South Africa, Uzbekistan, and Azerbaijan), we recorded two orders, three families, four genera and four species. The reptiles identified belonged to the order Testudines, family Testudinidae, two genera, and two species of tortoises (*Chersina angulata*, *Testudo graeca*), as well as the order Squamata, family Gekkonidae (*Pachydactylus maculatus*), and Sphaerodactylidae (*Teratoscincus scincus*) (Table 1, Fig. 4, Suppl. materials 1, 3).

#### Mammalia

Mammals were represented by five orders (Primates, Rodentia, Carnivora, Artiodactyla, Macroscelidea), nine families, 14 genera, 17 species based on data from 26 observations of 10 holoparasites from eight countries. The family Ursidae was the most frequently observed (10 observations), followed by Bovidae (5), and Cricetidae (3). The most diverse taxa were in the families Bovidae (5) and Cricetidae (3). Bears were most frequently observed, especially *Ursus americanus* and *U. arctos*, and to a much lesser extent representatives of elephant shrews or cattle (Table 1, Suppl. materials 1, 3).

### Molecular identification through DNA barcodes

We obtained 355 COI sequences from 469 individuals (76% overall success). Sequences (seq) were assigned to 141 BINs, of which 24 were unique. The majority of the data originated from Poland (224 seq, 100 BINs, 5% unique), followed by Azerbaijan (92 seq, 24 BINs, 58% unique), Georgia (43 seq, 21 BINs, 33% unique), and Armenia (one seq, one BIN). We obtained two sequences from land snails, eight from Arachnida, seven from Diplopoda, 15 from Malacostraca and 329 from insects, and among them, representatives of nine orders, with dominants from Diptera (89 seq), Hemiptera (69 seq), Hymenoptera (50 seq) (Suppl. material 4). The molecular identification method was indispensable and helpful in identifying juvenile stages of arthropods, especially eggs, pupae, and larvae. We identified 98 taxa at the species level and 22 BINs at a genus-family level. The unique, unidentified BINs were mostly from the Caucasus (19 BINs, 84 individuals) and the rest from Poland (5 BINs, 11 records). Regarding existing BINs not identified to species level, we recorded 52 entries from 24 BINs from Poland, 18 records from seven BINs from Georgia, and 48 records from 10 BINs from Azerbaijan (Suppl. material 4).

### Geographical distribution and share of fauna inhabiting holoparasitic plants

**Figure 5.**
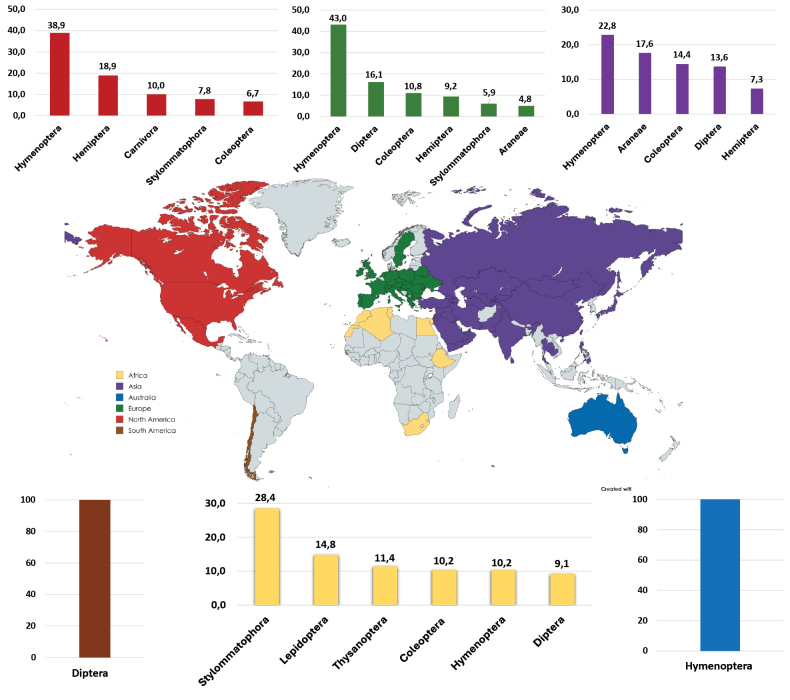
Distribution and share (%) of the fauna inhabiting parasitic plants (the world map from https://www.mapchart.net/world.html accessed 24.01.2026).

Hymenoptera are the most frequent visitor group across all continents, underscoring their global importance to the ecological functioning of parasitic plants (Fig. 5). Their consistent dominance highlights the key role of hymenopterans in mediating plant–animal interactions, particularly in relation to pollination and the exploitation of floral resources. North America exhibits a distinctive faunal composition, characterised also by a noticeable representation of mammals, especially Carnivora. This pattern highlights biologically interesting interactions between parasitic plants and vertebrates and suggests that, in some regions, parasitic plants may interact with higher trophic levels in ways that extend beyond classical insect-mediated relationships. Clear regional dominance patterns were evident on other continents. Unique dominance patterns, such as Diptera in South America (however based on single observations) and Stylommatophora in Africa, may reflect regional ecological adaptations and the role of local fauna in shaping plant–animal interactions, primarily involving native species. In contrast, most observations in Africa pertain to two invasive snail species (*Theba pisana* and *Zonitoides arboreus*), alien to the fauna of South Africa, found on the native *Hyobanche*, indicating their high adaptive capacity. Continental differences in the diversity of visitor groups further emphasise contrasting ecological strategies. Asia and Africa display a broader spectrum of faunal orders interacting with parasitic plants, suggesting more generalised interaction networks. Our findings are limited by a collection gap, specifically regarding the diversity of species found in South America and Australia. Overall, the observed biogeographic variation highlights the ecological flexibility of parasitic plants in forming associations with locally available fauna. These patterns suggest that parasitic plants may have evolved region-specific interaction strategies shaped by continental differences in faunal composition and environmental conditions, reinforcing their role as dynamic and context-dependent components of terrestrial ecosystems.

### Biodiversity metrics for fauna (orders and species) visiting holoparasitic plants (genera and species)

Suppl. material 5: table S3 presents a set of biodiversity indices describing the fauna (at the order level) visiting different genera of parasitic plants. Taxonomic richness and the number of observations varied markedly among the studied genera. The highest values of taxonomic richness were recorded for *Orobanche* (23 orders of animals), *Cistanche* (20), and *Phelipanche* (12), indicating a particularly high attractiveness of these genera to visiting fauna. The highest Shannon diversity values (H'), reflecting high overall biodiversity, were observed in *Cistanche* (H' = 2.300), *Phelipanche* (H' = 2.019), and *Orobanche* (H' = 2.018). These high Shannon indices were accompanied by low dominance values (Dominance_D), especially in *Cistanche* (0.133) and *Phelipanche* (0.165), suggesting a limited dominance of any single taxon. This pattern was further supported by high Simpson diversity values (1-D), reaching 0.867 and 0.836, respectively. Although *Orobanche* exhibited the highest taxonomic richness of animals and by far the largest number of observations of fauna (761), it showed slightly higher dominance (Dominance_D = 0.193) and the lowest evenness among the most diverse genera (Evenness_e^H/S = 0.327). This indicates a community structure in which a few taxa were highly abundant, while the remaining ones occurred at much lower frequencies. This trend was also reflected in the Berger–Parker index, which indicates a substantial contribution from the most abundant taxon to the total number of observations. Evenness indices (Equitability_J and Evenness_e^H/S) were highest in genera with low taxonomic richness of animals such as *Christisonia*, *Boschniakia*, and *Kopsiopsis*, indicating a relatively even distribution of observations among the few present taxa. However, in these cases, high evenness primarily results from the low complexity of the communities rather than from high biological diversity. Species richness indices, including Margalef, Fisher’s alpha, and the Chao-1 estimator, consistently highlighted *Cistanche* (Margalef = 3.621; Fisher’s alpha = 5.639; Chao-1 = 38) and *Orobanche* (Margalef = 3.316; Fisher’s alpha = 4.472; Chao-1 = 26) as the genera with the greatest potential diversity. The high Chao-1 values suggest that the true number of taxa associated with these genera may be underestimated, emphasising their role as key components of local ecological networks. The lowest biodiversity was recorded for *Xylanche*, represented by a single taxon and a single observation, resulting in maximum dominance and zero values for diversity indices. Similarly low Shannon and Simpson diversity values were observed for *Christisonia* and *Epifagus*, indicating highly simplified fauna visitors; however, this may be more indicative of a poor level of observation. In summary, the results demonstrate that *Cistanche*, *Phelipanche*, and *Orobanche* play a particularly important role in sustaining the faunal diversity associated with parasitic plants. However, differences among these genera suggest distinct ecological patterns, ranging from relatively even and well-balanced assemblages (*Cistanche*, *Phelipanche*) to highly species-rich but dominance-structured communities (*Orobanche*).

Marked differences in the proportional composition (%) of faunal orders were observed among parasitic plant genera (Suppl. material 5: table S4). The visitors were generally dominated by invertebrates, particularly insects, although vertebrates and non-insect invertebrates were locally important in several genera. Across all genera, Hymenoptera constituted the most frequent visitors, with a high mean contribution and clear dominance in many plant genera. Their relative abundance reached particularly high values in *Epifagus* (53.3%), *Kopsiopsis* (55.6%), *Lathraea* (54.2%), *Christisonia* (50.0%), and *Conopholis* (41.4%). The consistently high representation of Hymenoptera supports their central importance in the reproductive ecology of parasitic plants. Coleoptera formed the second most important group and showed pronounced dominance in several genera, most notably in *Phacellanthus* (50.0%) and *Phelypaea* (48.1%), but also contributed substantially to *Harveya* (25.0%), *Epifagus* (13.3%), and *Phelipanche* (13.6%) genera. This pattern suggests that beetles may play a significant role not only as floral visitors but also as herbivores or opportunistic feeders on parasitic plant tissues. Diptera were unevenly distributed among genera, reaching relatively high proportions in *Christisonia* (25.0%), *Cistanche* (19.5%), and *Orobanche* (13.9%). Their presence indicates the importance of generalist floral visitors and suggests that some parasitic plant genera may rely on a broader spectrum of pollinating or nectar-feeding taxa. Several genera were characterised by the dominance of non-insect invertebrates. *Hyobanche* was overwhelmingly dominated by Stylommatophora (61.1%), indicating a strong association with gastropods, possibly linked to humid microhabitats or ground-level floral structures. Similarly, *Boschniakia* showed a high contribution of Stylommatophora (30.8%) and Carnivora (38.5%), reflecting a unique relationship likely driven by habitat-specific or trophic interactions rather than pollination alone. Predatory arthropods such as Araneae were locally abundant, particularly in *Epifagus* (26.7%), *Phelypaea* (25.9%), and *Orobanche* (12.4%). Their presence suggests that parasitic plants may also function as structural microhabitats, supporting higher trophic levels and contributing to local food web complexity. Thysanoptera dominated the *Harveya* (37.5%) genus and were also notable in *Aeginetia*, *Cistanche* and *Phelypaea*, indicating possible specialisation or strong attraction to floral or vegetative tissues. Lepidoptera were most prominent in *Aeginetia* (26.7%) and *Cistanche* (13.7%), whereas Hemiptera reached high proportions in *Aphyllon* (45.8%), *Christisonia* (25.0%) and *Phelipanche* (20.8%), suggesting feeding on plant sap or associated host plants. Vertebrate visitors were recorded only sporadically and typically constituted a small fraction of the genera. Rodentia, Artiodactyla, and Passeriformes were recorded mainly in association with *Conopholis*, *Hyobanche*, and *Kopsiopsis*, respectively, highlighting intriguing and biologically meaningful interactions between parasitic plants and vertebrates. These observations suggest that parasitic plants may provide resources or microhabitats attractive to higher trophic levels, underscoring their underestimated ecological relevance beyond plant-insect interactions. Overall, the pronounced variability in faunal composition among parasitic plant genera indicates a spectrum of ecological strategies, ranging from highly specialised assemblages dominated by one or two faunal orders to more complex, multi-order communities. These differences likely reflect variation in floral morphology, phenology, resource availability, and habitat conditions, underscoring the multifaceted ecological roles of parasitic plants within terrestrial ecosystems.

Marked variation in biodiversity indices was observed among visitors associated with individual parasitic plant taxa (Suppl. material 6: table S6). Species richness (Taxa_S) and the number of observations varied widely among the analysed taxa, which was reflected in pronounced differences in diversity, dominance, and evenness indices. The highest biodiversity values were recorded for *Orobanche alsatica*, *O. laxissima*, *O. lutea*, *Phelipanche arenaria*, and *Cistanche fissa*. *Orobanche alsatica* exhibited the greatest species richness of animal visitors (Taxa_S = 79) and the highest number of observations (Observations = 88). This species also showed the highest Shannon diversity index (H' = 4.330), indicating exceptionally high diversity of associated visitors. The dominance index was extremely low (Dominance_D = 0.014), while the Simpson diversity index was very high (Simpson_1–D = 0.986), suggesting the absence of strong dominance by any single taxon. High evenness values (Evenness_e^H/S = 0.961; Equitability_J = 0.991) further confirm a well-balanced distribution of individuals among species. In addition, very high Margalef (17.420) and Fisher’s alpha (α = 372.6) values indicate outstanding species richness. *Orobanche laxissima* was similarly characterised by very high diversity, with a Shannon index of 4.039, high species richness (Taxa_S = 65), and a large number of observations (Observations = 95). Low dominance (Dominance_D = 0.021) and a high Simpson_1–D value (0.979) indicate a highly diverse and stable visitor assemblage, although evenness values (Evenness_e^H/S = 0.873; Equitability_J = 0.968) suggest a slightly less even distribution than in *O. alsatica*. High Margalef (14.050) and Fisher’s alpha (α = 90.73) values further support the high species richness associated with this species. *Orobanche lutea* also supported a highly diverse visitor community, with a Shannon index of 3.808, substantial species richness (Taxa_S = 47), and 52 observations. Very low dominance (Dominance_D = 0.024) and a high Simpson_1–D value (0.976) indicate a well-structured and diverse assemblage. High evenness (Evenness_e^H/S = 0.959) and elevated Margalef (11.640) and Fisher’s alpha (α = 236.3) values further emphasise the richness of associated taxa. High diversity was also observed in *Phelipanche arenaria* (H’ = 3.768; Taxa_S = 44; Observations = 46), which showed very low dominance (Dominance_D = 0.024), extremely high Simpson diversity (Simpson_1–D = 0.976), and near-perfect evenness (Evenness_e^H/S = 0.984; Equitability_J = 0.996). Similarly, *Cistanche fissa* exhibited high diversity (H' = 3.704) and species richness (Taxa_S = 48), with low dominance (Dominance_D = 0.030) and high Simpson diversity (0.970), although evenness was slightly lower (Evenness_e^H/S = 0.846), indicating some variation in relative abundances. Other species with relatively high Shannon diversity values included *Orobanche caryophyllacea* (H’ = 3.606), *O. alba* (H' = 3.370), *O. centaurina* (H' = 3.318), *O. hederae* (H' = 3.281), *Cistanche flava* (H' = 3.283), and *O. cumana* (H' = 3.481). These taxa were generally characterised by low dominance and moderate to high evenness, indicating well-structured visitor communities. Chao-1 estimates suggest that for the most species-rich plants (e.g. *O. alsatica*, *O. lutea*, *P. arenaria*, *C. fissa*), the observed species richness was likely close to the true richness, whereas for several moderately sampled taxa, the estimator indicates the potential presence of undetected rare taxa. In contrast, numerous parasitic plant species were represented by very low numbers of associated taxa and observations (Taxa_S ≤ 2; Observations ≤ 2), resulting in extreme values of dominance, evenness, and diversity indices. These cases likely reflect limited sampling effort or genuinely species-poor visitor assemblages and should be interpreted with caution when compared to well-represented host species. Overall, the analysed parasitic plant species form a clear gradient from species-poor, strongly dominated visitor assemblages to highly diverse and well-balanced communities characterised by high species richness, low dominance, and high evenness.

In Suppl. material 6: table S7, we compiled data on 126 parasitic plant taxa and 673 taxa of visiting fauna. Most parasitic plants were visited by a relatively small subset of very frequent invertebrates, supplemented by numerous rare or sporadic interactions with invertebrates and vertebrates, forming a varied distribution of interaction strengths. *Phytomyza orobanchia* is a fly primarily associated with Orobanchaceae parasitic plants, including many *Orobanche*, *Phelipanche*, and *Cistanche* taxa (recorded in 26 taxa), where it strongly reduces host condition due to herbivory. This highlights trophic specialisation on Orobanchaceae with the exploitation of multiple host taxa, making *P. orobanchia* a key antagonist in these networks. Hymenoptera pollinators, especially Apoidea bees, form a significant part of the visiting fauna, with many *Bombus* (recorded in 26 parasitic plant taxa) and *Lasioglossum* taxa (recorded in 15 taxa) achieving high percentage shares on individual hosts. Moreover, species like *Apis mellifera* (recorded in 14 taxa) occur across various hosts, underscoring their ubiquity. Among invertebrates, several aphid species (e.g. *Myzus persicae*) showed high proportional abundances on particular hosts, indicating a marked concentration of herbivory on single or a few parasitic plant species, whereas other phytophagous beetles reached only moderate, host-restricted values. Surprisingly, mammalian visitors (e.g. *Ursus arctos*, *Bos taurus*, *Capra hircus*, *Ovis aries*) and birds (e.g. *Nectarinia famosa*, *Zosterops japonicus*, *Hypsipetes amaurotis*) appear only sporadically and at low frequencies, typically confined to just one or a handful of plant species. This intriguing pattern points to opportunistic encounters, incidental grazing, trampling, or brief nectar/fruit raids rather than dedicated foraging, highlighting how parasitic plants can unexpectedly intersect with large vertebrate diets in specific habitats. Such rare but striking interactions add a fascinating layer to the ecological web, revealing the plants’ role beyond typical insect pollinators. The dataset also reveals a substantial representation of visitors, including ants (*Formica fusca*, *Lasius niger*, *Crematogaster sordidula*, *Dolichoderus quadripunctatus*), spiders (*Thomisus onustus*, *Misumena vatia*, *Pisaura mirabilis*), orthopterans (*Leptophyes albovittata*, *Oecanthus pellucens*), slugs and snails (*Ariolimax columbianus*, *Helix pomatia*, *Cepaea hortensis*, *Cochlicella acuta*, *Zonitoides arboreus*), and small mammals (*Peromyscus maniculatus*, *P. leucopus*, *Clethrionomys gapperi*, *Tamias striatus*). Their presence highlights the fact that parasitic plants are not only nectar or pollen resources, but also provide structural habitat, hunting platforms, shelter, or palatable vegetative tissues for a broad array of predators, scavengers, granivores and detritivores. Notably, the interaction matrix includes several specialist or locally restricted taxa, such as *Eumerus cistanchei* and *E. mucidus* on *Cistanche* and related hosts, or *Chyliza extenuata* associated with *Orobanche*. These species often show high percentages of a single or a few parasitic plant species, which may reflect evolutionary or ecological specialisation on these hosts, for example in larval development or adult feeding.

### Faunal interactions with parasitic plant genera: plant organs, developmental stages, and ecological roles

The distribution of plant organs visited by fauna revealed a strong preference for flowers, which were the most frequently used structures across nearly all parasitic plant genera (Suppl. material 5: table S5A). Flower visitation exceeded 60% in most cases and reached 100% in *Christisonia*, *Harveya*, *Kopsiopsis*, *Phelypaea*, and *Xylanche*. Other plant parts were visited less frequently but contributed to overall interaction diversity. Combined visitation of flowers and stems was particularly pronounced in *Phacellanthus* (61.1%) and occurred to a lesser extent in *Phelipanche* (5.9%), while underground stems and tubers (3.7%) represented important interaction sites in *Boschniakia* (25.0%) and *Cistanche* (23.9%). Interactions involving the whole plant or multiple plant parts were recorded mainly in *Hyobanche* (20.0%) and *Boschniakia* (8.3%), indicating more generalised patterns of habitat use. Fruits and seeds (2.9%) were visited in several genera, most notably in *Phacellanthus* (16.7%), *Aeginetia* (7.1%), *Epifagus* (6.7%), and *Lathraea* (5.4%). Among the studied genera, *Orobanche* exhibited the greatest diversity of visited plant structures, with fauna recorded on flowers, stems, fruits and seeds, underground organs, and combinations of multiple parts, suggesting a particularly complex pattern of structural interactions.

Regarding developmental stages, adult insects clearly dominated across all parasitic plant genera, accounting on average for 90.9% of records (Suppl. material 5: table S5B). In several genera, however, immature stages were also documented, indicating that parasitic plants may provide resources or suitable microhabitats for insect development. Larval stages were most frequent in *Aeginetia* (26.7%), *Cistanche* (14.6%), *Conopholis* (10.3%), *Phelypaea* (7.4%), and *Orobanche* (6.6%), while combined records of adults and larvae occurred in *Cistanche* (12.4%), *Epifagus* (7.1%), *Phelipanche* (6.6%), and *Orobanche* (3.1%). Juvenile stages were recorded usually in *Orobanche* (10.0%), *Harveya* (6.3%), *Cistanche* (4.9%), and *Phelipanche* (3.3%). Egg stages and exuviae were rare and restricted to a few genera, with eggs observed in *Orobanche* (2.3%) and *Phelipanche* (1.7%), exuviae in *Aphyllon* (4.2%) and *Orobanche* (0.4%), and records combining adults, larvae, and eggs limited to *Orobanche* (0.3%) and *Phelipanche* (0.8%). Overall, these patterns suggest that parasitic plants can support not only adult insects but also multiple developmental stages, highlighting their role as multifunctional habitats within ecological networks.

The analysis of faunal interactions associated with parasitic plant genera demonstrates clear genus-specific differences in functional structure (Suppl. material 5: table S5C). Across all genera, ten distinct ecological roles were recorded, indicating substantial functional diversity among interacting fauna. Phytophagous species (39.8%) constituted the dominant group in most genera, with particularly high proportions in *Boschniakia* (76.9%), *Aphyllon* (57.1%), and *Harveya* (53.8%). Anthophilous (28.3%) fauna formed the second most prominent group and were especially abundant in *Lathraea* (60.8%), *Kopsiopsis* (55.6%), and *Phelypaea* (55.6%), as well as in *Orobanche* (35.2%), underlining the importance of flower-associated interactions likely linked to pollination. Omnivorous species (17.5%) showed a heterogeneous distribution among genera, dominating entirely in *Christisonia* (100%) and occurring at notable levels in *Phacellanthus* (50.0%), reflecting flexible feeding strategies. Carnivorous fauna (6.8%) was less common overall but reached relatively high proportions in *Epifagus* (26.7%) and *Phelypaea* (25.9%), suggesting the presence of higher trophic interactions within these systems. Other functional groups including detritivorous, fungivorous, hemizoophagous, parasitoid, phytophagous granivorous, and resting fauna were generally rare and restricted to specific genera and observations. For instance, parasitoids were recorded only in *Orobanche* (3.4%), fungivores exclusively in *Orobanche* (0.3%), and resting fauna were most pronounced in *Harveya* (15.4%). Phytophagous, granivorous fauna represent a functionally complex group combining herbivory with seed feeding. Although generally rare, their presence indicates more intricate trophic interactions, as they may influence both vegetative tissues and reproductive output of parasitic plants. These low-frequency groups nonetheless contribute to the overall ecological complexity and highlight unique interaction profiles within certain parasitic plant genera.

### Parasite–animal interactions across evolutionary lineages of holoparasitic Orobanchaceae

**Figure 6.**
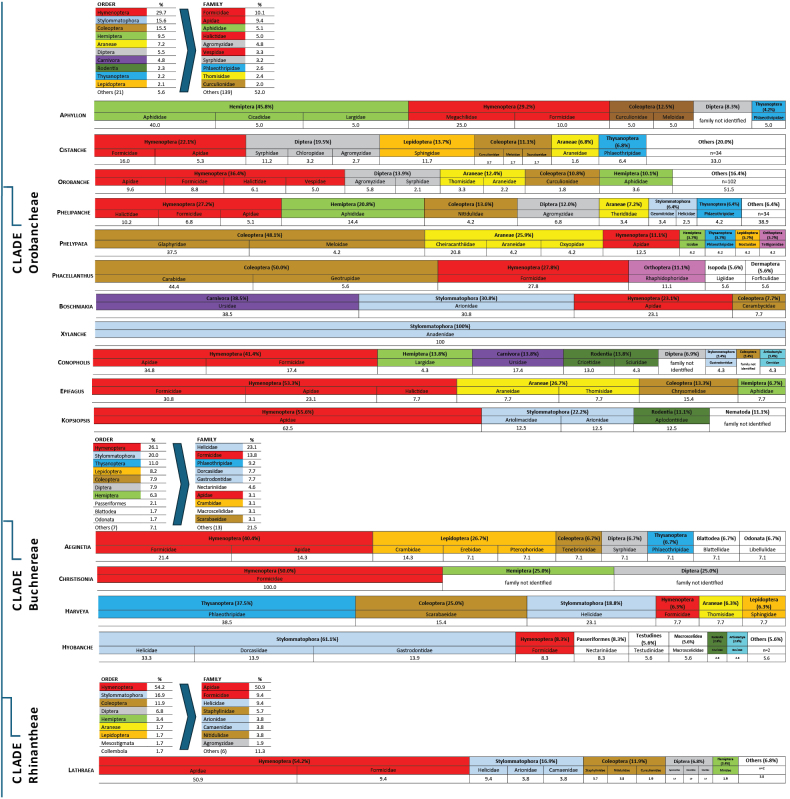
Taxonomic structure of animal visitors associated with phylogenetic clades (top) and with particular genera (bottom) of holoparasitic Orobanchaceae plants. For each genus, the relative contribution (%) of animal visitors is shown at the order and family levels. Consistent colors denote the same animal taxa occurring across different orders and families. Only the most frequent taxa are presented explicitly, while less abundant groups are pooled into the “Others” category.

Figure 6 illustrates differences in the taxonomic composition of animal visitors associated with genera of parasitic plants from the family Orobanchaceae, arranged into three phylogenetic clades. Across all clades, animal visitors were dominated by insects, although representatives of other animal groups, including arachnids, molluscs and vertebrates, were also recorded. At the order level, Hymenoptera constituted the most frequent visitors in the majority of plant genera, particularly within clades Orobancheae and Rhinantheae, followed by Coleoptera, Diptera and Hemiptera. In contrast, genera belonging to Buchnereae showed a relatively higher contribution of non-insect visitors, including Stylommatophora and vertebrate taxa. Analysis at the family level revealed marked variation among plant genera. Apidae and Formicidae were the most consistently represented families across clades, whereas other families occurred sporadically or were restricted to single genera. Despite this taxonomic heterogeneity, several animal families were shared among multiple genera, as indicated by consistent colour coding in the figure. Only the most abundant animal taxa are shown explicitly, while less frequent visitors are grouped into the “Others” category, highlighting dominant interaction patterns while retaining overall taxonomic breadth.

The clade Orobancheae (11 genera) exhibited the highest taxonomic richness and the most structured composition of animal visitors among the analysed groups. Insect visitors strongly dominated this clade, with Hymenoptera representing the most abundant order across most genera, followed by Diptera, Coleoptera and Hemiptera. Arachnids were also regularly recorded, particularly Araneae. At the family level, Apidae, Formicidae and Halictidae were consistently present across several genera, while additional families occurred with lower but recurrent contributions. In comparison with the remaining clades, Orobancheae exhibited the most detailed and taxonomically resolved visitor assemblages, supported by high numbers of identified families (*n*), which enabled explicit representation of numerous animal taxa.

Genera belonging to the clade Buchnereae (four genera) were characterised by a comparatively broad spectrum of animal visitors, including both invertebrate and vertebrate taxa. In addition to insects, a substantial contribution of non-insect visitors was observed, particularly Stylommatophora and mammalian orders such as Carnivora and Rodentia. At the order level, Hymenoptera and Coleoptera were frequently recorded but did not consistently dominate individual genera. At the family level, the composition of visitors was highly heterogeneous, with several families represented by single or infrequent records. This clade showed a relatively high proportion of taxa grouped into the “Others” category, indicating a diverse but weakly structured visitor assemblage.

In the clade Rhinantheae (one genus), the composition of animal visitors was dominated by insects, with Hymenoptera accounting for more than half of all records in this genus. Stylommatophora constituted the second most important non-insect group within this genus. Coleoptera and Diptera were present at moderate levels, whereas other arthropod orders contributed only marginally. At the family level, Apidae was the most prominent family, followed by Formicidae and several gastropod families, including Helicidae and Arionidae.

Overall, the three phylogenetic clades differed markedly in both taxonomic breadth and dominance structure of their associated animal visitors. Buchnereae was characterised by high heterogeneity and a relatively large contribution of non-insect taxa, Orobancheae by the highest diversity and repeated occurrence of key insect families, and Rhinantheae by strong dominance of Hymenoptera coupled with a consistent presence of gastropods.

The analysis of the provided data identified specific animal families that were unique to a single clade and did not appear in any others. In Orobancheae, there was a significantly higher level of exclusivity with 125 unique families. In the clade Buchnereae, the unique families were limited to Blattellidae, Cercopithecidae, Crambidae, Dorcasiidae, Gekkonidae, Libellulidae, Macroscelididae, and Nectariniidae. Conversely, the clade Rhinantheae exhibited the lowest level of exclusivity, containing only two families, Limoniidae and Staphylinidae, which were not found in the other groups.

A more detailed distribution of visiting fauna is also presented by the analysis of individual genera of holoparasitic plants (Fig. 6).

The animal assemblage associated with *Aphyllon* was strongly dominated by Hemiptera (45.8%), followed by Hymenoptera (29.2%), Coleoptera (12.5%), and Diptera (8.3%), with a minor contribution from Thysanoptera (4.2%). At the family level, Aphididae (40%) represented a substantial proportion of records, followed by Megachilidae (25%) and Formicidae (10%). Additional contributions from Curculionidae and Meloidae indicated the presence of beetles.

In *Cistanche*, Hymenoptera (22.1%) and Diptera (19.5%) were the most frequent visitors, followed by Lepidoptera (13.7%) and Coleoptera (11.1%), with additional records of Araneae (6.8%) and Thysanoptera (6.8%). The “Others” category accounted for 20.0% of records and comprised 34 families (33% of the total), each with a smaller share. The dominant identified families included Formicidae (16%), Apidae (5.3%), Syrphidae (11.2%), and Sphingidae (11.7%).

*Orobanche* exhibited a clear dominance of Hymenoptera (36.4%), followed by Diptera (13.9%), Araneae (12.4%), Coleoptera (10.8%), and Hemiptera (10.1%). The “Others” category constituted 16.4% of records and encompassed 102 families (51.5% of the total), reflecting high taxonomic richness. Prominent identified families included Apidae (9.6%), Formicidae (8.8%), Halictidae (6.1%), Vespidae (5.0%), and Agromyzidae (5.8%). The assemblage was highly structured and diverse.

The visitor spectrum of *Phelipanche* was dominated by Hymenoptera (27.2%), Hemiptera (20.8%), Coleoptera (13.6%), Diptera (12.0%), and Araneae (7.2%), with Stylommatophora (6.4%) and Thysanoptera (6.4%) also recorded. The “Others” category represented 6.4% and included 34 families (38.9%), indicating substantial additional taxonomic diversity beyond the principal families (Halictidae (10.2%), Formicidae (6.8%), Apidae (5.1%), Aphididae (14.4%), and Agromyzidae (6.8%)).

In *Phelypaea*, Coleoptera strongly dominated (48.1%), followed by Araneae (25.9%) and Hymenoptera (11.1%). Minor contributions were recorded for Hemiptera, Thysanoptera, Lepidoptera, and Orthoptera (each 3.7%). The prominent identified families included Glaphyridae (37.5%), Cheiracanthiidae (20.8%), and Apidae (12.5%).

The assemblage of *Phacellanthus* was dominated by Coleoptera (50.0%) and Hymenoptera (27.8%), with Orthoptera (11.1%), Isopoda (5.6%), and Dermaptera (5.6%) also present. The principal families included Carabidae (44.4%), Formicidae (27.8%), and Rhaphidophoridae (11.1%).

*Boschniakia* displayed an assemblage dominated by Carnivora (38.5%) and Stylommatophora (30.8%), with Hymenoptera (23.1%) and Coleoptera (7.7%) contributing to a lesser extent. Ursidae, Arionidae, Apidae, and Cerambycidae were the identified families, proportionally corresponding to the dominant orders. The assemblage was characterised by a substantial vertebrate component.

The assemblage associated with *Xylanche* (single observations) consisted exclusively of Stylommatophora (100%), represented entirely by Anadenidae (100%).

In *Conopholis*, Hymenoptera (41.4%) were most frequent, followed by Hemiptera (13.8%), Carnivora (13.8%), Rodentia (13.8%), Diptera (6.9%), Stylommatophora (3.4%), Coleoptera (3.4%), and Artiodactyla (3.4%). Apidae (34.8%) and Formicidae (17.4%) dominated among insects, while Ursidae (17.4%) and Cricetidae (13%) were the major vertebrate families.

The assemblage of *Epifagus* was strongly dominated by Hymenoptera (53.3%), followed by Araneae (26.7%), Coleoptera (13.3%), and Hemiptera (6.7%). Formicidae (30.8%), Apidae (23.1%), and Chrysomelidae (15.4%) were prominent among insects.

In *Kopsiopsis*, Hymenoptera (55.6%) dominated, followed by Stylommatophora (22.2%), Rodentia (11.1%), and Nematoda (11.1%). Apidae (62.5%) was the dominant family, alongside Ariolimacidae, Arionidae, and Aplodontiidae (each 12.5%).

The assemblage of *Aeginetia* was dominated by Hymenoptera (40.4%) and Lepidoptera (26.7%), with Coleoptera, Diptera, Thysanoptera, Blattodea, and Odonata each contributing 6.7%. Formicidae (21.4%) and Apidae (14.3%) were prominent among Hymenoptera, while Crambidae (14.3%) was the major representative of Lepidoptera.

*Harveya* was characterised by Thysanoptera (37.5%), Coleoptera (25.0%), and Stylommatophora (18.8%), with minor contributions from Hymenoptera, Araneae, and Lepidoptera (each 6.3%). Phlaeothripidae (38.5%), Scarabaeidae (15.4%), and Helicidae (23.1%) were the principal families.

The assemblage of *Christisonia* was dominated by Hymenoptera (50.0%), with Hemiptera and Diptera each contributing 25.0%. Formicidae was the only explicitly identified family.

*Hyobanche* exhibited dominance of Stylommatophora (61.1%), followed by Hymenoptera (8.3%), Passeriformes (8.3%), Testudines (5.6%), Macroscelidea (5.6%), Rodentia (2.8%), Artiodactyla (2.8%), and others (5.6%). At the family level, Helicidae (33.3%), Dorcasiidae (13.9%), and Gastrodontidae (13.9%) were prominent among gastropods, while Nectariniidae (8.3%) represented birds and Formicidae (8.3%) ants.

The assemblage of *Lathraea* was dominated by Hymenoptera (54.2%), followed by Stylommatophora (16.9%), Coleoptera (11.9%), Diptera (6.8%), and Hemiptera (3.4%). The “Others” category accounted for 6.8% and comprised two families (3.8%). Apidae (50.9%) was the most prominent family, followed by Formicidae and Helicidae (each 9.4%). The assemblage was strongly pollinator-centred, with moderate supplementary diversity.

Across plant genera, animal assemblages differed markedly in taxonomic composition. Hymenoptera and Coleoptera were the most frequently dominant orders across multiple taxa, whereas Hemiptera dominated in *Aphyllon*, Stylommatophora in several taxa (*Hyobanche*, *Xylanche*), and vertebrates contributed substantially in *Boschniakia* and *Conopholis*. Across taxa, Formicidae and Apidae were recurrent dominant families within Hymenoptera, whereas Aphididae frequently dominated Hemiptera. Among Diptera, Syrphidae and Agromyzidae were prominent, and among Coleoptera, Carabidae and Glaphyridae were major representatives.

## Discussion

### Visual, tactile and olfactory signals of Orobanchaceae in the evolution of animal–plant associations and adaptations

Numerous studies have found that the architecture of plants is a major factor determining the diversity of fauna exploiting their resources. Arthropods are especially responsive to these changes, as a single plant may serve as the entire habitat for a particular invertebrate species. Thus, even small changes in plants’ architecture can be impactful (e.g., Tews et al. 2004). Despite their typically rapid growth and short flowering period, holoparasitic Orobanchaceae possess diverse vegetative structures (with leaves replaced by reduced scales, and roots replaced by haustoria, and sometimes very long underground rhizomes) and especially generative structures where particular arthropods find food sources and favourable microclimate conditions for refuge, hunting, or development. Holoparasitic Orobanchaceae are very diverse in size, from plants of a few centimetres (geoflorous) up to ones over 1 m high. They grow singly or in clumps, with flower tubes from 1 to 10 cm in length. However, most of them have a very characteristic morphology expressed in the fleshy and succulent tissues, an extremely wide range of usually bright colours, and dense inflorescences or solitary, open flowers (Fig. 2). The underlying variation in plant chemistry is an essential factor in structuring the associated insect community (Visakorpi et al. 2019). Phytophagous insects have evolved finely tuned sensory systems for the detection of host cues, and a nervous system with a high level of spatio-temporal resolution. While plants have evolved to evade detection or defend themselves when attacked (Bruce 2015 and cited references; review by Serdo and Degaga 2023). Parasitic plants can be attractive food sources for herbivores (Heide-Jørgensen 2008). However, literature data focus mainly on hemiparasitic plants, especially mistletoes (their fruits, flowers, and foliage), as a food source, especially for birds and mammals (e.g., Watson 2001, 2009).

When analysing animal trophic relationships and diversity using holoparasitic Orobanchaceae, their phytochemical composition should be considered in detail. However, the phytochemistry of many species of holoparasites still remains unknown. These plants are rich in various phenylethanoid glycosides (PhGs), which are listed as their chemosystematic markers (Nicoletti et al. 1987). Some plants, like *Lathraea clandestina*, contain iridoid glycosides too, e.g. aucubin (black substances seen in decaying shoots and flowers), which are widely toxic or distasteful to insects and birds, and these substances undoubtedly act as deterrents to herbivores and pathogens (Atkinson and Atkinson 2020). Nectar from the parasitic plant *Orobanche colorata* contains substances with repellent properties, including thymol, n-benzyloleamide, azadirachtin, capsaicin, azatadine maleate, and andrachcinidine. For this reason, the parasite can also significantly modify the entomofauna of its host (Özenirler 2024). Moreover, the nectar of *L. clandestina* has an unusually high ammonia content (Prŷs-Jones and Willmer 1992). The presence of these toxic substances does not mean that there are not some animals which have adapted to it or which use the plant after flowering, when the amount of toxic substances decreases (see below).

The biological role of plant defensive chemicals can change over time (Bruce 2015). In addition to these dominant compounds, holoparasites from Orobanchaceae contain steroids, terpenoids, organic acids and their derivatives, alkaloids, lignans, and flavonoids (Shi et al. 2020). Phenylethanoid and iridoid glycosides may be considered specialised metabolites synthesised by parasitic species, because they are not typically found in host species (Scharenberg and Zidorn 2018). Although parasitic plants obtain all nutrients from the host, they have their own biosynthetic pathway and thus, the profile of host polyphenols differs qualitatively and quantitatively from that of the holoparasite (Piwowarczyk et al. 2020a, 2020b, 2021b; Lachowicz-Wiśniewska et al. 2024). PhGs play protective, antimicrobial, antistress, or resistance roles in plants (Jiménez and Riguera 1994). Extract of the tubers of *Xylanche himalaica* with a number of lignans and triterpenoids was found to possess strong feeding deterrent activity against the beetle *Tribolium castaneum* (Coleoptera, Tenebrionidae), one of common pests found in indoor food storage facilities (Cao et al. 2023). Some studies show that host plants can also negatively or positively influence herbivores feeding on hemiparasites through different pathways, not only phytochemical (Tjiurutue et al. 2016), and with great probability vice versa.

In holoparasitic Orobanchaceae, one can find many pigments, mainly anthocyanins, which are also responsible for their wide range of colours, including very intense ones which are attracting various animals (Fig. 2). Pigments also protect the plant against biotic and abiotic stresses, e.g., the anthocyanin content in the flowers of *Phelypaea tournefortii* is present in unprecedently large quantities (Piwowarczyk et al. 2020b).

In holoparasites, increased accumulation of elements, including heavy metals, and the production of specific metabolites, such as PhGs, may have an enhanced protective effect against drought, pests, herbivory, and disease damages (Boyd 2007; Mehdawi et al. 2011; Rascio and Navari-Izzo 2011; Bani et al. 2018; Piwowarczyk et al. 2021b). Although PhGs has been shown to deter feeding by generalist herbivores, many different plants (from other families) with known PhGs content are still attacked. In the holoparasitic Orobanchaceae, however, only one monophagous insect, the fly *Phytomyza orobanchia*, and probably several *Eumerus* species have demonstrated intensive feeding (despite the contained metabolites that repel other insects) and co-evolutionary adaptation. Closely related herbivore species often feed on closely related plants (Futuyma and Mitter 1996). The feeding range or possible monophagy of other insects requires further research.

Some mutualistic effects have been observed on trophic interactions in abiotic environments and other organisms, host and non-host plants, plant communities, herbivores, pollinators, and seed vectors. Their behaviour and diversity are often closely linked to the presence and abundance of parasitic plants, which are considered keystone species, and ecosystem engineers (Press and Phoenix 2005). Some authors consider parasitic plants to be herbivore analogues because interactions between parasitic plants and hosts often parallel those between herbivores and plants (Pennings and Callaway 2002). In the case of indirect effects, hosts weakened by a parasitic plant may be more susceptible to insect attack (Press and Phoenix 2005). Some works also highlight that plant and herbivore ontogeny interact to shape a specialist herbivore’s preference, performance and chemical defence (Quintero and Bowers 2018).

An interesting process that warrants mention is sequestration, defined as the uptake, accumulation, and eventual use of substances, especially toxins, by animals or plant species from other organisms (Mebs 2001; Erb and Robert 2016). Most reports deal with interactions between plants and herbivorous insects, but sequestration is also known in some gastropods, as well as in hemiparasitic (in most cases examined) and holoparasitic plants (Scharenberg and Zidorn 2018). Some data have presented evidence for the sequestration of alkaloids, flavonoids, and iridoid glycosides by some hemi- and holoparasites from their host species (Scharenberg and Zidorn 2018; Scharenberg et al. 2019). The parasite may also gain indirect benefits from the uptake of secondary metabolites, for example, alkaloids, such as a reduction in herbivory by insect larvae and increased visitation by pollinators (Adler 2000). Plants create bioactive natural chemicals to defend themselves. However, some insects detoxify and/or sequester the substances due to their co-evolution with plants. Herbivorous insects commonly use sequestration of chemical defences from host plants to avoid predation or metabolise sex pheromones, a phenomenon so far known from more than 250 insects. In most reported cases, toxic (but also non-toxic) secondary plant metabolites, such as glycosides and alkaloids, are sequestered by herbivorous insects (Dwarka et al. 2022), and these metabolites are also abundant in holoparasitic Orobanchaceae. However, these cases require further detailed research.

Insects, as pollinators, are often effectively guided by specific floral fragrances developed through co-evolutionary adaptations (Nishida 2014). Plants often emit hundreds of floral scents or floral volatile organic compounds (VOCs), and recent research on holoparasitic Orobanchaceae shows more than 130 VOCs per species, differing between species. The scent of some is aromatic, with a strong clove-like odour or very strong, fusel-like and earthy, mushroom fragrance, and attracts, or sometimes repels, various groups of insects. The spectrum of VOCs of 20 studied holoparasitic Orobanchaceae species (*Phelipanche* spp. emitted about 13% more VOCs than the *Orobanche* spp.) turned out to be unusually diverse, including 12 functional groups: alcohols, aldehydes, amines, hydrocarbons, aromatic hydrocarbons, carboxylic acids, esters, furans, ketones, phenols, sulphur compounds. In addition, many compounds found are known as semiochemicals with behavioural functions, such as kairomones, attractants, and pheromones, and some are known from no other plants so far. The authors also showed that the floral VOCs clearly separated the weedy, often invasive, holoparasites from the wild species (Tóth et al. 2016). Further research in Orobanchaceae, showing adaptations of specific pollinators or herbivores in correlation with the emitted chemical spectrum of flower scent, would be interesting. Nonetheless, insect attraction may be influenced by more overlapping factors. It is also unknown what physiological mechanisms in the host may influence VOC release by holoparasitic plants in holoparasitic plant-pollinator interactions (Casadesús and Munné-Bosch 2021).

Holoparasitic Orobanchaceae defend against herbivores using phytochemical substances (PhGs and other metabolites) and, to a lesser extent, some mechanical defences, such as nonglandular or glandular trichomes. Trichomes, as the first line of defence against herbivores and pathogens, may also complement the chemical defence of a plant by possessing glands with various substances. Most species in the holoparasitic Orobanchaceae are densely covered with trichomes, almost on the entire above-ground parts (stems, flowers), and, depending on the morphological part, trichomes can differ in density, length, and the presence of glands (Fig. 2). The trichomes are implicated in a variety of adaptive processes, including as a mechanical defence barrier against herbivores, for ion homeostasis maintenance, as a pollinator attractant, and for other signalling properties (Schilmiller et al. 2008, and references therein; Hassan and El-Awadi 2009). The results of recent studies on a few species of *Orobanche* s.l. have suggested the presence of terpenes and flavonoids in the glandular trichomes, and positive reactions to lignin, phenolic, lipid and suberin (Sacchetti et al. 2003; Hassan and El-Awadi 2009). The trichomes located on perianth elements and stamens of *Orobanche picridis* demonstrated the presence of four groups of metabolites, i.e., polyphenols (tannins, flavonoids), lipids (acidic and neutral lipids, essential oil, sesquiterpenes, steroids), polysaccharides (acidic and neutral polysaccharides), and alkaloids. The presence of such diverse bioactive compounds in *O. picridis* trichomes suggests their multifaceted role, e.g., against herbivores while attracting pollinators (Konarska and Chmielewski 2020).

Data collected during fieldwork confirmed that trichomes, especially those densely arranged on the stem or flower elements, act as traps for smaller insects. Dense and sticky hairs often had insects stuck to them, especially small flies, e.g., Sciaridae, Cecidomyiidae (Diptera) or Aphididae (Hemiptera) (Suppl. materials 1, 2). In this way, trapped small insects can also become easy prey for other arthropods. Similar conclusions, supporting our assumptions and observations, were previously published in other works (LoPresti et al. 2015 and cited references), which presented the hypothesis that glandular or hooked trichomes entrap insects onto plant surfaces and also may provide mutualistic predators. This phenomenon is likely a widespread convergent trait, known even in non-carnivorous plants, and may be a common indirect plant defence strategy in which specialised predators play the role of effective bodyguards despite the deterrence of generalist predators. LoPresti et al. (2015) compiled a list of insect-trapping sticky plants that included over 110 genera in 49 families, including two hemiparasitic genera in Orobanchaceae. In our opinion, the list can be supplemented with many species from the holoparasitic Orobanchaceae, especially *Orobanche* and *Phelipanche* and others (Suppl. material 3). In fact, many arthropod predators, such as insects belonging to the family Miridae or spiders, also have specific associations with plants containing glandular trichomes (Romero and Vasconcellos-Neto 2004; Sugiura and Yamazaki 2006), and capture prey that become adhered to these plant structures.

Further research is also needed to understand the potential involvement of arthropods or other animals in the feeding on, and perhaps indirectly in the dispersal of Orobanchaceae seeds. These seeds are one of the smallest in the world and are rich in numerous, mostly unsaturated, fatty acids (Ruraż et al. 2020), probably valuable in the diet of many organisms (see subsection below).

When looking at both phytochemical and architectural features of holoparasitic Orobanchaceae in terms of attracting and use by animals, attention is generally drawn to both above-ground and underground parts. The animals can be attracted by the succulent and fleshy inflorescences, stems and juicy tubers with haustoria, high-quality sap, relatively soft tissues, variety of colours, nectar and pollen, scent, indumentum, as well as the stiffness of the shoots, and numerous hiding places, and finally by the presence of other animals (Figs 2, 4, Suppl. material 2). These rewards attract specific visitors focused on particular attractants (food, such as nectar, pollen, tissues, and plant juices; the presence of shelters or other co-occurring animals, and various trophic relationships and symbioses between the animals (pollinators, herbivores, carnivores, and parasitoids)). Below, we discuss the most important aspects of these relationships.

### Mining phytophages

In general, phytophagous species constituted the dominant group (ca. 40%) in most genera of holoparasitic Orobanchaceae. The most recognised and most widespread monophagous insect that attacks holoparasitic Orobanchaceae (mainly the genera *Orobanche*, *Phelipanche*, and with a high probability also other genera), is the leaf miner fly *Phytomyza orobanchia* (Diptera, Agromyzidae) (Klein and Kroschel 2002) (Fig. 4, Suppl. material 2). So far, *P. orobanchia* has been reported from ca. 30 *Orobanche*, *Phelipanche*, and *Cistanche* species (Klein and Kroschel 2002; Černý et al. 2022; Suppl. material 3), but the real number of species is certainly higher. This fly is widespread, observed in more than 40 countries, mainly in southern Europe, western Asia, and northeastern Africa, as well as in South America and southern Asia (e.g., Manjunath and Nagarkatti 1977; Norambuena et al. 2001; Klein and Kroschel 2002; Dawah and Deeming 2025; Suppl. material 3). However, the most recent reports show its distribution range expanding further north, e.g., in Germany, Poland, and Slovakia (Piwowarczyk et al. 2018c; Černý et al. 2022). Our barcoding results show four BINs associated with *P. orobanchia*, or *Phytomyza* sp. These divergent clades need further taxonomic investigation, which may lead us to reveal new or undetected species, as we see in some recent studies regarding Agromyzidae (Boucher and Savage 2022). The larvae of *P. orobanchia* feed mainly on broomrapes. The larvae mine the stem, feed in seed capsules, and may reduce seed production by 30 to 80%, so it is considered one of the most effective biological control agents for invasive broomrapes (Klein and Kroschel 2002). Unfortunately, the flies attack not only invasive species of holoparasites, but also many rare and endangered holoparasitic plants (Suppl. material 3; Černý et al. 2022), causing a reduction in the number of their seeds and tissue necrosis. Further observations on the potential use of this fly as a natural enemy of invasive broomrapes are necessary. However, its effectiveness may be limited by agricultural practices and natural co-occurring organisms, such as bacteria, saprophytic fungi, predators, and parasitoids (Klein and Kroschel 2002).

Other important phytophages of holoparasitic Orobanchaceae belong to the genus *Eumerus* (Diptera, Syrphidae), one of the largest syrphid genera occurring mainly in Palaearctic. The larvae of its species are known to be destructive pests on various plants from numerous families (e.g., Amaryllidaceae, Asphodelaceae, Cactaceae, Liliaceae, Iridaceae, Apiaceae, Solanaceae, or Asteraceae) (Ricarte et al. 2017), attacking mainly their succulent and fleshy bulbs, stems, roots, fruits, or tubers. Some species of holoparasitic Orobanchaceae, especially *Cistanche*, have succulent and fleshy tissues of great mass (especially tuber and stem) and inhabit mainly desert and semi-desert habitats of Eurasia and North Africa (Piwowarczyk et al. 2019; Fig. 2), so it is not surprising that they have become a desirable host plant for these larvae. The larvae of 11 species of *Eumerus* (*E. ammophilus*, *E. arnoldii*, *E. cistanchei*, *E. compertus*, *E. mucidus*, *E. ovatus*, *E. turcmenorum*, and the newly described *E. alxaensis*, *E. seximaculatum* and *E. larvatus*), have been found in the tubers and shoots of a few *Cistanche* spp. (e.g., Stackelberg 1961; Waitzbauer 1976; Shaumar and Kamal 1978; Al-Khezraji et al. 1987; Piwowarczyk and Mielczarek 2018a; Song et al. 2020; Aracil et al. 2023; Suppl. material 3). Additionally, adult *Eumerus* spp., likely acting as a pollinator, were found on *O. alsatica* and *Cistanche* spp. flowers (Suppl. material 3). Generally, the genus *Cistanche* includes rare and endangered species and an intense attack by the larvae of this fly may threaten their existence. A good example is *C. armena*, an endemic and critically endangered species known only from a few localities in Armenia, which was attacked by the larvae of *E. mucidus* in almost 30% of the plant population, significantly damaging the stems and tubers (Piwowarczyk and Mielczarek 2018a; Piwowarczyk et al. 2019; Suppl. material 2). Additionally, we also observed numerous *E. mucidus* larvae in tubers and stems of *C. fissa* in many localities in Azerbaijan (Suppl. materials 2, 3, Fig. 4). Furthermore, more studies on the relation of *Eumerus* and *Cistanche* are definitely needed to confirm potential monophagy (Aracil et al. 2023), because many species of this syrphid genus are polyphagous, as well as being observed on other genera from Orobanchaceae in this respect. It should also be added that larvae often carry out hidden feeding, usually in tubers underground or in stems. Thus, without deliberate searches such as those done on *C. phelypaea* in the Iberian Peninsula by Aracil et al. (2023), it is difficult to determine the range and importance of these syrphids on plant species occurring within their distribution ranges.

The stem- and tuber-mining larvae of the chloropid flies *Polyodaspis sulcicollis* and *P. cf. rufescens* have also been observed in *Orobanche* and *Cistanche*, and are probably more common on these plants than has been reported so far (Suppl. material 3, Fig. 4). Sometimes, underground plant parts are very heavily infested with this fly. An interesting fact is that 70 adult flies were hatched from a piece of a *C. fissa* tuber (less than 50 grams; a month-long experiment with breeding this fly) from Azerbaijan. It is possible that the entire plant specimen could have contained several hundred larvae of this fly.

In all likelihood, further research will also bring more observations of beetle (Coleoptera) larvae using the fleshy and succulent stems and tubers (mainly underground ones) of these plants, and larvae of thrips (Thysanoptera) feeding on flowers, fruits, and stem tissues. So far, we have found Curculionidae and Scarabaeidae larvae boring in the underground tubers of *Phelipanche caesia* and *Cistanche fissa*. Manjunath and Nagarkatti (1977) observed larvae of Tenebrionidae boring in the shoots of *Orobanche cumana*, as well as adults of a few species from this family feeding on shoots superficially (Suppl. material 3). We also often observed other channels excavated in tubers and stems, but unfortunately, we could not determine which insect caused this damage.

We also found many mining thrip larvae in young flower ovaries and other tissues of many and various species of parasites (Suppl. materials 2, 3, Fig. 4), and we believe that these plants are common host and, for some insects, perhaps the only or preferred sites for the developmental and feeding stages. Moreover, *Neoheegeria gigantea* (Phlaeothripidae) has been recorded from several plant species (*Cistanche fissa*, *C. lutea*, *C. phelypaea*, *C. salsa*, *C. violacea*, *Phelypaea coccinea*) in large numbers in both sexes (Halperin and zur Strassen 2001; Minaei et al. 2007; Masarovič et al. in preparation; Suppl. material 3). It may thus be specialised for plants of this family, on which it not only feeds, but also reproduces (Masarovič et al. in preparation).

Moreover, we found *Sarcophaga cucullans* pupae (Diptera, Sarcophagidae) in underground tubers of *Cistanche fissa* in Azerbaijan (Suppl. material 3). There are also reports of Dermaptera and Termitidae (*Microtermes* sp.), which excavate tunnels in the shoots of *Orobanche cumana* in India (Manjunath and Nagarkatti 1977). Although some important agricultural pest species are polyphagous, there is a trend for phytophagous insects to become more specialised in the host plant use over time (Bruce 2015). Therefore, further observations in this respect in holoparasites are required. Ecological specialisation involves many complex interplays between plants and herbivores and other multitrophic interactions (Forister et al. 2012).

### Sucking phytophages

We noted a large group of phytophagous bugs that fed on plant sap. Due to their succulent and fleshy structure, holoparasitic Orobanchaceae are often attacked by usually polyphagous aphids (Hemiptera, Aphididae). Aphids attack the above-ground parts, stem and flowers, and the underground stem, tuber and haustoria. Aphids found most often on flowers and stems of holoparasites represent different species from dominant genera such as *Rhopalosiphum*, *Aphis*, *Myzus*, while aphids feeding on the tubers and haustoria belonged to *Smynthurodes betae*, *Rectinasus buxtoni* and rarely species of *Anuraphis*, *Brachycaudus*, *Uroleucon*, *Macrosiphum*, *Aulacorthum*, *Anoecia*, *Protaphis*, *Rectinasus*, *Rhopalosiphoninus*, *Geoica*, *Trama*, and *Uroleucon* (e.g., Borkowski and Dyki 2008; Holman 2009; Boukhris-Bouhachem et al. 2011; Piwowarczyk et al. 2018b; Suppl. materials 2, 3, Fig. 4). Numerous larvae and adults feed on broomrape sap, resulting in weakened or dead shoots, but potential use of these insects as a biological control agent for invasive broomrapes requires further study. Recent observations have shown that the *Aphis gossypii* may be a biological control agent, because it was found to completely stop the growth and flowering of broomrape plants (Borkowski and Dyki 2008); however, this species of aphid (as well as most of the aphids found) is quite polyphagous and is unlikely to fulfil such a role.

Apart from aphids, we also recorded a high number of other phytophagous Hemiptera that fed on the sap of holoparasites, e.g., *Orius niger* (Anthocoridae), *Aphrophora alni* and *Philaenus spumarius* (Aphrophoridae), *Neides tipularius* (Berytidae), *Aphrodes bicincta* (Cicadellidae), *Largus* sp. (Largidae), various species from the family Miridae (e.g., *Adelphocoris lineolatus*, *Calocoris* sp., *Dicyphus errans*, *Lygus pratensis*, *Megaloceroea recticornis*), *Eysarcoris aeneus* (Pentatomidae), *Coptosoma scutellatum* (Plataspididae), *Corizus hyoscyami* and *Rhopalus parumpunctatus* (Rhopalidae). *Dolycoris baccarum* (Pentatomidae) was often noted; interestingly, we documented the presence of all its stages on Orobanchaceae, from eggs through larvae to adults (Suppl. materials 2, 3). Pseudococcidae were noted on underground parts, such as tubers and haustoria, of *O. cumana* in Ukraine (Suppl. material 3) and *Dysmicoccus brevipes* in India (Manjunath and Nagarkatti 1977). We observed numerous individuals of various species of thrips (Thysanoptera) in flowers (Suppl. materials 2, 3, Fig. 4) where they fed on multiple flower parts.

During our observations, we saw many different small flies in Orobanchaceae flowers or entwined in their glandular hairs. Among insects from the order Diptera, we also recorded many predatory or herbivorous flies that suck sap from plants, causing them to wither, or feed on mycelium hyphae, e.g., Cecidomyiidae, Chironomidae, Chloropidae, Opomyzidae, Psyllidae, Sciaridae, and Syrphidae (Suppl. materials 2, 3).

### Florivores

Florivores are animals (invertebrates and vertebrates, but excluding pollen-eating pollinators) that damage and consume flower buds, flowers (partially or whole), and floral rewards before fruit and seed formation (Boaventura et al. 2022). Herbivory, specifically the less studied florivory (flower feeding), may influence plant breeding and plant population growth, as well as play a role in the evolution of flower traits. It may directly or indirectly affect plant attractiveness to pollinators (Moreira et al. 2019; Boaventura et al. 2022). Current knowledge suggests that florivory lies at the intersection of herbivory and pollination (McCall and Irwin 2006). A higher intensity of florivory is noted in tropical plants (Boaventura et al. 2022). Caterpillars are among the most common chewing florivores in the tropics, and recent research suggests that some species of herbivores might be specialised on flowers (Boaventura et al. 2022 and cited references). Caterpillars of moths were also recorded during our research (Suppl. materials 2, 3). The most intensive feeding by *Ammoconia senex* (Lepidoptera, Noctuidae) (Suppl. material 2) was observed in Georgia in a population consisting of several hundred individuals of *Phelypaea coccinea*. About 70% of the population was damaged to a greater or lesser extent, especially the flowers (single-flowered, red, large), petals, generative structures and immature fruits, which had numerous traces of gnawing and were riddled with holes. This caterpillar can eat an entire flower in a few minutes.

Other recorded florivorous caterpillars from Noctuidae (the family with the most frequently recorded florivory) were *Antitype chi* on *Orobanche lutea* in Austria, *Helicoverpa armigera* on *Cistanche phelypaea* in Spain and *O. cumana* in Azerbaijan and India, *Lacanobia contigua* on *O. picridis* in Poland, *Scotia segetum* on *O. cumana* and *O. crenata* in Serbia, *Zygaena* sp. (Zygaenidae) on *O. grenieri* in Georgia, *Orgyia dubia* (Erebidae) on *O. cumana* in Azerbaijan, Pterophoridae on *O. kotschyi* in Kazakhstan, and various Geometridae on a few *Orobanche* species (Suppl. materials 2, 3). *Spilosoma obliqua* (Erebidae) larva feeds voraciously upon the floral parts of *Aeginetia acaulis*, causing significant damage (Ray and Dasgupta 2010). The occurrence of lepidopteran herbivores was reported on *Aeginetia indica* and *Christisonia wightii* which parasitised sugarcane in the Philippines. These included *Platyptilia* sp. (Pterophoridae), which damaged 60–90% of flower buds and *Daulia* sp. (Crambidae) on the tender portion of the stem of *A. indica* (Lee 1931; Lee and Goseco 1932). As seen from the above examples, the caterpillars chose flowers that varied in both size and colour.

Florivorous beetles from the Curculionidae family (larvae and adults), such as *Smicronyx cyaneus*, *Hypera plantaginis*, *H. postica* (Zermane et al. 2001; Suppl. material 3), were found in Orobanchaceae flowers. Often larger beetles, feeding on pollen, damage and nibble the petals of flowers, e.g., *Tropinota*, *Oxythyrea* (Scarabaeidae), *Diabrotica*, *Luperomorpha* (Chrysomelidae), *Danacea*, *Dasytes* (Melyridae), *Mylabris* and *Hycleus* (Meloidae), Cerambycidae, Staphylinidae, Tenebrionidae, and others (see below) (Suppl. material 2). Florivorous orthopterans have also been observed, as have various species from e.g., Acrididae, Gryllidae or Tettigoniidae. There are also reports of *Kopsiopsis hookeri* and *Boschniakia rossica* from North America (e.g., Alaska) being damaged by nematodes nibbling on their flowers, as well as the slug *Arion subfuscus* (Arionidae) or banana slugs chewing on the flowers and mountain beavers eating them (Suppl. material 3). The gastropods, we found, require further observations as they often visit flowers and perhaps eat pollen, nectar or even stigma, as well as petals, e.g., *Arion* spp. (Arionidae) and *Zonitoides arboreus* (Gastrodontidae) (Suppl. materials 2, 3). *Meghimatium bilineatum*, an exotic slug of the Bonin Islands (Japan), was sometimes observed entering the flowers of *Orobanche boninsimae*, and feeding damage marks were observed to have probably been caused by them (Nishimura and Takayama 2022). Among vertebrates, there are observations of turtles eating flower petals and flower buds of *Hyobanche sanguinea* in South Africa (Turner and Midgley 2016) and *Cistanche flava* in Azerbaijan (Suppl. material 3; Fig. 4).

The morphology of holoparasitic plants may promote florivory more than in other non-parasitic plants, because they are leafless (small, reduced scales) and usually manifest as flowering inflorescences or, less frequently, single flowers on stems. The consumption of whole plants, including flowers, by vertebrates, is presented in the subsection below.

### Anthophilous animals and adaptations of holoparasitic Orobanchaceae to pollination

Flowers of the holoparasitic Orobanchaceae have evolved a plethora of strategies to attract pollinators, and many adaptations to cross-pollination can be observed (Figs 2, 4, Suppl. material 2). Not only pollinators but also herbivores and carnivores can respond to the same stimuli. Xenogamy primarily occurs in species that are chasmogamous, with cleistogamy being a rare occurrence (e.g., reported in *Epifagus*, *Cistanche*, and *Boschniakia* (Thieret 1971; Musselman 1980; Olsen and Olsen 1980). Flowers are campanulate or tubular (corolla tube has mostly 2–4 cm in length; through sometimes long-tubed, reaching even 7–9 cm in some species of *Harveya*, *Christisonia*, and *Gleadovia*). Usually, flowers are zygomorphic with a two-lipped corolla with landing platform, contrasting elements such as the corolla, stigma, anthers, folds, nectar guides and nectar rewards, as well as hidden UV-absorption patterns, scents or floral volatile organic compounds (VOCs) (Kjuit 1969; Tóth et al. 2016; Piwowarczyk and Kasińska 2017; León-Osper and Narbona 2022). There are many specific adaptations of flowers for pollination by insects, including: small flowers gathered in dense inflorescences, more rarely one-flowered but then usually with a bright and widely open flower (e.g., *Phelypaea*, *Aeginetia*, some *Aphyllon*); contrasting colouration and shine of the corolla and stigma; a wide range of various, often bright, colours; smell of the flowers; the lower lip of the corolla serving as an alighting place for pollinators (double-folded, often bright and covered with hairs or hairless or contrasting floral entry); production of nectar from coloured spots; and flowers open day and night (Fig. 2). The dense trichomes covering the folds of petals also accumulate falling pollen from the anthers and store it, as well as probably help gather pollen from other individuals from the trichomes on the bodies of pollinators. However, pollen grains were also observed on the hairless folds (Piwowarczyk and Kasińska 2017). Orobanchaceae is an eurypalinous family, with different types of pollen, usually inaperturate or tricolpate, with various sculptures of the exine (Piwowarczyk et al. 2015). In general, the adaxial side of the lower lip of flowers (landing platform for pollinators) of the species of Orobanchaceae can be divided into three types of macromorphology: Type I (typical for all *Phelipanche* and *Orobanche coerulescens*) with blue flowers with folds in the shape of distinct white or rarely yellow stripes covered with dense hairs; Type II (typical for *Phelypaea*) with red petals with black coloured nectar guides, with dark hairs; and Type III (typical for the remaining *Orobanche*, and *Boschniakia*, *Kopsiopsis*, *Conopholis*, *Epifagus*, *Mannagettaea*, *Phacellanthus*) with petals with shades of yellow, red and bronze colours, with folds of a different shape and size, mostly hairless, rarely with a few hairs (Piwowarczyk and Kasińska 2017; Fig. 2). Moreover, recent studies showed pollen, anther, stamen, and androecium mimicry (PASAM) and a hypothesis suggests that the system composed of the flowers possessing yellow UV-absorbing floral structures constitutes the world’s most speciose mimicry system (Lunau et al. 2024). The yellow UV-absorbing colour is probably one of the most recurrent visual signals in nature, which can be observed around the world among many angiosperm plants, as well as in the holoparasitic Orobanchaceae, e.g., anther mimicry like a fake anther, anther-like swollen folds or a dumbbell-shaped stigma (Osche 1983; Piwowarczyk et al. 2016; Lunau et al. 2024). *Harveya* has flowers with interesting, spurred anthers that block the passage to the nectar. The insect entering the flower will contact these spurs and produce vibration, causing it to shed pollen on the visitor (Marloth 1919–1932). Holoparasites are largely achlorophyllous, but some *Harveya* species also have chlorophyll concentrated in certain structures like petals, and the stigma, which probably appears only as an attractant for pollinators (Randle 2004).

So far, limited data could be found regarding the diversity of the pollinators of holoparasitic Orobanchaceae and most often evidence is based on accidental observations (e.g., Jones 1991; Ellis et al. 1999; Ollerton et al. 2007; Wester 2011; Tóth et al. 2016, 2026; Turner and Midgley 2016; León-Osper and Narbona 2022; Nishimura and Takayama 2022; Piwowarczyk et al. 2022; Özenirler 2024; Suppl. material 3). Our work is the first summary of this phenomenon based on long-term observations from many regions of the world, including diversity centres and origin regions of these plants, supplemented with data collected from literature and observations available on nature portals and databases (Suppl. materials 2, 3, Fig. 4). Apart from phytophagous species, anthophilous species formed the second most prominent group (average 28.3%), indicating that these plants often rely on animal visits for nectar or pollen collection (Suppl. material 5: table S5C). Below, we present the most important groups of anthophilous species, pollinators, or potential pollinators, showing them as a system of combined activities of diverse groups of organisms.

#### Bees and wasps

Bees (Hymenoptera, Anthophila) contain several functional groups, such as solitary and social, long-tongued and short-tongued, and vary in body sizes. The bilabiate flower is a one-way floral construction that, in most cases, offers nectar at its base for nototribic (dorsal, away from insect legs) pollen deposition, which evolved to protect pollen against pollen-collecting bees (Claßen-Bockhoff 2007). Such construction of flowers is also a perfect response to the biology and morphology of the bees (Claßen-Bockhoff 2007). Some authors indicate that, as bees have specialised on nototribic flowers, specific stiff hairs have evolved on the front surface of the head, with different inclinations, with examples mainly from Halictidae, Andrenidae, Megachilidae, and Apidae (Müller 1996; Thorp 2000; Gonzalez and Chavez 2004). Most bilabiate flowers are melittophilous, i.e., pollinated mainly by bees. As the distance to nectar, located deeply within the flower, increases in nototribic flowers, other pollinators with long proboscis have also evolved, apart from bees, including flies and moths (Westerkamp and Claßen-Bockhoff 2007). In addition to visual and olfactory adaptations for pollinators, several tactile adaptations related to the microstructure of flower elements, especially landing platforms, have also been described. The texture of a flower of Orobanchaceae may provide visual but also tactile signals for pollinators (Piwowarczyk and Kasińska 2017; Fig. 2). However, few and inconclusive works show these plants’ pollination biology. Some works show autogamy as common in the New World species, in contrast to more variable systems in the Eurasian taxa, with arguments showing autogamy, but also partial or dominated cross-pollination (Musselman et al. 1982; Jones 1991; Ellis et al. 1999; Rodríguez-Ojeda et al. 2013). Studies of *Orobanche* flower development indicate that the flowers have a mechanism that promotes autogamy at the end of flowering. In mature flowers at later stages of development, the style curves downward to lie in line with or slightly below the anthers, which must greatly facilitate self-pollination (Jones 1991). Jones (1991), conducting research in the British Isles (the northern limits of the broomrape range), suggests that autogamy occurs during the later stages of flowering, and that most British species can be both cross-pollinating and self-pollinating, but the degree of pollination of each is unknown. Our study did not focus on the effectiveness of xenogamy vs. autogamy or co-occurring processes, and this requires targeted future research. Like Jones (1991), we also noticed that bees and wasps penetrated deeply into the flower during their visit to search for the nectar, rather than to obtain pollen, which is much closer. Similarly to many authors who have shown that, for example, short-tongued *Bombus* species chew holes in many flower species as a short-cut to nectar (review by Irwin et al. 2010), we also observed holes chewed at the level of the nectar deposition in Orobanchaceae. To the best of our knowledge, nectar robbing was not previously observed in the holoparasitic Orobanchaceae (reviews by Irwin et al. 2010; Zhong et al. 2024).

Fieldwork observations and analysis of all sources revealed that the largest share in the primary pollination of the holoparasitic Orobanchaceae is undoubtedly played by Hymenoptera, especially by the bees in the families Apidae, Halictidae, Megachilidae, Colletidae, and Andrenidae and wasps of the family Vespidae (Suppl. materials 1–3, Figs 4, 6).

Anthophila were represented by both short-tongued and long-tongued bees. The former (Colletidae, Halictidae, Andrenidae, and Melittidae) had to enter the flower tube to get to the nectaries (they were usually characterised by small body sizes), while the latter (Apidae and Megachilidae – usually larger) only inserted their heads to obtain nectar. The bees were dominated by representatives of the Apidae and Halictidae families – primarily from the genera *Bombus* and *Lasioglossum*, i.e., mostly social bees, which usually have a much longer flight distance than solitary bees. In addition, a large caste of workers lives in their nests. These bees also have a longer flight period (often nearly the whole season) than solitary bees (1–2 generations). Solitary bees from the families Colletidae and Megachilidae or Andrenidae visited Orobanchaceae much less often. Most observed species belonged to polylectic bees, but some oligolectic species were also recorded, e.g., two species of *Hoplitis* (Megachilidae) oligolectic on *Echium* (Boraginaceae) (Sedivy et al. 2012) or *Rophites hartmanni* (Halictidae) oligolectic on *Ballota*, *Betonica*, *Mentha* (Lamiaceae) (Zimmermann et al. 2024).

Flowers of some Orobanchaceae, mostly of the genus *Orobanche*, attract a quite large proportion of vespid wasps, mostly social wasps of the genera *Dolichovespula*, *Vespula*, and *Polistes*; however, some species of solitary wasps (subfamily Eumeninae) were also recorded (*Euodynerus velutinus* and *Symmorphus gracilis* on *Orobanche alba*, *Eumenes pedunculatus* on *O. alsatica*, and *Allodynerus rossii* on *Phelipanche arenaria*) (Suppl. materials 2, 3). A similar composition of flower visitors was observed in some species of the genus *Scrophularia*, which largely attract more common social wasps but are also visited by the eumenine wasps as well (Robertson 1891; Brodmann et al. 2012; Navarro-Pérez et al. 2013; Fateryga 2020). The families Orobanchaceae and Scrophulariaceae belong to order Lamiales and flowers of *Orobanche* and *Scrophularia* have quite similar morphology. A small group of pollen wasps was represented by a single record of *Celonites tristiculus* on *O. amoena* (Suppl. material 3).

So far, insects, such as bees (including bumblebees) and wasps, were recognised as the major pollinators of Orobanchaceae (e.g., Baird and Riopel 1986; Joel et al. 2013; Piwowarczyk et al. 2022). *Lasioglossum frigidum* was reported in Japan as the primary pollinator of *O. coerulescens* (Nishimura and Takayama 2022). In Slovakia, the key broomrape pollinators are social wasps, bumblebees, bees from the Halictidae and Colletidae families, and hoverflies (Syrphidae). An interesting conclusion proposed by Tóth et al. (2016) is the change in floral VOCs correlated with the loss of pollination by social wasps and bumblebees in weedy broomrapes. The nectar of *Lathraea clandestina* has an unusually high ammonia content with a pH of as much as 11, rendering it unpalatable to birds and ants but apparently tolerated and pollinated principally by long-tongued bumblebees (*Bombus* spp.) (Prŷs-Jones and Willmer 1992; Atkinson and Atkinson 2020; Suppl. material 3). Similarly, other *Lathraea* species, such as *L. squamaria*, are most frequently pollinated by *Bombus* spp. (Suppl. materials 2, 3).

An interesting mutual relationship was presented between the parasitic plant *Aeginetia indica* and the bee *Ceratina flavipes* (Apidae) inhabiting the same host plant *Miscanthus sinensis* (Watazu et al. 2025). These results suggest that *C. flavipes* is the exclusive pollinator of *A. indica*, and this bee nests inside dead stems of *M. sinensis*, which is not only a host for the parasitic plant but also a nesting site for its pollinator. Therefore, *A. indica* may serve as an important autumn food resource for *C. flavipes* owing to its proximity to nests. Future studies should examine the relative importance of outcross-pollination by *Ceratina* bees in the reproduction of *A. indica*, which is assumed to be self-pollinating (Tiagi 1952).

#### Beetles

In our study, we demonstrated the significant importance of beetles in holoparasitic Orobanchaceae for the first time. Beetles are recognised as the oldest pollinators (Grimaldi 1999), since the early Cretaceous period, but despite this, they are often overlooked as pollinators compared to bees (Muinde and Katumo 2024). Pollen is an important food resource (Faegri and van der Pijl 1979) for many groups which shifted to pollen feeding independently (e.g., Hunt et al. 2007). Additionally, flowers are also mating sites for these animals (Barth 1985). More than 30 families of beetles have been documented as visiting flowers, and many plant families have prominent beetle pollinators (cantharophily), such as Melyridae, Scarabaeidae, Nitidulidae, and Chrysomelidae (Gottsberger 2016; Kirmse and Chaboo 2020). Our observations confirmed the presence of species belonging to the above-mentioned families on the flowers of holoparasitic Orobanchaceae. Almost all the found beetles were anthophilous, i.e. feeding on pollen and nectar in flowers of various holoparasitic species, especially from the genera *Orobanche*, *Phelipanche*, *Cistanche*, *Phelypaea* in various mainly Eurasian countries (Suppl. materials 2, 3, Fig. 4). Simultaneously, beetles such as Chrysomelidae, Scarabaeidae, Tenebrionidae, and Curculionidae were among the insects that interacted with early angiosperms around 140–100 million years ago (Thien et al. 2009), and we also found species representing these families in flowers of Orobanchaceae (Suppl. materials 1–3, Fig. 4). In addition, we also recorded melitophagous or partially anthophilous representatives from other families, including Cerambycidae, Dasytidae, Latridiidae, Meloidae, and Oedemeridae. Among anthophilous beetles, the most frequently recorded were: *Danacea nigritarsis*, *Dasytes niger* (Melyridae), *Hycleus scabiosae*, *Meloe*, *Mylabris*, *Teratolytta* (Meloidae), *Meligethes* spp., *Sagittogethes distinctus* (Nitidulidae), various *Oedemera* (Oedemeridae), and *Oxythyrea* and *Tropinota* species (Scarabaeidae) (Suppl. materials 1–3, 5: table S5C). While feeding on pollen, many beetles, e.g., *Tropinota* or *Oxythyrea*, damaged flowers when they could not fit into them.

An interesting and quite rare phenomenon among the holoparasitic Orobanchaceae is the occurrence of intensely red flowers with black spots that are single, large and wide open, such as those known from the genus *Phelypaea* (*P. boissieri*, *P. coccinea* and *P. tournefortii*; Figs 2, 4). During our research, we observed a surprising co-evolutionary phenomenon, in which these species were pollinated and frequently visited by beetles from Glaphyridae, *Pygopleurus* and *Eulasia*. These glaphyrids are attracted by the dark spots that mimic beetles, and this is very likely a case of deceptive pollination through sexual mimicry. The two black spots in *Phelypaea* genus seem to be a case of even more advanced mimicry (mimicking beetle elytra wings), because they are 3-dimensional dummies, as the folds are raised and hairy, eliciting mating attempts from the male beetles. Also worth emphasis is the fact that four species of *Pygopleurus* were observed abundantly on species of *Phelypaea* across their entire range, from Greece, through Turkey up to the Caucasus, Armenia and Georgia (Fig. 4). Moreover, this genus of beetles was not recorded in other species of holoparasitic Orobanchaceae, with one exception of an observation on a dry *Orobanche* in Georgia (Suppl. material 3). Other studies have shown that some species of Glaphyridae (i.e., *Pygopleurus* and *Eulasia*) are dominant pollinators of plants with red bowl-shaped flowers (Dafni et al. 1990). However, in *Eulasia* and *Pygopleurus*, colour preferences may vary among red, yellow, violet and white, sometimes even among related species (Sabatinelli et al. 2020). The presence of red-sensitive photoreceptors enables the strong association of *Pygopleurus* species with red flowers (Martínez-Harms et al. 2012). In addition, we found an observation of three individuals of *Eulasia cf. chrysopyga*, on a *P. coccinea* flower from Georgia (Suppl. material 3). A separate study involving several *Orobanche* plants with varying shades of red flowers suggests that the bee-avoidance hypothesis does not apply to red-flowered *Orobanche* species in the Mediterranean Basin. Those results suggested that red-flowered species are pollinated mainly by hymenopterans, and occasionally by dipterans, coleopterans or lepidopterans (León-Osper and Narbona 2022). However, the *Orobanche* species used in above work were not typically red, and their flowers were small and gathered in dense inflorescences, unlike *Phelypaea*. Only occasionally did we observe pollination of *Phelypaea* by representatives of bees, *Eucera* (Hymenoptera, Apidae) (Suppl. materials 2, 3).

#### Syrphids

After Hymenoptera, flies are probably the second most common insects visiting flowers, and especially hoverflies are considered important pollinators (Chang et al. 2024). Also, pollination by flies (myophily) is economically important for both cultivated and wild plants. Adults of hoverflies are anthophilous with diverse morphology, from large species imitating bumblebees, bees to hornets to small species mimicking solitary bees or wasps (Howarth et al. 2004; Penney et al. 2012). Flowers are vital to hoverflies for a different reason than bees; they provide nectar as a food source and pollen is required for ovarian development (Schneider 1948). This difference means that hoverflies are not restricted to a limited home range and may carry pollen over longer distances than bees (Rader et al. 2011), facilitating high levels of gene flow between distant plant populations (Doyle et al. 2020). During our research, we observed several species of Syrphidae, like *Episyrphus balteatus*, *Eumerus* spp., *Paragus* spp., *Pipizella* sp., *Syrphus torvus*, *Xanthogramma* sp., *Sphaerophoria scripta*, that may be important or complementary as pollinators (Suppl. materials 2, 3).

#### Moths

The Lepidoptera (butterflies and moths) have dichotomous relationships with plants, primarily herbivores as larvae, and later pollinators as adults. Two main families of moths stand out as pollinators: Sphingidae and Noctuidae (Inouye 2011). Moths have an important role as pollinators (day and night active moths), but nocturnal interactions are often overlooked, presumably because of the difficulty of conducting observations at night (MacGregor et al. 2015; Buxton et al. 2018). During our studies, we observed several adult moths on Orobanchaceae flowers, mainly from families active at night, such as Noctuidae (e.g., *Autographa gamma*), Sphingidae, Gracillariidae (*Euspilapteryx auroguttella*), and Scythrididae (*Parascythris muelleri*) (Suppl. materials 2, 3). However, moths constituted a small percentage compared to other groups of pollinators. The flowers of holoparasitic Orobanchaceae also remain open at night. However, further, targeted and nighttime research is necessary to understand the range and importance of moths in the pollinating these plants. Based on the photographs (taken during the day), we also established that a few species of hawkmoths of the genus *Hyles*, especially *H. livornica*, are important pollinators of the genus *Cistanche*, mainly in the Mediterranean Basin to SW Asia (Suppl. materials 2, 3, Fig. 4). Another interesting nocturnal observation comes from the deserts of Uzbekistan, where a moth (Sphingidae) was found at the flowers of *Cistanche mongolica* (Suppl. material 3). This plant species has whitish-pink flowers with a distinct scent, which is typical of the plants pollinated by nocturnal moths (Faegri and van der Pijl 1979). There are also reports of hawkmoths, such as *Hyles chamyla* and *H. hippophaes*, flying around *Cistanche* flowers after sunset (Pittaway 2025). The convolvulus long-tongued hawkmoth *Agrius convolvuli* (Sphingidae) was identified as the most important pollinator of African plants with very long-tubed flowers. Patterns of convergent evolution also include *Harveya speciosa* (and probably *H. capensis*) with 7–9 cm long flower tubes and white flowers with abundant nectar blooming also at night (Randle 2004; Johnson and Raguso 2016).

#### Orthoptera

During our research on flowers, we also observed various developmental stages of Orthoptera, mostly larvae and rarely adults, attracted also by nectar and pollen, especially from the Tettigoniidae family (*Leptophyes albovittata*, *Phaneroptera* spp., *Poecilimon* sp.), occasionally from the Gryllidae family (*Oecanthus pellucens*) (Suppl. materials 2, 3, Fig. 4). The role of orthopterans in pollination of plants is a little studied phenomenon, and we have valid data on it mainly from the tropical regions (Nagy et al. 2023; Nagy et al. 2023 and cited references). Orthopterans, mostly known as herbivores or even predators, are not efficient pollinators. However, their wide distribution and relatively high abundance may make them an important member of flower-visiting- and even pollinator assemblages (Nagy et al. 2023). The flower-visiting habit of temperate zone orthopterans is mainly unknown, only some sporadic data can be found on it. An interesting study was performed with volatile traps of insect pests in western Ukraine which proved significant attractivity (especially for *Phaneroptera* and *Leptophyes* species) of compounds of flower scents in the temperate zone (Nagy et al. 2023).

#### Other minute pollinators: thrips, ants, and flies

The feeding of thrips on plant tissues and their ability to transmit viral diseases have usually placed these insects in the category of pests. However, their high abundance and short- and long-distance movement capability may also make them effective, although secondary or complementary, pollinators (Moog et al. 2002; Eliyahu et al. 2015). Before the appearance of angiosperms, thrips were among the first groups of insects, along with some beetles and flies, to pollinate plants (Willmer 2011). Most likely, pollination in thrips has evolved through pollen and other nutritious secretions parasitism, and attraction to heat (Willmer 2011; Peñalver et al. 2012). Additionally, pollination can be enhanced by the floral chemistry of plants (e.g., *Sambucus nigra*) that attracts thrips (confirmed in *Thrips major*) to flowers at certain times. This can create mutualistic relationships that are beneficial for both the thrips (oviposition, feeding) and the plants (pollination) (Scott-Brown et al. 2019). Furthermore, although *Euphrasia willkommii* (hemiparasitic Orobanchaceae) is commonly observed to be self-pollinating, their tubular flowers were visited by low numbers of thrips, and their pollination potential was discussed (Gómez 2002). Numerous individuals of various species of thrips (Thysanoptera, Thripidae, Phlaeothripidae), especially *Frankliniella intonsa*, *Bolothrips* spp., *Hoplandrothrips* spp., *Limothrips* spp., *Thrips atratus*, or *Neoheegeria gigantea*, were observed in flowers (Suppl. materials 2, 3, Fig. 4), probably feeding not only on pollen, but also on other parts of flowers. At the same time, flowers might work as shelter and even a breeding place for these insects, but this requires further research. We are unable to confirm the effectiveness of thrip pollination, but we can clearly state that Orobanchaceae flowers are an attractive feeding place for them, both on pollen and tissues.

Ants are ubiquitous, omnivorous, and numerous insects with extensive ecological networks, often visiting flowers and having multiple mutualistic relationships with plants. However, their role as pollinators (myrmecophily) remains poorly researched (Das and Das 2023). It is reasonable to consider the possibility that ants pollinate flowers on purpose and by coincidence while foraging for food. Ants generally forage flowers in search of nectar and other substances, and in doing so, they pollinate the flowers that they encounter. Thus, ant cross-pollination may have led to coevolution between ants and the pollinating flowers (Das and Das 2023). Despite ants’ preference for nectar, which attracts them to flowers, there are cases where they can also destroy flowers, feed excessively on pollen, and repel other pollinators (e.g., Urbani and de Andrade 1997; Cembrowski et al. 2014; Martins et al. 2020). Some studies suggest that ants (*Prenolepis imparis* and *Crematogaster* sp.) can play an important role in pollinating of holoparasitic Orobanchaceae, such as *Epifagus virginiana* (Abbate and Campbell 2013). Another study found that *Salvia absconditiflora* infected by *Orobanche colorata* attracted fewer insects than its uninfected counterparts (likely due to less appealing nectar), and the parasite altered the entomofauna dynamics of the host plant. The nectar of *O. colorata* contains various toxic compounds as a defence mechanism that might repel insects (Özenirler 2024). The author of this observation suggested that while some parasitic plants do not rely on pollinators, they still produce abundant nectar, mainly consumed by ants. However, the appearance of ants may be related to seed dispersal rather than pollination (Özenirler 2024). Our research was not aimed at assessing the effectiveness of ant pollination. However, it showed that ants are very frequent and numerous visitors to Orobanchaceae flowers (Suppl. materials 2, 3, Figs 4, 6). Flowers attract them with deeply hidden nectar, pollen, and succulent tissues. However, other insects, especially aphids that occur not only on the above-ground parts but also underground ones (like haustoria), play a crucial role in attracting ants. We also often observed Orobanchaceae growing shoots in soil arranged as a nesting site for ants. The diversity of ants found is significant, 20 genera were recorded on Orobanchaceae, of which *Lasius* was the most frequently observed, while *Myrmica*, *Tetramorium*, *Crematogaster*, *Formica*, *Plagiolepis*, *Pheidole*, *Camponotus*, *Cataglyphis*, *Dolichoderus*, *Messor*, *Prenolepis*, and *Tapinoma* were less numerous (Suppl. materials 1–3, Fig. 3). Interestingly, the observed ants’ visits were most frequent in the flowers of *Orobanche*, as well as *Cistanche*, *Epifagus*, *Conopholis*, *Hyobanche*, and *Lathraea*, i.e., species colored more brightly, in various shades of yellow, red, white and pink, compared to the blue *Phelipanche*, which ants rarely visited (Suppl. material 3).

We also observed anthophilous flies (Diptera) representing families such as, Anthomyiidae (*Delia platura*), Bombyliidae (*Bombylius cinerascens*, *Hemipenthes maura*, *H. morio*), Chironomidae, Opomyzidae (*Geomyza breviseta*), Phoridae (*Metopina* sp.), and Tachinidae (*Cylindromyia auriceps*) (Suppl. materials 2, 3). Flies, ants or thrips are all small compared to the flowers of Orobanchaceae and probably thieve pollen and nectar, therefore, it is unlikely that they are pollinators; however, when present in large numbers, they may act as an auxiliary to the main pollinators.

#### Snails and slugs

The sweet flower nectar also attracts gastropods. Pollination by snails and slugs (malacophily) is considered the rarest pollination syndrome (Abrol 2011). Pollen can stick to their bodies and be transported to subsequent flowers, and neither snails nor slugs are affected by rainy or windy weather, like other pollinators. Plants pollinated by gastropods are typically characterised by a dense population and non-protruding stigmas and anthers (Pammel and King 1930). Many species of Orobanchaceae possess non-protruding stigmas, which might imply an adaptation for malacophily, although this phenomenon requires further study. During our observations, we noticed various gastropod species inside and on flowers, and less frequently on stems of Orobanchaceae. The most frequently observed snails belonged to the families Helicidae and Geomitridae, notably *Theba pisana* and *Xeropicta derbentina*, which were dominant on different plants across various countries (Suppl. materials 1–3, Fig. 4). These gastropods may be attracted by nectar, pollen, or stigma secretions, as well as seeds (see chapter below). Alternative explanations exist for the snails climbing the stiff inflorescences of these plants. One hypothesis states that snails escape extreme soil surface temperatures by climbing plants (Di Lellis et al. 2012; Schweizer et al. 2019), as holoparasitic Orobanchaceae often prefer dry and hot habitats. However, most of the observations were made in spring, when ground temperatures are not excessive. In such cases, the behaviour may be related to the snails’ circadian activity: they are frequently active at night, especially under warm and humid conditions, while during the day they rest hidden in their shells, which may be stuck to the plant they were feeding on (Cameron 1970; Odendaal et al. 2008).

Snails and slugs may crawl on parasitic plants while seeking moisture. Most of Orobanchaceae species possess trichome-covered surfaces (especially flowers and stems) that aid in water management. Trichomes can capture water from fog and dew through capillary action, supporting plants in drought-prone environments (Li et al. 2023). Additionally, metabolically active glandular trichomes may secrete water derived from host xylem sap, enhancing surface humidity (Světlíková et al. 2015). Such moisture modifications may attract gastropods, influencing their preference for plants possessing these structures.

Such behaviour, especially resting (see also section below) on the stems and inflorescences during the day, is present in some snail species observed on Orobanchaceae outside their native ranges, in areas where these species are regarded as invasive alien gastropods. In this study, *Theba pisana* was observed both in the native Mediterranean region on *Cistanche*, *Orobanche* and *Phelipanche* as well as on *Harveya squamosa* and *Hyobanche sanguinea* in South Africa, where it is an invasive alien species. *Zonitoides arboreus*, native to North America, was also found there on *H. sanguinea*. These snails were first reported in the region at the end of the19^th^ century (Herbert 2010), ruling out any scenario of co-evolution with these plants. Both species are known agricultural pests with generalist feeding habits and are commonly found in coastal South African dune vegetation (van Elden et al. 2015). However, despite their widespread presence, little is known about their ecological interactions with southern African flora, including parasitic plants and their hosts. *Theba pisana* may significantly alter plant community composition through selective herbivory (van Elden et al. 2015). Similarly, *Z. arboreus* is recognised as an adaptable detritivore and fungal grazer, but it may also feed on tender roots and other parts of plants. Furthermore, both species impact ecosystems because they are capable of acting as vectors for parasites and pathogens (Butcher and Grove 2006; Fernandes et al. 2024). Their presence on Orobanchaceae could therefore have direct or indirect effects on both plant health and broader ecosystem interactions, including food web structure (MacDonald and Jarman 1984; van Elden et al. 2015) and pollinator communities (Gess and Gess 2007). In coastal areas where *T. pisana* was absent, the shells of the native endemic snails, e.g. *Trigonephrus*, were abundant, whereas where *T. pisana* was present, indigenous snail shells were rarer. In these habitats, shells serve as refugia for pollinators that built nests inside them. Conversely *T. pisana* shells, though abundant, are too small for many shell-nesters (including several species of the bees and pollen wasps) that depend upon flowers for adult nourishment in the form of nectar as well as pollen and nectar for provision for rearing their larvae. These insects are potential pollinators of the holoparasites or their hosts’ flowers.

However, snails and slugs are herbivorous and sometimes feed on flowers, thereby inhibiting rather than facilitating pollination (Abrol 2011; Suetsugu 2018). Nevertheless, this rare method of pollination cannot be ruled out and requires further research.

#### Mammals and birds

Pollination by non-flying mammals (therophily; primates, rodents, marsupials, and other small mammals) is an uncommon, understudied and fascinating phenomenon. Plants that are pollinated and adapted to rodents have evolved towards specific characteristics, such as usually robust, open flowers placed close to the ground, geoflory, flowers with a large distance between stigma and nectar, with easily accessible sucrose-rich nectar and a characteristic scent, and visually inconspicuous flowers with wide flower entrances, and a musky or musty scent (Wester 2011 and cited references). Surprisingly, in recent years therophily was also discovered in two holoparasitic Orobanchaceae, i.e. *Hyobanche atropurpurea* (dark, almost black geoflorus open flowers, musky or musty smell) and *H. hanekomii* (from light red-purple to very dark, almost black-red flowers, no scent). The species belong to the genus endemic to southern Africa and were pollinated by elephant shrews, such as *Elephantulus edwardii* (Wester 2011; Niedzwetzki-Taubert 2022).

In turn, plants specialised in pollination by birds (ornithophily), which feed on the nectar and thus contribute to pollination, usually have bright coloured flowers (red, yellow or orange), lots of thin liquid nectar, no scent, tubular flowers with long tubes that play a protective role from the potentially destructive behaviour of birds (Turner and Midgley 2016 and cited references; Niedzwetzki-Taubert 2022; Nishimura and Takayama 2022). Interestingly, this pollination syndrome has also been recently discovered in holoparasitic Orobanchaceae, pollinated by birds from Nectariniidae, Pycnonotidae, and Zosteropidae. Recent studies proved pollination of red flowered *Hyobanche sanguinea* in southern Africa by two species of sunbirds (*Nectarinia famosa* and *Anthobaphes violacea*) (Turner and Midgley 2016). Moreover, *Elephantulus edwardii* and also the sunbird *Nectarinia famosa* were observed foraging on *Hyobanche hanekomii* inflorescences, indicating a mixed pollination syndrome or a transition state between bird pollination to pollination by non-flying mammals (Niedzwetzki-Taubert 2022).

It is worth emphasising that, apart from the observations from South Africa in the genus *Hyobanche*, ornithophily has also been demonstrated in Japan, for the first time in the genus *Orobanche*, on pollination of *O. boninsimae*, endemic to the Bonin Islands, by two passerine birds Hypsipetes amaurotis subsp. squamiceps and *Zosterops japonicus* (Nishimura and Takayama 2022). This surprising phenomenon may be related to the oceanic islands, mainly due to their geographical and habitat isolation (mesic forests), where specific evolutionary processes also impact pollinators, woody host plants, and seasonal activities of native insects. Possibly, *O. boninsimae* may be the only bird-pollinated species in the genus *Orobanche*; moreover, this observation supports the hypothesis that a pollinator shift from insects to birds may have occurred in the ancestor of *O. boninsimae* (Nishimura and Takayama 2022), as was observed in some species of *Scrophularia* (Navarro-Pérez et al. 2013). Similarly to Orobanchaceae, most records of bird pollination in orchids are from tropical and subtropical regions (Ackerman et al. 2023). *Harveya* species vary in floral morphology, suggesting different pollinator syndromes (although pollinators have been identified only sporadically). Several species of *Harveya* (*H. bodkini*, *H. bolusii*, *H. scarlatina*, *H. stenosiphon*, and *H. squamosa*; Fig. 2) have morphologies suggesting pollination by birds, having orange to red flowers with long cylindrical corollas borne upright (Randle 2004).

An interesting, albeit solitary, observation of a gecko *Pachydactylus maculatus* (Reptilia, Gekkonidae) sitting on the inflorescence of African *Hyobanche roseoalba*, as well as *Teratoscincus scincus* (Reptilia, Sphaerodactylidae) under a shoot of *Cistanche* in Uzbekistan (Suppl. material 3) may suggest hunting for visiting insects, but there are also reports of lizards and geckos as pollinators from South Africa. Reptilian pollination (saurophily) is one of the rarest and least understood pollination systems, observed mainly on various islands and continentally in South Africa (Wester 2019).

### Seed predation and seed dispersal in Orobanchaceae

Seed predation as granivory (seed feeding) by vertebrates and invertebrates may influence plant breeding and population growth, and may play a role in the evolution of flower and seed traits. Seed dispersal is a fundamental life history trait in plants. It is central to plant reproduction, recruitment, population genetics and ecology because it determines the movement of plant genes in space and time (Lengyel et al. 2010). Seed dispersal modes by animals are usually classified as external (e.g., on fur: exozoochory), or internal (passing through the gut: endozoochory), and by ants (myrmecochory) (van der Pijl 1982).

Holoparasitic Orobanchaceae are characterised by the production of a large number (even several thousand in a single fruit) of one of the smallest types of seeds in the world’s flora, of a length from 0.2 to 1(–5) mm, usually less than 0.5 mm, called ‘dust seeds’ (Piwowarczyk et al. 2020c and cited references; Fig. 2). Only the seeds of *Lathraea clandestina* are unusually large for a parasitic plant, being up to 5 mm, and the capsule contains only a few seeds. Uniquely, the capsules of *L. clandestina*, *L. rhodopea*, and *L. japonica* possess an explosive fruit dispersal mechanism (Atkinson and Atkinson 2020). Germination of the holoparasite seeds depends on their location very close to the root of the preferred host and on the impact of specific hormones – strigolactones extracted by host’s roots (e.g., Yoneyama et al. 2008). Teryokhin (1997) distinguished several ecological Orobanchaceae seed types based on their structure: anemohydrochoric (*Phelipanche*, *Orobanche*, *Phelypaea*, *Aphyllon*, *Boschniakia*, and *Christisonia*), anemochoric (*Cistanche*, *Kopsiopsis*, *Xylanche*, and *Aeginetia*), and zoochoric (*Conopholis*, *Mannagettaea*, and *Phacellanthus*). *Epifagus* (and probably some other genera too) has open and held-up capsules (Suppl. material 2), similar to the well-documented ‘splash cup’ syndrome in other plants, where the minute seeds are ejected with force by incoming raindrops (Thieret 1969; Klaassen van Oorschot et al. 2024), but this requires targeted research. However, some of these proposed adaptations require further studies (Piwowarczyk et al. 2020c), and should consider other modes of seed dispersal or a mix of complex or complementary systems with a greater emphasis on the role of animals (see below).

Apart from larvae of the fly *Phytomyza orobanchia* (Diptera), which is closely associated with holoparasitic Orobanchaceae and also feeds on its young seeds (see above), we found many other examples of seed feeding. During our research, we also observed beetles that can feed on fruits and seeds (Suppl. materials 2, 3, 5: table S5C), but their importance in seed dispersal requires further research, as does the potential role of other arthropods in this process, e.g., ants, lepidopterans, and orthopterans.

Species of carabid beetles are among the most important post-dispersal weed seed predators, removing 65–90% of specific weed seeds shed in arable fields each year (Ali and Willenborg 2021). Many observations of seed eaters, e.g., beetles and moth caterpillars (Suppl. material 3; Manjunath and Nagarkatti 1977; Ray and Dasgupta 2010) require further research into the degree of seed damage and potential seed dispersal. Beetles from the Curculionidae family (larvae and adults), such as *Smicronyx cyaneus*, *Hypera plantaginis*, *H. postica* (Zermane et al. 2001; Suppl. material 3), were found feeding on seeds of Orobanchaceae. Adults of *Gonocephalum depressum* (Coleoptera, Tenebrionidae) congregate on the mature and dry *Aeginetia acaulis* plants and feed selectively on the seeds of the capsule, causing intense damage and seed reduction (Ray and Dasgupta 2010). Moreover, *Bagous*, *Myllocerus*, *Neocleonus* (Curculionidae), *Luperomorpha* (Chrysomelidae), and *Carpophilus* (Nitidulidae) were found feeding on seeds of *Orobanche cumana* in India (Manjunath and Nagarkatti 1977). During our field study, we also observed numerous adult beetles of *Podonta* spp. (Tenebrionidae), *Metadonus anceps*, *Smicronyx fulvipes*, *Sitona macularius* and *S. lineatus* (Curculionidae), as well as *Trichopterapion holosericeum* and *Exapion difficile* (Apionidae) feeding on seeds on faded or dry inflorescences of *Cistanche fissa* and a few *Orobanche* spp. in Poland, Georgia and Azerbaijan (Suppl. materials 2, 3).

The five species of grasshoppers (Orthoptera, Acrididae) that devoured the seed pods of *Orobanche cumana* were fairly common in India (Manjunath and Nagarkatti 1977). The caterpillars of moths were also recorded (Suppl. material 3), but when they occurred, they fed very intensively, especially on the flowers and immature seeds of parasitic plants. Insects, unlike vertebrates, are seldom expected to act as primary seed dispersers via ingestion of fruits and seeds (endozoochory) (de Vega et al. 2011). The seeds of most heterotrophic plants, commonly referred to as dust seeds, are typically dispersed in the air (anemochory) or water (hydrochory). However, a recent study revealed that camel crickets *Diestrammena japonica* and *Tachycines elegantissima* (Orthoptera, Rhaphidophoridae), as well as ground beetles *Synuchus* spp. (Coleoptera, Carabidae) were the major seed dispersers in *Phacellanthus tubiflorus* from Orobanchaceae, and two other non-related achlorophyllous plants. The analysed plants grew in Japan in the understorey of densely vegetated forests, where wind is probably an ineffective seed-dispersal agent. It turned out that the seeds remained intact and viable after passing through the digestive tract of these insects, probably due to the thickened lignified seed tissues. Moreover, its fleshy and indehiscent fruits and the thickened lignified testa of the seeds can be considered as an adaptation for endozoochory (Suetsugu 2018; Piwowarczyk et al. 2020c). It is likely that other frugivorous orthopterans also play a role in seed dispersal, particularly for plants with minute seeds, like holoparasites. However, whether this is a coincidental or more common phenomenon, especially in similar habitats in achlorophyllous plants, requires further research (Suetsugu 2018). We also observed numerous *Phaneroptera* (Orthoptera, Tettigoniidae) visiting on fading, dry inflorescences but still with fresh fruits, e.g. *Orobanche* spp. in temperate zone in Poland (Suppl. materials 2, 3).

In addition, beetle endozoochory has also been documented in achlorophyllous *Cytinus hypocistis* from Cytinaceae (de Vega et al. 2011). Therefore, the life-history traits of these achlorophyllous plants, such as colonisation of dark understorey habitats and dust seeds, will facilitate the independent recruitment of novel endozoochorous seed dispersal systems by insects (Suetsugu 2018). Endozoochory by beetles may facilitate the dispersal of viable seeds after passage through the gut away from the plant to potentially favourable underground sites, offering a high probability of germination success (de Vega et al. 2011). It should be emphasised that seeds of parasitic plants germinate only when they are in very close proximity to the roots of a suitable host and its chemical signals, i.e., strigolactones. The ecological role of endozoochory usually was attributed only to vertebrates. Rodents or other mammals may appear to be better dispersers than beetles because they consume larger amounts of fruit and move seeds on longer distances. However, they leave dung at ground level, not near host plant roots as beetles do, and consume immature fruits, unlike (usually) beetles (de Vega et al. 2011).

Seed dispersal by ants (myrmecochory) is probably the least studied of the main seed dispersal syndromes and also represents a unique opportunity to examine the links between seed dispersal and the evolution of flowering plants. Myrmecochory is mediated by elaiosomes, i.e., lipid-rich seed appendages that attract ants and serve as rewards for dispersal. Elaiosomes provide one of the most dramatic examples of convergent evolution in biology. Myrmecochory is present in at least 11,000 plant species (4.5% of all known species of angiosperms). Most myrmecochorous lineages are Australian, South African and northern temperate (Holarctic) (Lengyel et al. 2010). Some studies suggest that the appearance of ants in *O. colorata* may be related to seed dispersal rather than pollination (Özenirler 2024). We also have observations of the ant *Nylanderia* in ripe open fruits with seeds of *Epifagus virginiana* (Suppl. material 2), but many of the other ant genera we have observed likely also utilise small Orobanchaceae seeds in their diet. It is likely that many of these ants carry the seeds back to their nests (often located under the parasitic plant and its host roots), thereby aiding in effective dispersal. Interesting results demonstrated also that the presence of ants decreases post-dispersal seed predation, even when the ants do not bury the seeds (Tanaka et al. 2015). Some ants of the genera *Pheidole* and *Pogonomyrmex* species are important seed consumers in several desert ecosystems, potentially affecting their abundance in the soil seed bank (Pirk et al. 2009). The seed-carrying behaviour of *Tetramorium tsushimae* was presented in seeds of the common weed species (Ohtsuka et al. 2020), as well as the hemiparasitic plant *Thesium chinense* (Suetsugu 2015). Representatives of these genera, especially *Pheidole koshewnikovi*, *Tetramorium immigrans*, *T. aegeum*, and *T. ferox*, were observed in large numbers in the semi-deserts of Azerbaijan on *Cistanche fissa* flowers, fruits and stems (Suppl. materials 2, 3). We also observed an interesting phenomenon which requires further research, i.e. ants of *Tetramorium* spp. and *Pheidole koshewnikovi* building satellite colonies in the numerous holes and tunnels in the fleshy stems, inflorescences and fruits with seeds, or by bringing lumps of soil and sand into the spaces between the flowers and fruits of *Cistanche fissa* in a few semi-desert localities in Azerbaijan. The tunnels probably result from the flies and beetle larvae simultaneously mining and leaving their excrements, perhaps also ant prey (Suppl. materials 2, 3). However, this requires further research and there may be a link between this phenomenon and the consumption, protection and dispersal of seeds of *Cistanche* by these ant species. We also observed some species of ants in empty, dry stems.

Interestingly, among the myrmecochorous elaiosome-bearing global taxa of plants, only two holoparasitic plants were listed: *Mystropetalum* from Balanophoraceae (Lengyel et al. 2010) and *Lathraea squamaria* from Orobanchaceae (Ortega-Olivencia et al. 2021). Ortega-Olivencia et al. (2021) also included in the database *Cytinus hypocistis* (Cytinaceae), which has no apparent elaiosome but whose fruit pulp envelops the seeds, serving a similar function (de Vega et al. 2011). It seems that *L. squamaria* is the only myrmecochorous elaiosome-bearing species in the holoparasitic Orobanchaceae. Myrmecochorous species exhibit earlier flowering and fruiting than non-myrmecochorous ones in temperate areas (after Ortega-Olivencia et al. 2021 and cited references), such as *Lathraea*, an early spring plant associated with deciduous forests. Myrmecochory is common in temperate forest understory plants for spring-ephemeral and summer-ephemeral life history strategies (Beattie and Culver 1981; Boulay et al. 2007). Plants with elaiosomes also occur sporadically among hemiparasitic species, i.e. *Melampyrum*, *Pedicularis* (Orobanchaceae) or *Thesium* (Santalaceae) (Lengyel et al. 2010; Ortega-Olivencia et al. 2021). Approximately 100 ant species were estimated to be effective seed dispersers (Warren and Giladi 2014). Recent results indicate that in intact forests, ant-myrmecochore mutualisms might tolerate a loss of elaiosome-bearing seeds for a short period (Clark 2022).

In another interesting study, slugs were identified as seed dispersers of seven myrmecochorous species, including *L. squamaria*, in a central European beech forest. An experiment with Arionidae and Helicidae slugs, collected mainly from deciduous forests, revealed that terrestrial gastropods can generally act as seed dispersers of myrmecochorous plants and even substitute myrmecochory, especially where ants are absent or uncommon. Arionid slugs (especially *Arion rufus*, less frequently *A. lusitanicus*) and Helicidae (*Cepaea hortensis*), readily fed on *L. squamaria* seeds, and swallowed seeds were defecated undamaged (elaiosome damage was also not visible) and germinated as well as control seeds in a germination experiment. Surprisingly, gastropods might have been overlooked for decades in the dispersal ecology of myrmecochores (Türke 2011). Interestingly, one of the snails (*C. hortensis*) used in this experiment was also observed in *Orobanche crenata* flowers in Italy (Suppl. material 3). Flowers and shoots of *Lathraea clandestina* are also often grazed by slugs after flowering (Atkinson and Atkinson 2020). Furthermore, the slug *Anadenus yangtzeensis* (Anadenidae) was observed on previous year inflorescences of *Xylanche himalaica* at 3900 m a.s.l., while *Arion subfuscus* (Arionidae) was found on *Boschniakia* (Suppl. material 3).

Mammalian seed dispersal in the holoparasitic Orobanchaceae has also been demonstrated, especially the long-distance dispersal of *Conopholis americana* seeds by deer, which eat the flowering shoots. The viability of seeds after passing through the digestive tract of deer was 48% (Baird and Riopel 1986). The same authors also conducted an experiment under laboratory conditions and observed the consumption of fruits by small mammals from Rodentia (Cricetidae, Sciuridae). In addition to the examples cited, there is also evidence of bears eating inflorescences and stems with tubers, especially of *Boschniakia* and *Conopholis*, and less frequently *Cistanche* (Beeman and Pelton 1980; Eagle and Pelton 1983; Polet and Weber 2008; Mandakh et al. 2020; Suppl. material 3; see also chapter below). However, their influence on effective seed dispersal requires further research. Moreover, seeds may also be dispersed primarily by vertebrates (e.g., passively or ballistically) and secondarily by ants, beetles, etc.

### Carnivores and animals using Orobanchaceae for hunting or shelter

Although spiders are a very numerous and diverse group of arthropods, their role as predators and the cascade effect of their presence on herbivores, pollinators, and plants have not been fully explored. Moreover, only a few studies have reported specific associations between spiders and plants (Vasconcellos-Neto et al. 2017). The architecture of plants influences the abundance and diversity of arthropods, particularly spiders (e.g., Riechert and Gillespie 1986; Vasconcellos-Neto et al. 2017). This applies to various, usually not accidental, vegetative and generative plant structures where spiders can find hiding places, favourable microclimatic conditions, anchoring points for prey-capturing webs, and possibly sites for feeding, hunting or laying eggs (Greenstone 1984; Riechert and Gillespie 1986; Uetz 1991; Dennis et al. 1998; Hesselberg et al. 2023). Some studies showed a high number of spider species inhabiting plants with inflorescences. Inflorescences, especially with open flowers, provide favourable microclimatic conditions and shelters, and can attract different types of prey (Morse 1981). But little is known about a possible mutualism between parasitic plants and spiders. Moreover, most spiders are considered obligatory carnivores; however, in a recent review, Nyffeler et al. (2016) recorded more than 60 species from 20 families of spiders that feed directly on plant products, such as pollen, nectar, stigmatic exudate, plant sap or seeds. Spiders associated with plants have evolved sensory adaptations involving visual, olfactory, and tactile stimuli (review by Romero and Vasconcellos-Neto 2007). Some spiders have also specialised in using plants with glandular hairs that help trap small insects, and the spiders use such carrion to supplement their diet (Romero 2006; Vasconcellos-Neto et al. 2017). During our studies, we often observed small flies, e.g., Sciaridae, Cecidomyiidae (Diptera), or Aphididae (Hemiptera) (Suppl. materials 2, 3) trapped by glandular trichomes of Orobanchaceae, and such easy prey or carrion is most likely used by spiders or other predators.

The impact of spiders on plants can be positive (hunting harmful plant pests) or negative (hunting or repelling pollinators). Insects, including pollinators, can detect flowers that contain ‘sit-and-wait’ spider predators and avoid them due to changes in their behavioural traits. Moreover, spiders and bees are attracted to flowers by scent, and spiders exploit pollinator-attracting signals to find prey (Abrams et al. 1996; Knauer et al. 2018). Plants are locally adapted to attract crab spiders through induced floral volatile emission following herbivore attack (Knauer et al. 2018). Spiders can also negatively affect digestive mutualistic interactions between insects and plants, repel predators of phytophagous insects, and/or consume insects (e.g., ants) that protect the plant against predators (Vasconcellos-Neto et al. 2017).

In our study, the specific architecture of Orobanchaceae (i.e., stiff shoots, usually multiflowered inflorescences, open flowers, trichomes; Fig. 2) and the availability of diverse food sources through the presence of other insect visitors, also turned out to be a convenient habitat for spiders to hunt. The spiders found on Orobanchaceae belonged mainly to the Thomisidae, Araneidae, Theridiidae, and Cheiracanthiidae families; occasionally members of Salticidae, Anyphaenidae, and Dictynidae were also detected (Suppl. materials 2, 3, Fig. 4). Some spiders from the family Thomisidae are typical ‘sit-and-wait’ predators, hunting prey that visit flowers (Morse 1981). We observed various crab-shaped spider species hunting on inflorescences, e.g., *Spiracme striatipes*, *Misumena vatia*, *Xysticus cristatus*, *Thomisus onustus*, *Tmarus piger*. Crab spiders indirectly impact floral-signal evolution by removing florivores (Knauer et al. 2018). Interestingly, spiders from the Thomisidae family are also cited as feeding directly on plant products (Nyffeler et al. 2016). Spiders from the family Araneidae often attach their webs to stiff inflorescence shoots, e.g., *Singa hamata*, *Araneus quadratus*, *A. grossus*, *Neoscona adianta*, *Mangora acalypha* (Suppl. materials 2, 3). We have often observed hunting spiders from the Theridiidae family, especially *Enoplognatha ovata* and *Phylloneta impressa*. An interesting phenomenon was the observation, spread across various countries, of a few species from the mildly venomous genus *Cheiracanthium* (Cheiracanthiidae), which built silken retreats in flowers of three species of *Phelypaea* (red, large single flowers with wide open throat) (Fig. 4, Suppl. material 2). These spiders were also found occasionally on *Orobanche* and *Cistanche* flowers (Suppl. materials 2, 3). Interestingly, those spiders often exhibit cannibalism, with the young feeding on their mother. Thus, perhaps Orobanchaceae flowers provide a good hiding place to protect the young from climate conditions or predators, but this subject requires further research. Moreover, it seems interesting to test the hypothesis of whether such frequent presence of *Cheiracanthium* in flowers, especially *Phelypaea*, caused a repelling of pollinators such as bees in favour of pollinating beetles of the genus *Pygopleurus* (see above). We also have observations of body parts of other arthropod species on holoparasitic plants, such as parts of bees found in flowers (with their heads bitten off), perhaps evidence of hunting spiders. *Cheiracanthium* (and many other spiders) play an important role in the biological control of pests such as the larvae of *Spodoptera littoralis* (Lepidoptera, Noctuidae) (Mansour et al. 1981), and are also reported as predators of lepidopteran eggs (Pfannenstiel 2008) and probably many other moths and arthropods occurring on Orobanchaceae. We also have evidence of underground attraction of spiders, such as cocoons and a female of the also mildly venomous *Steatoda paykulliana* (Theridiidae), at the junction of the haustoria of the parasite and the host-root of two species of *Phelipanche* in the Caucasus (Suppl. materials 2, 3). Furthermore, underground, some spiders, and other arthropods (like ants), may also prey on detritivores (see below) or mining larvae in the tubers and stems of holoparasites. In our opinion, further and more detailed research will reveal many new examples and types of spider-parasitic plant associations.

Many of the recorded arthropods (Suppl. material 3) also use Orobanchaceae for hunting, such as the Miridae or Nabidae (Hemiptera), Syrphidae (Diptera), Formicidae, Coleoptera (e.g., Coccinellidae, Carabidae), Hymenoptera (e.g., Ichneumonidae) or Orthoptera. Social wasps of the family Vespidae may also hunt at flowers of holoparasitic Orobanchaceae on their prey (flies and honeybees or caterpillars) but this was never observed. It is interesting that other plants largely attracting social wasps to their flowers, such as some species of *Epipactis* (Orchidaceae) and *Scrophularia*, emit green-leaf volatiles (GLVs), which are attractive to hunting foragers of the social wasps (Brodmann et al. 2008, 2012). However, this hypothesis fails to explain why flowers of *Epipactis* and *Scrophularia* are visited by no fewer males than females (Veenendaal 2010; Fateryga 2020), even though male wasps do not hunt. In addition, predators such as spiders can also fall prey to spider wasps, e.g. from the family Pompilidae (Finch 1997), whose representatives we noted during the research, e.g., *Arachnospila spissa* (Suppl. material 3), but we did not observe this predation.

Our observations regarding the use of dry flowers and stems of holoparasitic plants as shelter, such as overwintering places, breeding sites, diapause period or satellite colonies for various arthropods, especially ants, flies (e.g., *Phytomyza*, *Polyodaspis*), beetles (Coccinellidae, fungivorous *Propylea quatuordecimpunctata* and *Vibidia duodecimguttata*; some Curculionidae), bugs (e.g., some Miridae, Nabidae, Anthocoridae) and spiders (e.g., Thomisidae, *Tmarus piger*, Araneidae, *Mangora acalypha*, Dictynidae, *Dictyna uncinata*) (Suppl. materials 2, 3), also require further research. We also observed toads (Bufonidae, Bufotes viridis subsp. sitibundus) hiding from the scorching sun under patches of soil raised by growing *Cistanche* in Azerbaijan (Fig. 4). In these hiding places, we also saw various species of hoverfly larvae, spiders, and ants, and it is possible that the toads preyed on them as well.

### Animals using Orobanchaceae as resting sites

Only a few percent of the observations concern resting invertebrates; these included single observations of flies (Diptera) but mainly snails which were observed to stay inactive, hidden in shells glued with dried mucus to plants, during the day because they avoided solar radiation. In our study, 37 records (42% of all snail observations) involved snails resting on the flowers and stems of Orobanchaceae; these observations were made during the day and mostly under sunny weather conditions in open habitats, across 14 countries and three continents (Asia, Europe, and Africa). In total, 14 snail species were observed on 20 Orobanchaceae species belonging to six genera: *Cistanche* (3 records), *Harveya* (1), *Hyobanche* (1), *Lathraea* (1), *Orobanche* (10), and *Phelipanche* (4) (Suppl. materials 2, 3, 5: table S5).

Cook (2001) and Cameron (2016) note that many gastropods exhibit daily and seasonal movement patterns closely linked to the availability of suitable temperature and moisture, with the choice of resting site being a key survival strategy in arid zones. Snails are active only when necessary and are adapted to long periods of inactivity. The snails we observed retreat into their shells on sunny days and attach themselves to elevated structures, such as the flowers and the stems of parasitic plants, whereas they are typically active on cloudy days and warm, humid nights (Fig. 4, Suppl. material 2). This behaviour helps snails avoid harmful ultraviolet radiation (Ali and Said 2019), overheating, water loss and desiccation, as well as predation (Cameron 1970, 2016; Odendaal et al. 2008; Di Lellis et al. 2012; Schweizer et al. 2019). Such responses are typical of xerotolerance and are often accompanied by periods of aestivation. For example, *Theba pisana* is known to climb elevated surfaces, such as plant stems, in hot, dry conditions to reduce exposure to lethal ground temperatures and minimise evaporative water loss (McQuaid et al. 1979; Schweizer et al. 2019).

We also observed sleeping bees, e.g., males of *Eucera* in flowers or clinging under the petals of *Phelypaea coccinea* in Georgia, and males of *Rophites* spp., sleeping in *Phelipanche arenaria* flowers, observed in Poland (Suppl. material 2). Aculeata from the family Chrysididae and Anthophila visited Orobanchaceae only to collect nectar, although males may also use them as a resting site (Michener 2007; Wiśniowski 2015).

### Detritivores

Parasitic plants may also influence soil biota by producing a mineral and nutrient-rich litter, key for maintaining high biodiversity (animals and other plants), especially in low-productivity habitats (Watson 2009). Such litter has a positive effect, especially on detritivores. Hartley et al. (2015) observed a strong positive effect on the abundance of Isopoda and springtails (Arthropleona) as a result of increased density of some hemiparasitic species. Hemiparasites may also impact microbial communities in the soil by decreasing the fungal-to-bacterial ratio and thus increasing litter decomposition rates and nitrogen mineralisation, which influences all trophic levels, with effects evident in herbivores, predators, and detritivores (Bardgett et al. 2006). Future investigation into these relationships is needed, such as holoparasite litter and its impact on soil biota or higher trophic levels (Piwowarczyk et al. 2021b). However, there are some interesting reports on the effects of holoparasite litter on local animal and plant diversity. A survey of developmental sites of biting midges *Culicoides* (Diptera, Ceratopogonidae) in Spain showed that two species (*C. scoticus* and *C. lupicaris*) were strongly associated with soil enriched with leaf (scale) litter occupied by *Lathraea clandestina* (González et al. 2013). Moreover, this species exudes appreciable quantities of liquid, rich in nutrients and minerals, from its scales and this enhances the growth of surrounding vegetation (Atkinson and Atkinson 2020). After flowering of *L. clandestina*, springtails (Collembola) are common among the remains of flowers and shoots (Atkinson and Atkinson 2020). We also observed detritivores at the underground decaying tubers of holoparasitic plants, especially Collembola, larvae of various Diptera flies from Syrphidae, Chloropidae, Agromyzidae, Sciaridae, Cecidomyiidae or Sarcophagidae (especially *Eumerus*, *Polyodaspis*, *Phytomyza*, *Sarcophaga*, *Dicraeus*, *Lestodiplosis*, *Bradysia*), beetles (e.g., weevil larvae of Curculionidae and Scarabaeidae, with adult Scarabaeidae, e.g., *Pentodon*, Geotrupidae like *Phelotrupes*, Tenebrionidae like *Opatroides*, *Pedinus*), larvae of Lepidoptera (e.g., *Anarta sabulorum* from Noctuidae), Isopoda (Agnaridae, *Hemilepistus klugii* and *Protracheoniscus verhoeffi*), mites (Anoetidae) (which can simultaneously decompose plant tissue along with the excrements of fly and beetle larvae contained within it), and earthworms (Lumbricidae) (Suppl. materials 2, 3, Fig. 3). At the same time, faded, dried flowers, as well as seeds, are a food source for detritivores; for example, we observed Coleoptera (e.g., *Ptinus meisteri* from the Ptinidae, Bruchinae and Alticinae from the Chrysomelidae, or *Cortinicara gibbosa*, *Corticaria obscura*, *Corticarina truncatella*, *Melanophthalma distinguenda* from Latridiidae), Blattodea (*Ectobius*), Thysanoptera, or Polyxenidae (*Propolyxenus argentifer*) (Suppl. materials 2, 3, Fig. 4). The species listed above which were found in dry flowers, may also be granivorous. Simultaneously, predators were observed utilising the detritivore community, especially spiders, beetles, hemipteran, ants, various parasitoids or vertebrates. However, detritivores are probably much more numerous and diverse, and interconnected by various food webs and require further targeted study after the plants have decayed above and below the ground.

### Orobanchaceae as hosts of various stages of insect development

Adult stages of animals dominate in many genera (average 90.9%), indicating that these plants may often attract mature insects for various activities such as nectar collection and pollination. It appears that some parasitic plants may also support the development of larval stages, which consume parts of the plants (Suppl. material 5: table S5), which also serve as a breeding ground. We observed numerous eggs on the stems and flowers of Orobanchaceae, especially laid by Hemiptera, but also rarely by Diptera and Lepidoptera (Suppl. materials 2, 3, 5: table S5). Thus, these insects use Orobanchaceae as a breeding site and perhaps also the host plant. Interesting observations include the aphid *Dysaphis aff. middletoni* on the inflorescence of *Epifagus virginiana*. In particular, the presence of autumn oviparous females (oviparae) laying eggs indicates that this is a monoecious species (although it can be polygynous), i.e. feeding all year round and overwintering on this plant. We also observed various juvenile stages of other groups of insects, e.g. Coleoptera (larvae of Curculionidae in underground tubers, larvae of *Meloe proscarabaeus* (Meloidae) in flowers); Diptera (the above-mentioned larvae and eggs of *Phytomyza orobanchia* (Agromyzidae), larvae of *Eumerus* (Syrphidae), larvae of *Sphaerophoria* (Syrphidae), larvae of *Polyodaspis*, eggs of *Tabanus* sp. (Tabanidae)); Hemiptera (different juvenile stages of various species of the Aphididae family, *Aphrodes bicincta* nymph (Cicadellidae), *Adelphocoris lineolatus* larvae (Miridae)), larvae, nymph of mites (Anoetidae), and numerous observations of larvae from Thysanoptera. Interestingly, we found different developmental stages (eggs, larvae, and adults) of *Dolycoris baccarum* (Pentatomidae) on various holoparasitic plants (Suppl. materials 2, 3). This indicates that this bug is particularly common in these plants. It should be remembered that for aphids, the plant is the only place of feeding, so the plant feeds aphids in all developmental stages and in the aphid colony the larvae usually dominate in numbers, unless there is a change to another host plant; then all (or most) of the aphids reach adulthood and fly away.

We also recorded juvenile individuals of Lepidoptera (caterpillars from the families Geometridae, Noctuidae, Crambidae, Erebidae, Pterophoridae, Tortricidae, and Zygaenidae), and Orthoptera (e.g., Gryllidae, Tettigoniidae), as well as juvenile Araneae (Suppl. materials 2, 3). Tenthredinidae and Cephidae (Hymenoptera from the suborder Symphyta) lay eggs in plant tissues so that the larvae can feed on or in the plants (Gaulet and Huber 1993; Gauld and Bolton 1996), and adults may feed also on nectar. We also have observations of an exuvia of spiders, and rarely cicadas on the inflorescence of *Aphyllon pinorum* from North America and *Orobanche hederae* and *O. laxissima* from Georgia (Suppl. material 3), from which adult cicadas (Cicadidae, e.g. *Tibicina steveni*) emerged, and in this case, the holoparasitic plants served as the site of metamorphosis.

### Other multitrophic interactions, mutualism and antagonism between animals inhabiting Orobanchaceae

Animal species recorded on Orobanchaceae are also subject to many mutual interactions, especially the mechanisms of co-evolution in plant-pollinator/phytophage-parasitoid-predator-protector systems. The first good example, often observed, is the mutualistic relationship between ants and aphids. In exchange for their sweet excretion, honeydew (trophobiosis), phytophagous aphids receive careful care and protection from ants from various natural predators (Delabie 2001). Numerous ant species, especially *Lasius*, are linked with various species of aphids through a symbiosis. This relationship covers aphids found on the above-ground parts of Orobanchaceae shoots and those living on their underground parts, i.e. tubers and haustoria, i.e. *Smynthurodes betae* with *Lasius flavus*, *Rectinasus buxtoni* with *Pheidole koshewnikovi* (Suppl. materials 2, 3). Aside from ants’ floral nectar-gathering behaviour, they also indirectly provide an anti-herbivory defence strategy since ants, while patrolling, often attack many species of predatory arthropods and thus protect the plants (Das and Das 2023). Ant-plant interactions can be incorporated into a multi-network species interaction in a multi-trophic environment, where ants may fulfil multiple roles, such as pollinators, seed dispersers, and protectors (Del-Claro et al. 2018; Das and Das 2023).

The fly *Phytomyza orobanchia*, so common in Orobanchaceae, is often attacked by Braconid and Chalcidoid parasitoids (Aphelinidae, Braconidae, Eulophidae, Eupelmidae, Pteromalidae) (Černý et al. 2022; Suppl. material 3).

Some species of hoverflies in the family Syrphidae have carnivorous larvae that feed on aphids, lepidopteran larvae, and other soft-bodied herbivores (Li and Wu 2022; Li et al. 2023). During our research, we recorded aphidophagous larvae of the genus *Sphaerophoria* (Suppl. materials 2, 3). We found numerous *Meloe* (Coleoptera, Meloidae) larvae on the flowers of *Orobanche alsatica* (Suppl. material 3). These larvae (triungulin) are dangerous to bees because they attach to the body of a female visiting the flower and when they reach the nest, they migrate from the bees’ body and devour the eggs, the brood, and the pollen loafs in the provisioned nest cells (Topolska et al. 2001). Spiders are often involved in complex food webs or direct or indirect interactions with other arthropods and plants. However, studies showing evidence of mutualism between plants and spiders are scarce (Vasconcellos-Neto et al. 2017). Spiders, although they have a positive impact by protecting plants from phytophagous insects, can also have strong negative effect on pollinators; predated pollinators will avoid plants with spiders, and that eventually will affect plant fitness and plant-pollinator mutualism (Dukas 2001; Dukas and Morse 2003; Heiling and Herberstein 2004).

We also found predatory mites (various species associated with bumblebees, other bees and beetles), which are probably a bit more common in Orobanchaceae flowers, waiting there for their hosts, but due to their small size they are overlooked. We managed to observe *Parasitellus* sp. (Mesostigmata, Parasitidae) on flowers of *O. centaurina* in Romania (these mites are phoretic on *Bombus* spp.; Schwarz and Huck (1997)), and another mite (Parasitidae, subfamily Pergamasinae) inside *O. alsatica* flowers in Poland (Suppl. materials 2, 3), as well as a report of a predatory mite (Mesostigmata, Gamasina) on *Lathraea squamaria* flowers in Ukraine (Krasylenko et al. 2024). In addition, we observed mites (Parasitidae, Anoetidae) parasitising the bodies of beetles (Tenebrionidae) feeding on flowers and fruits, as well as on larvae of flies from tubers of *Cistanche fissa* in Azerbaijan (Suppl. materials 2, 3). The larvae of many flies are hemipteran parasitoids, e.g. *Cylindromyia auriceps* (Diptera, Tachinidae) parasite on *Dolycoris baccarum* (Hemiptera, Pentatomidae) (Suppl. material 3). Several flies, e.g. *Sarcophaga* spp. have been recorded as parasitoids of terrestrial snails (Coupland and Barker 2004). Adult parasitic wasps (Hymenoptera) feed mainly on nectar, but as parasitoids (except Cynipidae), their females may search on Orobanchaceae for hosts (insects and spiders) for their larvae. The recorded Braconidae might search for larvae of Lepidoptera (e.g., Pyralidae), Diptera (Alysiinae–Diptera from the suborder Cyclorrhapha), and Coleoptera (Gaulet and Huber 1993; Gauld and Bolton 1996; Ghimire and Philips 2014). Cynipidae from the genus *Phanacis* form galls in the stems and sometimes roots of various species of herbaceous plants and shrubs (Asteraceae, Lamiaceae, Papaveraceae, Rosaceae) (Nieves-Aldrey et al. 2008), including potential parasitic plants’ host species, but it has not been observed in Orobanchaceae. Females of observed Ichneumonidae may seek lepidopteran larvae (including Drepanidae, Geometridae, Lasiocampidae, Noctuidae, Nolidae, Nymphalidae, and Sphingidae) as hosts for larvae (Kolarov et al. 2024). Platygastridae may seek eggs and larvae of Diptera from the family Cecidomyiidae and Coleoptera from the families Curculionidae and Cerambycidae, as well as various groups of Hemiptera (e.g., Coccoidea, Aleyrodoidea, and Auchenorrhyncha–Fulgoromorpha and Cicadomorpha) for larvae (Gaulet and Huber 1993; Buhl and Jałoszyński 2016). The recorded species from the family Pteromalidae is a parasitoid of Cecidomyiidae eggs or spiders (Olfert et al. 2020). Females of the recorded representatives of Scelionidae (*Trissolcus nigripedius*) may search for eggs of Pentatomidae to parasitise, such as *Dolycoris baccarum* (which is often recorded on Orobanchaceae in all developmental stages) (Suppl. material 3), and also attack other heteropteran pests (Mahmoud and Lim 2008; Indihar et al. 2024). A myrid on *Orobanche cumana* was parasitised by a braconid and prayed upon by a reduviid (Manjunath and Nagarkatti 1977). The recorded Spheciformes from the family Crabronidae, apart from nectar, may search for prey for their offspring on Orobanchaceae in the form of small Auchenorrhyncha from the families Cercopidae, Cicadellidae, Cixiidae, Dictyopharidae, Flatidae, and Issidae (Polidori et al. 2007), and small Diptera (mainly Anthomyiidae, Muscidae, Syrphidae) (Skibińska 1986; Woydak 1996). The observed Scoliidae searched for nectar, and females of *Arachnospila spissa* and cf. *Auplopus carbonarius* (Pompilidae), apart from nectar, may have also searched for spiders from the families Salticidae and Lycosidae for example, which are food for their larvae (Wiśniowski 2009).

### Arthropod–plant transmission as potential vectors of beneficial and pathogenic microbiome

Microorganisms are important associates of arthropod and plant species. Insect-associated microbes, including bacteria, fungi, and viruses, can drastically impact host physiology and ecology. However, many microbes still have an unknown role. Insect resident gut microbiota can also detoxify plant defensive compounds (Holt et al. 2024). Symbiotic microbiomes act as important regulators of the diverse lifestyles of insect hosts and participate in the three-fold interactions of plants, insects, and natural enemies. These various microorganisms can be found in whole insects, and exoskeletons, intestines, saliva, etc. (Zhao et al. 2022; Serdo and Degaga 2023; Chang et al. 2024). Insects visiting and using plants can exchange their microorganisms through the interaction between co-occurring insects and the plant itself.

Hemipteran insects, especially aphids (numerous on Orobanchaceae), are also considered the most important viral and phytoplasma vectors. Although there is no experimental evidence of parasitic plants mediating phytopathogens from arthropods to other plants, this potential transmission route requires more studies (Savov et al. 2024). Besides, it seems that the arthropod-parasitic plant-host plant route of phytopathogen transfer is not significant, but some arthropods are specific to their parasitic plants as obligate nutritional symbioses, e.g., the fly *Phytomyza orobanchia* (Savov et al. 2024), *Eumerus* spp., and other potential new species discovered during our research, thus this subject requires further observations (Suppl. materials 2, 3, Fig. 3). Recent works uncovered that stigmas in flowers in holoparasitic Orobanchaceae plants host diverse bacterial and fungal communities, including pathogens of insects (Ruraż et al. 2023a, 2023b; Wiśniewska et al. 2025, 2026). *Beauveria bassiana* was isolated from mature stigmas in flowers of *Phelipanche arenaria* (Ruraż et al. 2023a). This entomopathogenic fungus is a parasite on various arthropod species, causing white muscardine disease, and is used as a biological insecticide to control numerous pests, including thrips, whiteflies, aphids, beetles, and moths (Sánchez-Rodríguez et al. 2018). Plant-associated microbes contribute to induced systemic plant resistance by enhancing chemical and morphological defences, but on the other hand, endosymbionts of insects also help their hosts to overcome plant defences by detoxifying plant metabolites (Sharma G. et al. 2021). Some bacteria found on Orobanchaceae flower stigmas (Ruraż et al. 2023a, 2023b; Wiśniewska et al. 2025, 2026) can also emit microbial VOCs that attract parasitoid wasps (Cusumano et al. 2023). Plants, for example, can respond to aphid herbivory by altering their volatile organic compound profile to attract the aphid’s natural enemies, which can ultimately cascade to entire insect populations and associated communities (Frago et al. 2012). Moreover, recent studies have also shown a high diversity and functional traits of the endophytic bacterial communities in the seeds of a few holoparasitic Orobanchaceae (Petrosyan et al. 2022, 2023, 2026). Thus, microorganisms can be exchanged, modified and transferred between flower/fruit-visiting insects, most often anthophilous ones, herbivorous pests, its parasitoids, and possibly their carnivorous enemies. Complicated and complex trophic systems, encompassing parasitic plants, their hosts, insects, and microorganisms, demand further investigation.

### Holoparasitic Orobanchaceae as a food resource for vertebrates and humans

It appears that holoparasitic plants are, in whole or in part, also a very important or additional source of food in the diet of mammals, reptiles, birds (nectarivorous), and humans. The extent of this phenomenon is poorly understood and requires further research and exploration, but there are several important reports. The genus *Conopholis*, also called squawroot or bear corn, is a common food eaten by black and brown bears, e.g., in the USA and Mexico (Beeman and Pelton 1980; Eagle and Pelton 1983). *Conopholis* is probably an important energy source for bears in spring in the southern Appalachians because the carbohydrates in squawroot are readily absorbed (Eagle and Pelton 1983). The combination of high-protein and relatively carbohydrate-rich herbaceous material makes *Conopholis* an important resource for bears recovering from the denning period and particularly for lactating females (Eagle and Pelton 1983). Although *Conopholis* is not an abundant species, the plant makes up even 10–15% of bears’ diet in the Smoky and Shenandoah Mountains. Data indicate that bears eat whole shoots, but some observations indicate that they prefer the fruits and thus contribute to seed dispersal (Suppl. material 3). There are also reports of *Conopholis americana* being eaten by deer in natural habitats and by rodents (Cricetidae, Sciuridae) in laboratory experiments (Baird and Riopel 1986).

Moreover, there are also confirmed reports of the use of *Boschniakia rossica* (mainly its tuber) as food by brown and black bears, especially in Alaska (e.g., Polet and Weber 2008; Suppl. material 3). Some evidence has been shown that brown bears in Mongolia consume *Cistanche deserticola* (Mandakh et al. 2020), and gazelles eat *C. tubulosa* in the United Arab Emirates (Hornby 2003). There are also observations of sheep, goats and cows grazing in fields infected with *Phelipanche aegyptiaca* in Israel (Basheer et al. 2024). Also rare, but interesting, are the observations of tortoises eating flowers of two species of the genus *Hyobanche* in South Africa (Turner and Midgley 2016; Niedzwetzki-Taubert 2022) and young inflorescence tops of *Cistanche* in Azerbaijan (Suppl. material 3). In *Orobanche boninsimae*, most flower visitors were observed after sunset, and black rats were among them, feeding on stems and floral organs (Nishimura and Takayama 2022). What compounds exactly the animals complement and what effect eating holoparasitic Orobanchaceae has on them remains to be elucidated by further research. However, recent studies have shown that polysaccharides from *Cistanche deserticola* can be used to regulate rumen flora and fermentation in grazing sheep to improve immunity and production efficiency (Zhang et al. 2022).

Holoparasitic Orobanchaceae have been used for thousands of years in traditional medicine, especially in China and America, as an herb or functional supplement (e.g., Shi et al. 2020). However, in our work, we wanted to focus only on the typical use as food for humans. Unfortunately, this knowledge is scarce and fragmented. The earliest known information on *Orobanche crenata* as a food can be found in Pliny the Elder’s *Book of Natural History* from AD 77–79 (after Bianco et al. 2009). The priest Kluk (1787) lists *O. elatior* (but he could likely have confused it with another species of *Orobanche* s.l.) in Poland as an edible plant that can be eaten like asparagus, and Łuczaj (2004) also added that other domestic species may probably be edible. There is also information about the consumption of various species of *Orobanche* (now *Aphyllon*) by Native Americans, who ate raw, roasted or boiled lower and underground parts of the shoots (Łuczaj 2004). *Aeginetia indica* is used as a food colourant, and the whole plant is cooked with sugar and nutmeg and eaten as an antiscorbutic in traditional Thai desserts (Useful Tropical Plants 2019). In recent years, there has been a growing interest in healthy food, and information and research are beginning to emerge showing that holoparasitic Orobanchaceae (especially those occurring abundantly as weeds or intentionally cultivated) can become an excellent source of food for humans.

*Cistanche* (the whole plant, especially when young) is widely consumed in many countries, especially in China, as a health food, with its dietary supplements also being widely available and in increasing demand globally (Zhu et al. 2025). The most commonly used are *C. deserticola*, endangered in many regions, often obtained from the wild illegally, therefore listed in the Appendix II of the Convention on International Trade in Endangered Species of Wild Fauna and Flora (CITES). A good solution was to introduce this species as a new cultivated plant in some regions of China, which now covers an area ca. 84,000 ha, with an annual output of 6000 tons (Song et al. 2021). However, there is potential to expand cultivation of *Cistanche* beyond China (Thorogood et al. 2021), especially due to the growing demand for this plant and its protection in wild. Therefore, our research on phytophagous and pollinating fauna can also be helpful in cultivation of holoparasitic plants. In some areas of Puglia in Italy, *O. crenata* is consumed similarly to asparagus and is widely used for several traditional dishes. It is considered a food with interesting nutritional traits (i.e., high fibre content, good antioxidant capacity and presence of polyphenols). In 2015, this holoparasite was listed in the ‘List of Traditional Agri-Food Products of Puglia’, and local farmers harvest and sell it under the local name “sporchia” (Renna et al. 2015). Recent studies also showed that another invasive crop weed, *Phelipanche aegyptiaca*, analysed in Israel, has potential as a novel food source for humans and animals, where there are observations that cattle have also been eating it (Basheer et al. 2024). Interestingly, literature data on traditional uses showed that they were mainly used for medicine in China and North America, while in Europe, *Orobanche* s.l. were used primarily as food (Shi et al. 2020). The consumption of holoparasitic Orobanchaceae by humans and vertebrate animals is still poorly understood. It requires further field studies and interviews, but there may also be local differences, depending on the geographical region. In Europe, on xerothermic grasslands or in subalpine grasslands, which are often also pastures for cattle, sporadic consumption by these animals has been observed (Piwowarczyk, pers. observ.). However, the situation may change dramatically in poor habitats with sparse vegetation, such as semi-deserts and deserts, or forests in early spring, when the ground cover is still poor (see examples above). In such cases, holoparasitic plants rich in valuable sap may constitute a very important food source also for vertebrates.

### Evolution of Orobanchaceae holoparasitic lineages in relation to animals and global factors

Coevolution between plants and animals mainly occurs between plants and herbivorous and pollinating or seed-dispersal insects, mainly due to various chemical and physical mechanisms (Labandeira 2013; Bruce 2015; Serdo and Degaga 2023). Beyond the mutualistic interactions, most are allospecific, involving species or sets of species, often utterly unrelated in dynamic systems (Bascompte and Jordano 2013). Holoparasitism is also associated with the high specificity of the particular host plants and impacts organisms’ biology and ecology (including pollination, herbivory, and speciation).

Parasitism has evolved independently more than 12 times from their free-living ancestors, while holoparasitism has evolved in nine families across angiosperm phylogeny (Nickrent 2020). Orobanchaceae is believed to have a mid-Tertiary Laurasian origin (Wolfe et al. 2005). Divergence dating reveals that the three origins of Orobanchaceae holoparasitism were not synchronous. Moreover, holoparasitism may have evolved independently as an adaptation to certain host plants (Fu et al. 2017; Fig. 1). The oldest holoparasitic clade is Orobancheae (14 genera, ca. 244 species), where all representatives are non-photosynthetic. The second clade is Buchnereae (including four holoparasitic genera *Hyobanche*, *Harveya*, *Aeginetia*, *Christisonia* (67 species)), and the remaining hemiparasitic). The youngest clade, Rhinantheae, comprises only five species from the single genus *Lathraea* (Fu et al. 2017, modified), the only holoparasitic genus in this clade (the remaining being hemiparasitic). Divergence time estimates showed that the transition to holoparasitism in the *Lathraea* lineage occurred relatively recently, whereas the holoparasitic lineage Orobancheae is about two times older (Samigullin et al. 2016). The extensive diversity of the tribe Orobancheae, the oldest and the most species-rich lineage of holoparasitic Orobanchaceae, is concentrated mainly in Western Asia and Mediterranean regions of the Old World (McNeal et al. 2013; Schneider and Moore 2017; Piwowarczyk et al. 2021a). Recent investigations have proved that Western Asia (particularly the Caucasus) and the Mediterranean are the centres of origin for large clades of holoparasitic Orobancheae within the last 6 million years (Piwowarczyk et al. 2021a). In the Caucasus, the centres of diversity are composed both of old and recently diversified clades, while in the Mediterranean there appear to be representatives mostly of recent diversification (Piwowarczyk et al. 2021a). In the Caucasus, about 30 species of Orobancheae are known to be endemic (15 confirmed species), or to have most of their range restricted to this region (Piwowarczyk et al. 2021a).

The oldest evolutionary clade Orobancheae, characterised by the highest diversity and repeated occurrence of key insect families, is pollinated mainly by diverse arthropods, especially Hymenoptera, particularly by bees and wasps, rarely syrphids, beetles or moths, with only one exception on an oceanic island where birds pollinate an endemic species. This observation supports the hypothesis that a pollinator shift in the ancestor of *O. boninsimae* from insects to birds may have occurred (Nishimura and Takayama 2022). Holoparasites represented by the intermediate and very morphologically diverse clade Buchnereae (tropical Asia, eastern and southern Africa, especially Cape Floristic Province) are characterised by high heterogeneity and large contribution of non-insect animals. It has a mixed pollination syndrome or a transition state between pollination by mammals and birds, as well as long-tongued hawkmoths and other arthropods, like bees, but the data are incomplete, and the genera are still poorly known. The evolution of the sizeable floral diversity of the Cape Floristic Kingdom of South Africa may be a result of the heterogeneous and mosaic nature of the habitats, allowing for large-scale diversification on a small geographical scale. In contrast, the youngest clade Rhinantheae with one holoparasitic genus *Lathraea* is pollinated almost exclusively by bumblebees (Suppl. materials 2, 3, Fig. 6). Overall, the three phylogenetic clades differed markedly in both the taxonomic breadth and dominance structure of their associated animal visitors. The evolution of pollination may also have been non-synchronous. It may have been linked to the evolution of host selection, which further determined the habitat, seasonality, and thus the content of insects vs. mammals or birds and further adaptive changes. Shifts between pollinator niches have been a key driver of adaptive radiation in angiosperms (e.g., van der Niet and Johnson 2012).

Hymenoptera is the most frequent visitor group of Orobanchaceae across most continents, emphasising its global ecological importance for evolution of holoparasitic plants. Among these, Apidae and Formicidae were the most consistently represented families across plant clades. Interestingly, in North America, the secondary groups were mammals (Carnivora and Rodentia). Unique dominance patterns, such as Stylommatophora or pollinators such as birds and mammals in subtropical or tropical regions, especially Africa, may reflect regional ecological adaptations and the role of local fauna in shaping plant-animal interactions. Some continents, like Asia and Africa, show greater diversity in the visiting groups (Fig. 4). These patterns suggest that holoparasitic plants may have evolved region-specific interaction strategies shaped by continental differences in faunal composition and environmental conditions. Unfortunately, many regions and plants require further observations, as some are more thoroughly studied based on multiple targeted observations (e.g., up to 80 different animal species can benefit from one holoparasitic species), while many plants have single or accidental observations (Suppl. material 5: table S4), and almost 60% of species have no data (Suppl. material 1).

Overall, the results demonstrate that parasitic plants attract a functionally and structurally diverse fauna, with genus-specific differences in ecological roles, developmental stages, and plant parts visited. Flowers were the central mediators of interactions, but in some genera, interactions extend to stems, tubers, and other vegetative structures (Figs 2, 4, Suppl. material 2). Only 5% of the animal observations were made on the underground parts of the plants, but our findings suggest greater potential for future research. While adult insects dominate the visitors, larval and juvenile stages contribute to ecological complexity (Suppl. material 3, 5: table S5). The variety and distribution of ten identified ecological roles that fauna fulfils within holoparasitic plant genera reveal a complex network of interactions specific to each plant genus. It appears that some parasitic plants may largely attract phytophagous fauna (average ca. 40%) that consume parts of their structure. Anthophilous species dominate in many genera (average 28.3%), indicating that these parasitic plants often rely on animal visitations for nectar or pollen collection (Suppl. material 5: table S5). These findings underscore the ecological versatility of parasitic plants, reflecting their ability to support diverse faunal communities across multiple functional levels. In our work, we tried to relate these multi-specific systems to entire networks, infer their influence on ecological and evolutionary processes, and understand how diversified mutualisms among animals and plants evolve and coevolve into megadiverse networks of species. Understanding complex networks of interaction will help assess patterns of evolution in generalisation and specialisation, as well as temporal and spatial changes in their main components.

Rich data support the theory that pollinator-mediated selection accounts for divergence in flower size and shape among geographically isolated populations (Galen 1999 and cited references). Natural selection may favour generalisation in floral adaptations that affect pollination efficiency rather than specialisation on a single species of pollinator or alternative pollinating agents. Convergence in floral preference implies that similar floral traits will be favoured in populations or among plants serviced by different groups of animals (Galen 1999; Johnson and Raguso 2016). However, new evidence suggests that selection on flower form and size is a more pluralistic process, involving not only pollinators but also enemies, like herbivores, and even some aspects of the plant’s abiotic environment. Thus, ecological forces other than pollinators may drive the evolution of flower morphology (Galen 1999).

Pollinator diversity often provides an incomplete explanation for the evolutionary divergence of flower morphology, which may have not only attractive functions, but also defensive roles, against nectar or pollen thieves and robbers, flower herbivores or ovule predators. Therefore, conflicting selection pressures related to floral attractiveness and defence may maintain variation in the shape and size of flowers and floral organs, emphasising the importance of the cross-talk between both types of interactions for plant evolution (Galen 1999; Ramos and Schiestl 2019). For almost all animal-pollinated flowering plants, complex, multispecies interactions with floral enemies, such as herbivores, often result in conflicting selection of plant traits (Jacobsen and Raguso 2018). A broad understanding of these multiple ecological factors should extend beyond the role of pollinators and include herbivores, especially florivores, as a major component of the ecology of angiosperms and a major driver of their evolution (Boaventura et al. 2022).

The examples of diverse herbivory, especially florivory and granivory, described above may also have been one of the selective forces in the evolution of these plants, and it would be interesting to investigate this in more detail in the future. Interestingly, myrmecophily occurs only in the youngest clade (Rhinantheae) in the holoparasitic *Lathraea squamaria*, and in the hemiparasitic members of this clade (*Melampyrum* and *Pedicularis*). However, whether ants’ dispersal system influenced the evolution of these plants requires further investigation. The dust seeds of holoparasitic plants are typically dispersed in the air or water. Additionally, and uniquely in Orobanchaceae, three of the five *Lathraea* species have an explosive fruit dispersal mechanism (Atkinson and Atkinson 2020). However, a recent study revealed that in the understorey of densely vegetated forests, where wind may be an ineffective seed dispersal agent, orthopterans and beetles are the major seed dispersers of *Phacellanthus tubiflorus*, which acquired adaptations for endozoochory (Suetsugu 2018). Perhaps a similar seed dispersal system will be found among other holoparasitic genera of Orobanchaceae occurring in similar habitat types, especially forests. Holoparasitic Orobanchaceae are also exclusive food and development sites for many herbivorous insects, e.g., the flies *Phytomyza orobanchia* and *Eumerus* spp., and many other animals connected by multilevel interactions. Holoparasitic plants are obligately dependent on host species and may also change their host ancestor during evolution, which entails changes in the habitat and, thus, in the surrounding animals, both pollinators and herbivores. Agricultural environments are often simplified, with less habitat diversity than natural ecosystems (Bruce 2015). Thus, other selection processes will pressure invasive holoparasitic plants parasitising cultivated plants in crops on almost homogeneous sites with relatively poor local biodiversity. However, some weeds contribute to biodiversity in agroecosystems and support the delivery of regulating ecosystem services by increasing the number of beneficial arthropods involved in pollination and biological control. However, they may also contribute negatively by increasing the number of pests in crops, and by functioning as a source of diseases for insects and crop plants (review by Serdo and Degaga 2023).

The above-mentioned relationships also overlap with climate change, which may affect holoparasites directly by impacting their physiology and indirectly by impacting their host plants and habitats (Piwowarczyk and Kolanowska 2023a, 2023b). Most anthropogenic stressors associated with global change have been shown to affect trophic webs in multiple ways (Tylianakis and Morris 2017), usually with net negative effects on species, interactions, and a simplification of network structure (Tylianakis et al. 2008, 2010). Biotic interactions and its robustness are key species- and community-level responses to global change (McCauley et al. 2012; Pocock et al. 2012; Tylianakis and Morris 2017). Failure to protect such interactions may result in many species going extinct before they are recorded, and before we learn the role they play in their ecosystems (Heleno et al. 2020).

Pollinator populations around the world have been declining at an alarming rate in recent decades, and ca. 40% of invertebrate pollinators, such as bees, wasps and butterflies, and 16% of vertebrate pollinators, especially birds, are at risk of extinction (IPBES 2016). Bees are the most important pollinators, besides a small and rare group of the pollen wasps, the only group that actively gathers pollen while foraging (Brunet and Fragoso 2024). These pollinators most actively pollinate species from the clade Orobancheae. A recent study suggests that the projected increased severity of heatwaves may expose bumblebee-mediated pollination services by disrupting the chemical communication between plants and pollinators (Nooten et al. 2024). Bumblebees are particularly vulnerable to ongoing climate warming because they are well-adapted to cold environments (Kerr et al. 2015). In our study, we showed that bumblebees play a significant role in the pollination of various species and genera, especially in the Northern Hemisphere, in the clade Orobancheae, and almost exclusively in *Lathraea* in the clade Rhinantheae (Suppl. material 3, Fig. 6).

The plant-herbivore interactions have evolved in response to coevolutionary dynamics, along with selection driven by abiotic conditions. The paleontological records documented increased herbivory during periods of global warming in the deep past. Climate change factors can increase herbivore consumption rates, probably leading to greater foliar damage to annual plants and floral damage to perennial plants. Moreover, some insect herbivores may shift from one to multiple generations per year under climate change. The immediate effects of the climate change on plant and herbivore functional traits could impose strong selection and alter long-term evolutionary dynamics (review by Hamann et al. 2021).

It is likely that more chemically diverse plants share more chemicals with other plant species, which could facilitate host switches and increase herbivore richness. Herbivore similarity may decrease gradually at increasing taxonomic levels of their host plants (comparisons among host genera, families, etc.). The degree of similarity also differs among guilds, depending on their host specialisation, and should be lower for specialists than for generalists. Beta-diversity is a key component in understanding the spatial organisation of such assemblages (Lewinsohn et al. 2005). Perhaps also important is the fact that in most cases the host plants do not flower simultaneously with the parasites, so they do not share their niches, at least partially. A similar pattern concerns seed dispersal or florivory.

## Conclusions

We found data on fauna in over 40% of all species and 84% of the genera in the holoparasitic Orobanchaceae worldwide. In total, a wide diversity of animals was represented by 34 orders, 163 families, 434 genera, and 667 species, with arthropods (key role of hymenopterans) overwhelmingly dominant. Our fieldwork, supplemented by the compilation of available data on animals associated with holoparasitic plants, gathered substantial evidence on pollinator- and herbivore-driven evolution, unravelling the origins of the astonishing morphological and taxonomic diversity of these plants and explaining their macro-evolutionary dynamics. The results show that parasitic plants attract a functionally and structurally diverse fauna, with mainly clade-genus-specific differences in ecological roles, developmental stages, and plant parts visited, with significant geographical and habitat effects. One of the most striking results of our analysis is the spatial scale over which we may find divergent coevolutionary trajectories.

Flowers of the holoparasitic Orobanchaceae have evolved a plethora of strategies to attract various pollinators, and many adaptations to cross-pollination, as well as developing several unique defence mechanisms from insect herbivores: intrinsic (chemical or mechanical), and extrinsic (including the protection of predators and parasitoids). The diversity of flowers is primarily shaped by selection and evolutionary change caused by the plant-animal interactions which are a major driver of plant evolution, including both local adaptation and species divergence. We have demonstrated direct mutualistic (such as pollinators and seed dispersers) and antagonistic relationships (such as different herbivores), but also many poorly understood indirect interactions with animals from the third trophic level (e.g., predators, parasitoids, and detritivores). The relationships we have identified are often very complex; one animal can perform several functions, as a pollinator, herbivore, predator, parasitoid or protector, and this depends on the developmental stage of the animal and plant, as well as the ecosystem’s and other biotic and abiotic features. The results presented here shed light on the potential for supergeneralist interactions, including both mutualisms and antagonisms, in shaping typical evolutionary trajectories between animals and angiosperms as exemplified by a unique group of holoparasites.

Our research not only demonstrates the very high biodiversity of animals utilising the holoparasitic Orobanchaceae but also highlights that all species are part of complex networks of interactions and trophically connected cascades. Recognition of these interactions is essential for protection, through understanding the consequences of species extinctions and the functioning of entire ecosystems. We also identified a number of critical gaps in our knowledge of animal-holoparasitic plant interactions, which may prove vital for identifying ecological linkages required for preserving biodiversity. These data may inform syntheses for ecological and evolutionary studies and more practical applications in refining local-to-global conservation strategies. Our work opens and indicates new windows for further research and can also serve as a guide for anyone wishing to pursue research into plant-animal associations.

## Additional information

### Artificial Intelligence (AI) use

The authors accept full responsibility for the content of the manuscript, including the disclosure of any use of AI.

No AI tools were used in the preparation of this manuscript.

### Author ORCIDs

R. Piwowarczyk https://orcid.org/0000-0003-0507-7835

K. Wiśniewska https://orcid.org/0000-0003-4831-7712

T. Rewicz https://orcid.org/0000-0002-2085-4973

T. Olbrycht https://orcid.org/0000-0003-2854-4467

S. Salata https://orcid.org/0000-0003-0811-2309

A.V. Fateryga https://orcid.org/0000-0002-5346-3477

Ł. Nicewicz https://orcid.org/0000-0003-3998-2673

Ł. Depa https://orcid.org/0000-0003-4204-5568

K. Zając https://orcid.org/0000-0003-2359-9258

R. Masarovič https://orcid.org/0000-0002-9523-1711

W. Celary https://orcid.org/0000-0001-6395-2680

Ł. Mielczarek https://orcid.org/0000-0002-7553-4619

A. Mátis https://orcid.org/0009-0007-6853-8308

### Data availability

All of the data that support the findings of this study are available in the main text or Supplementary Information.

## Supplementary materials

Supplementary material 1 Supplementary statistical analysis Data type pdf https://binary.pensoft.net/file/1666426This dataset is made available under the Open Database License (http://opendatacommons.org/licenses/odbl/1.0/). The Open Database License (ODbL) is a license agreement intended to allow users to freely share, modify, and use this Dataset while maintaining this same freedom for others, provided that the original source and author(s) are credited. Renata Piwowarczyk, Karolina Wiśniewska, Tomasz Rewicz, Tomasz Olbrycht, Sebastian Salata, Alexander V. Fateryga, Łukasz Nicewicz, Łukasz Depa, Katarzyna Zając, Rudolf Masarovič, Waldemar Celary, Łukasz Mielczarek, Attila Mátis

Supplementary material 2 Supplementary photographic boards of orders of recorded fauna Data type pdf https://binary.pensoft.net/file/1666427This dataset is made available under the Open Database License (http://opendatacommons.org/licenses/odbl/1.0/). The Open Database License (ODbL) is a license agreement intended to allow users to freely share, modify, and use this Dataset while maintaining this same freedom for others, provided that the original source and author(s) are credited. Renata Piwowarczyk, Karolina Wiśniewska, Tomasz Rewicz, Tomasz Olbrycht, Sebastian Salata, Alexander V. Fateryga, Łukasz Nicewicz, Łukasz Depa, Katarzyna Zając, Rudolf Masarovič, Waldemar Celary, Łukasz Mielczarek, Attila Mátis

Supplementary material 3 Database of animals recorded on holoparasitic Orobanchaceae Data type xlsx https://binary.pensoft.net/file/1666428This dataset is made available under the Open Database License (http://opendatacommons.org/licenses/odbl/1.0/). The Open Database License (ODbL) is a license agreement intended to allow users to freely share, modify, and use this Dataset while maintaining this same freedom for others, provided that the original source and author(s) are credited. Renata Piwowarczyk, Karolina Wiśniewska, Tomasz Rewicz, Tomasz Olbrycht, Sebastian Salata, Alexander V. Fateryga, Łukasz Nicewicz, Łukasz Depa, Katarzyna Zając, Rudolf Masarovič, Waldemar Celary, Łukasz Mielczarek, Attila Mátis

Supplementary material 4 Molecular database of animals’ collection with GenBank accession numbers Data type xlsx https://binary.pensoft.net/file/1666429This dataset is made available under the Open Database License (http://opendatacommons.org/licenses/odbl/1.0/). The Open Database License (ODbL) is a license agreement intended to allow users to freely share, modify, and use this Dataset while maintaining this same freedom for others, provided that the original source and author(s) are credited. Renata Piwowarczyk, Karolina Wiśniewska, Tomasz Rewicz, Tomasz Olbrycht, Sebastian Salata, Alexander V. Fateryga, Łukasz Nicewicz, Łukasz Depa, Katarzyna Zając, Rudolf Masarovič, Waldemar Celary, Łukasz Mielczarek, Attila Mátis

Supplementary material 5 Additional tables Data type pdf Explanation note **table S3**. Biodiversity metrics of identified fauna (orders) inhabiting parasitic plant genera. **table S4**. Taxonomic composition of fauna associated with parasitic plant genera, expressed as the proportion (%) of observations for each taxonomic group. **table S5**. Proportional distribution (%) of observations of fauna associated with parasitic plant genera, showing (A) visited plant parts, (B) developmental stages of fauna, and (C) ecological roles of fauna. https://binary.pensoft.net/file/1666430This dataset is made available under the Open Database License (http://opendatacommons.org/licenses/odbl/1.0/). The Open Database License (ODbL) is a license agreement intended to allow users to freely share, modify, and use this Dataset while maintaining this same freedom for others, provided that the original source and author(s) are credited. Renata Piwowarczyk, Karolina Wiśniewska, Tomasz Rewicz, Tomasz Olbrycht, Sebastian Salata, Alexander V. Fateryga, Łukasz Nicewicz, Łukasz Depa, Katarzyna Zając, Rudolf Masarovič, Waldemar Celary, Łukasz Mielczarek, Attila Mátis

Supplementary material 6 Additional tables Data type xlsx Explanation note **table S6**. Biodiversity metrics of identified animal taxa recorded from individual parasitic plant taxa. **table S7**. Taxonomic composition of animal taxa associated with individual parasitic plant taxa, expressed as the percentage (%) of total observations. https://binary.pensoft.net/file/1666431This dataset is made available under the Open Database License (http://opendatacommons.org/licenses/odbl/1.0/). The Open Database License (ODbL) is a license agreement intended to allow users to freely share, modify, and use this Dataset while maintaining this same freedom for others, provided that the original source and author(s) are credited. Renata Piwowarczyk, Karolina Wiśniewska, Tomasz Rewicz, Tomasz Olbrycht, Sebastian Salata, Alexander V. Fateryga, Łukasz Nicewicz, Łukasz Depa, Katarzyna Zając, Rudolf Masarovič, Waldemar Celary, Łukasz Mielczarek, Attila Mátis
