# Supplementary material for: A multiverse of trophic networks and coevolutionary trajectories among holoparasitic Orobanchaceae and their animal associates: a global perspective
Source: PhytoKeys. 2026 Jun 2;275:209–97. doi: 10.3897/phytokeys.275.192014 (PMC13250618; doi:10.3897/phytokeys.275.192014)
Supplement: Supplementary material 1 — Supplementary statistical analysis [file phytokeys-275-209_article-192014__-s001.pdf]

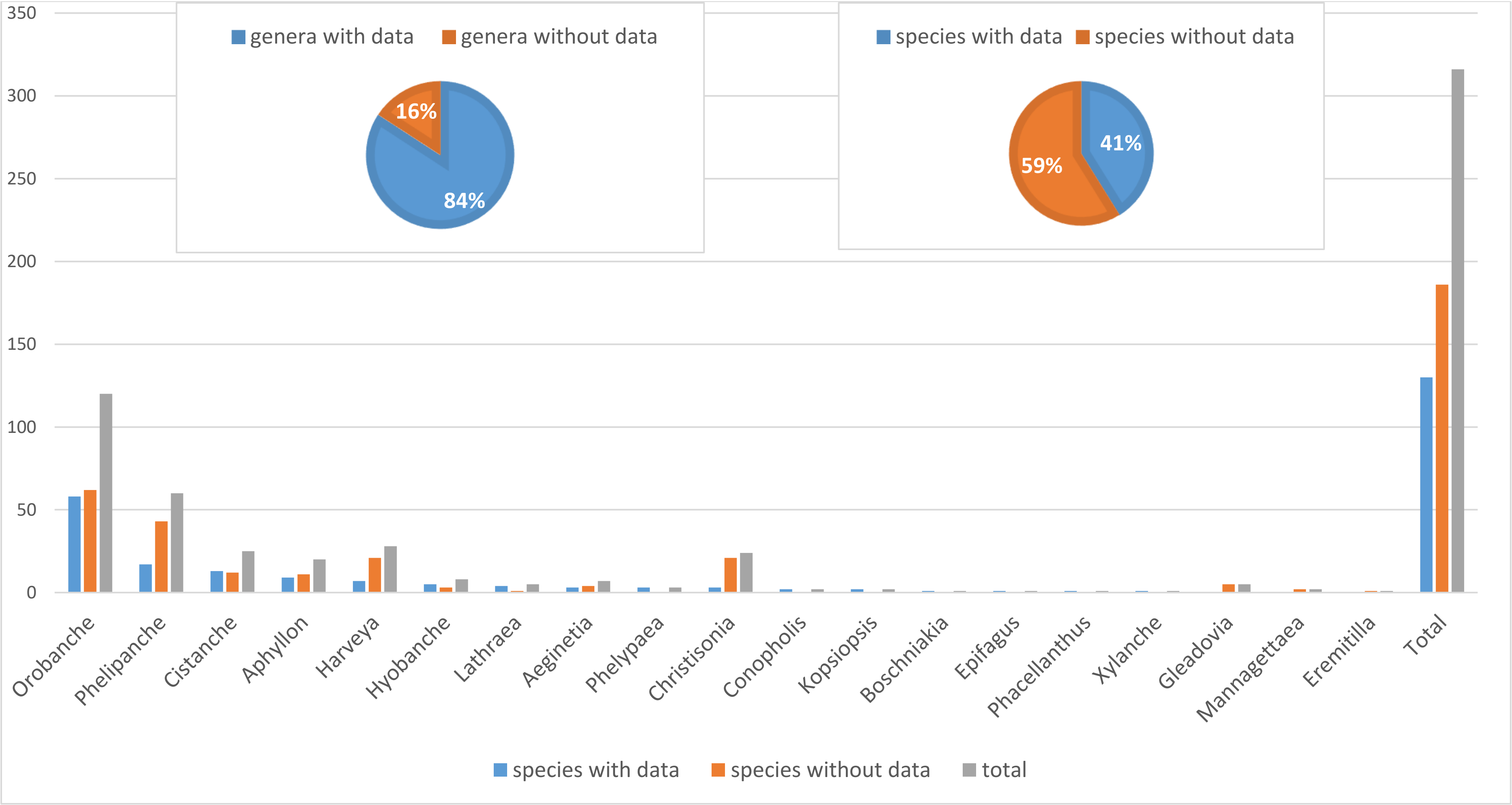

Figure S1.1. Species in genera of holoparasitic Orobanchaceae with or without data on animals with comparison of its total diversity.

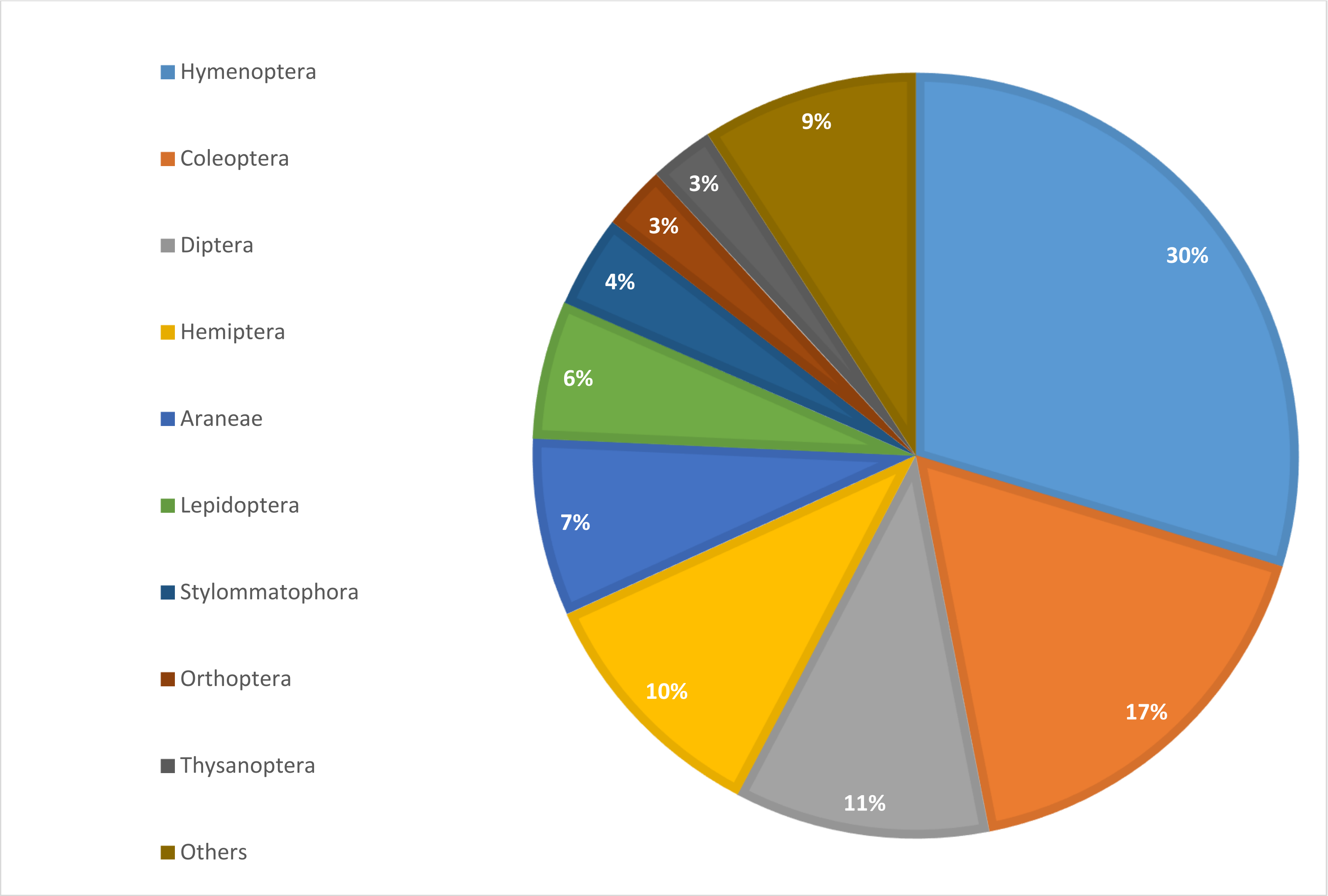

Figure S1.2. Percentage of species of the largest orders of animals.

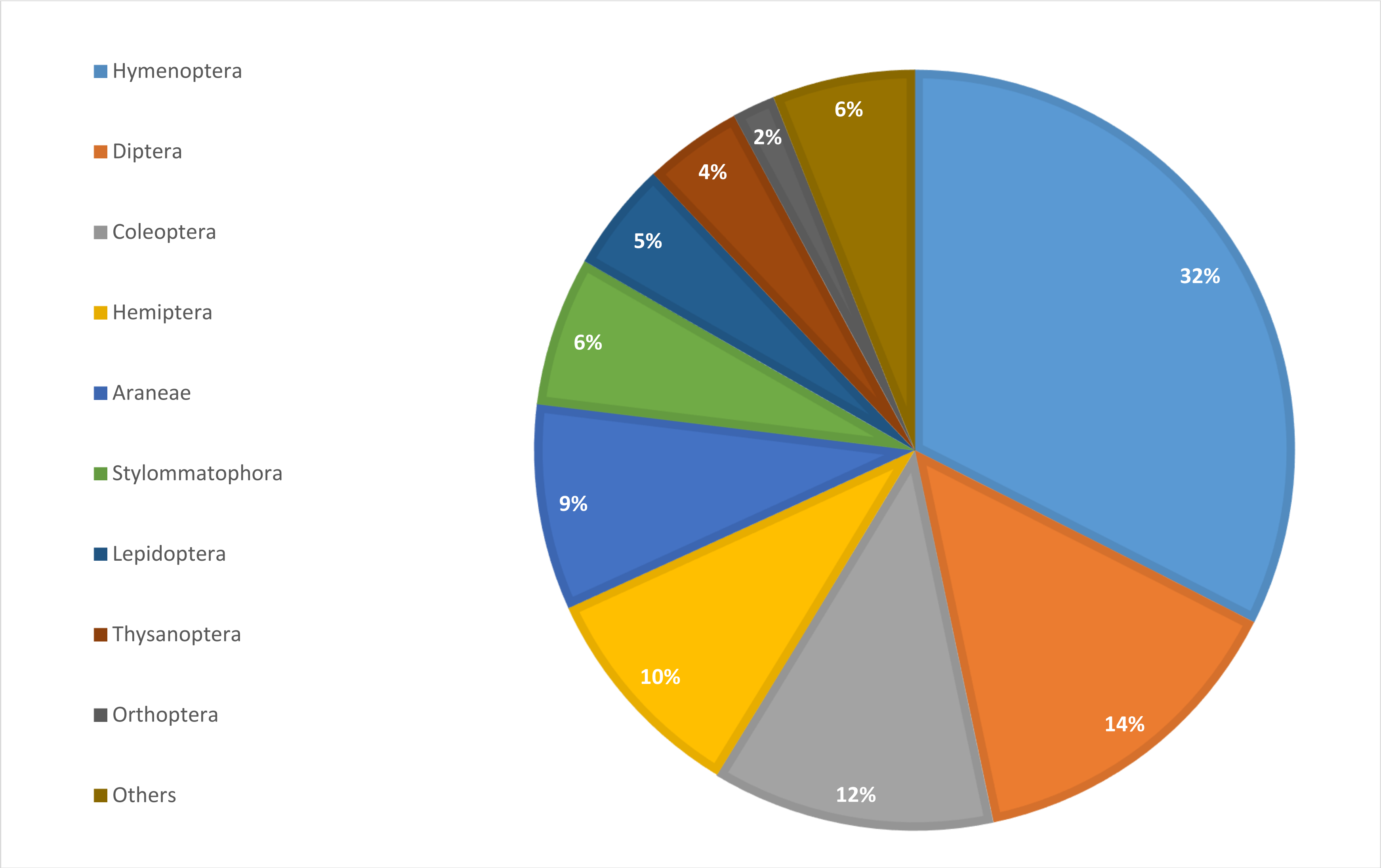

Figure S1.3. Percentage of observations of the largest orders of animals.

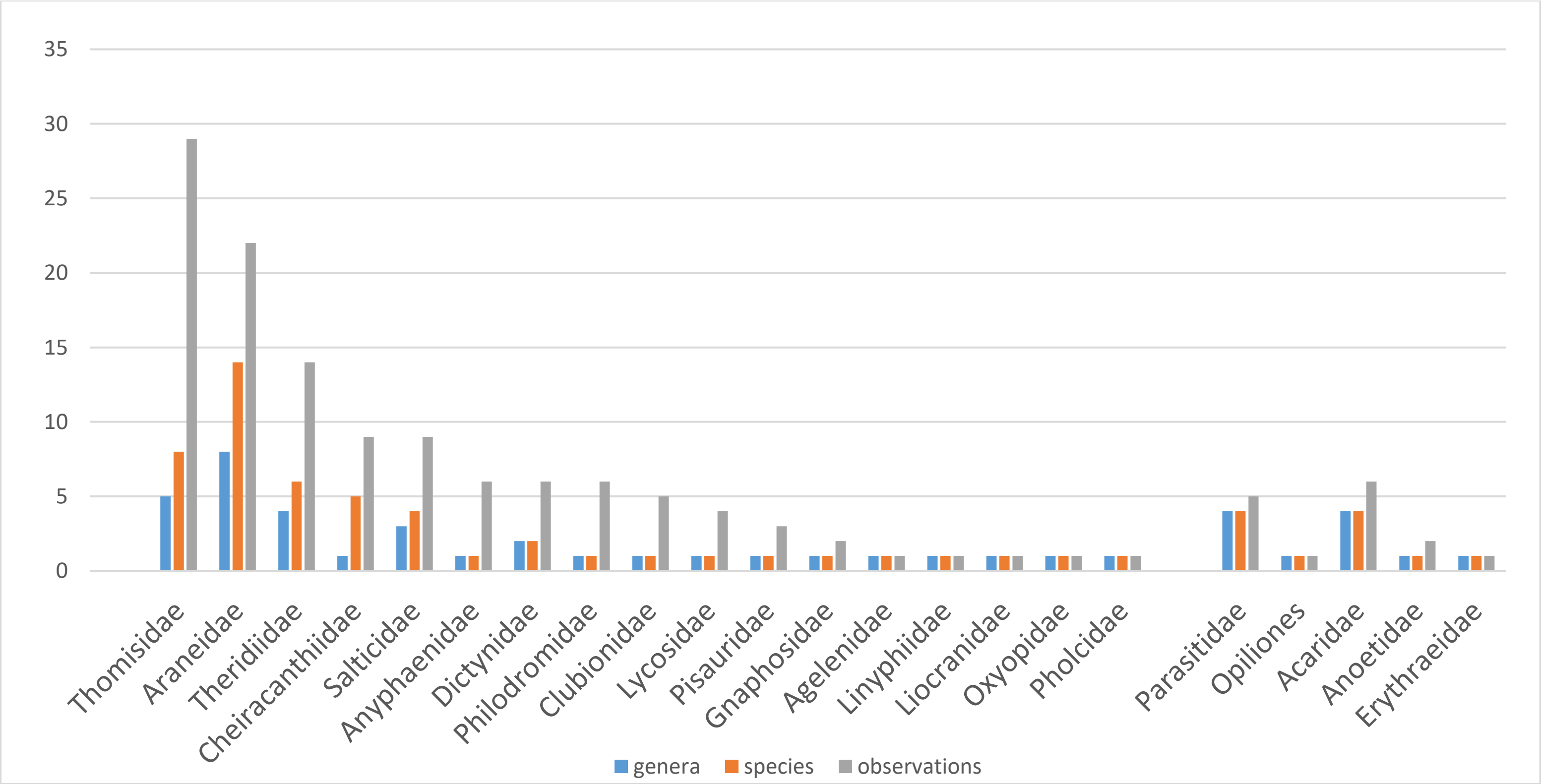

Figure S1.4. Number of genera, species and observations of families in Araneae (left) and other Arachnida (right).

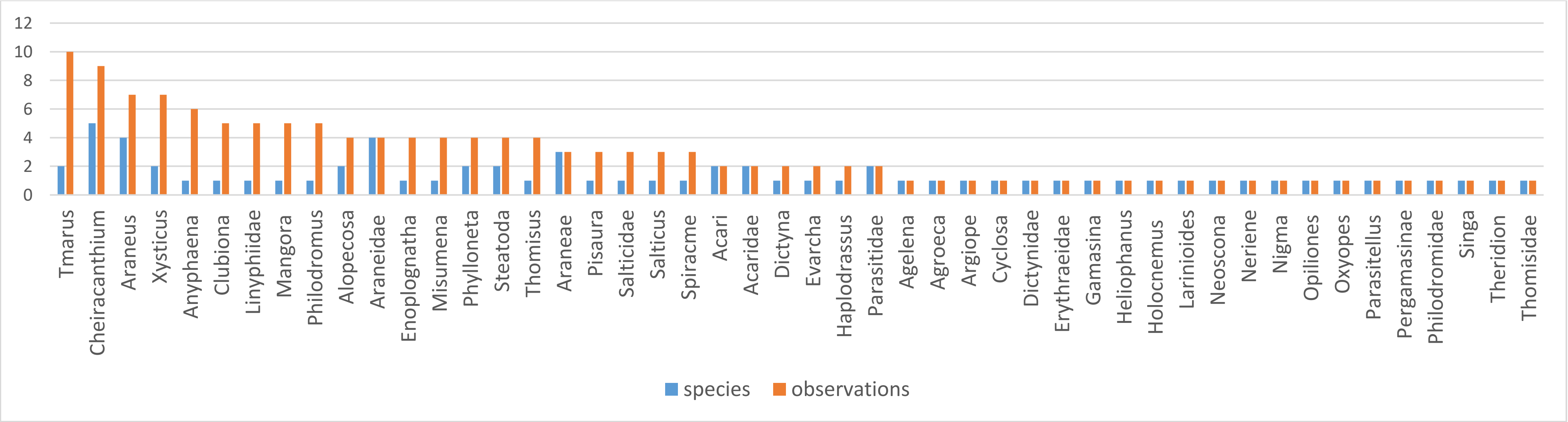

Figure S1.5. Number of species and observations in genera of Arachnida.

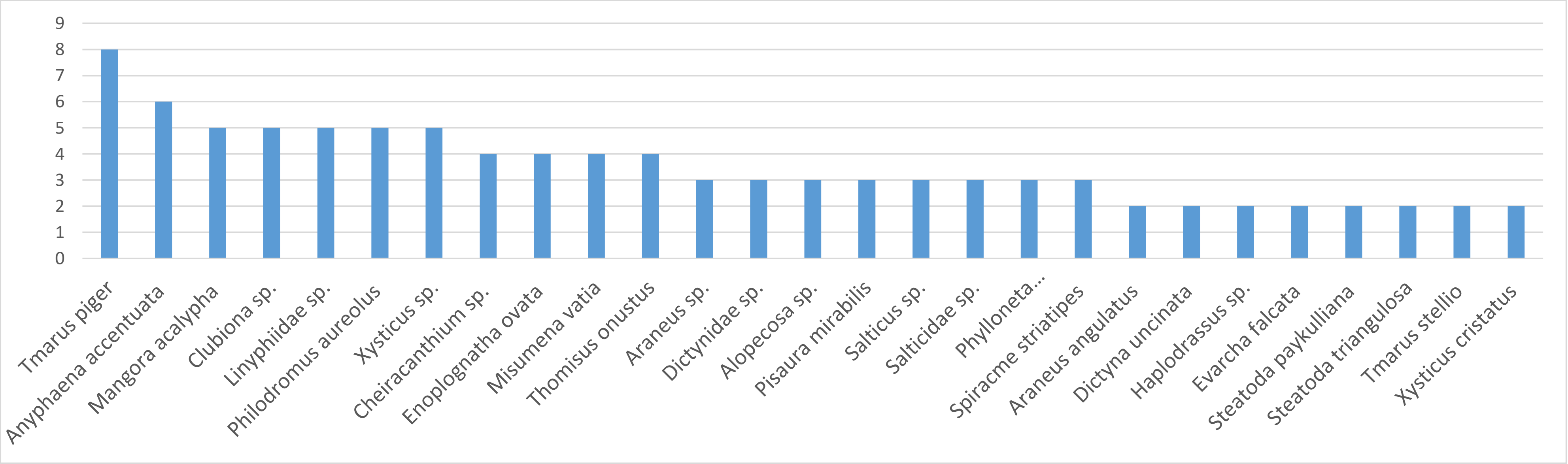

Figure S1.6. The most numerous observations (from 2 to 8) of species in Arachnida.

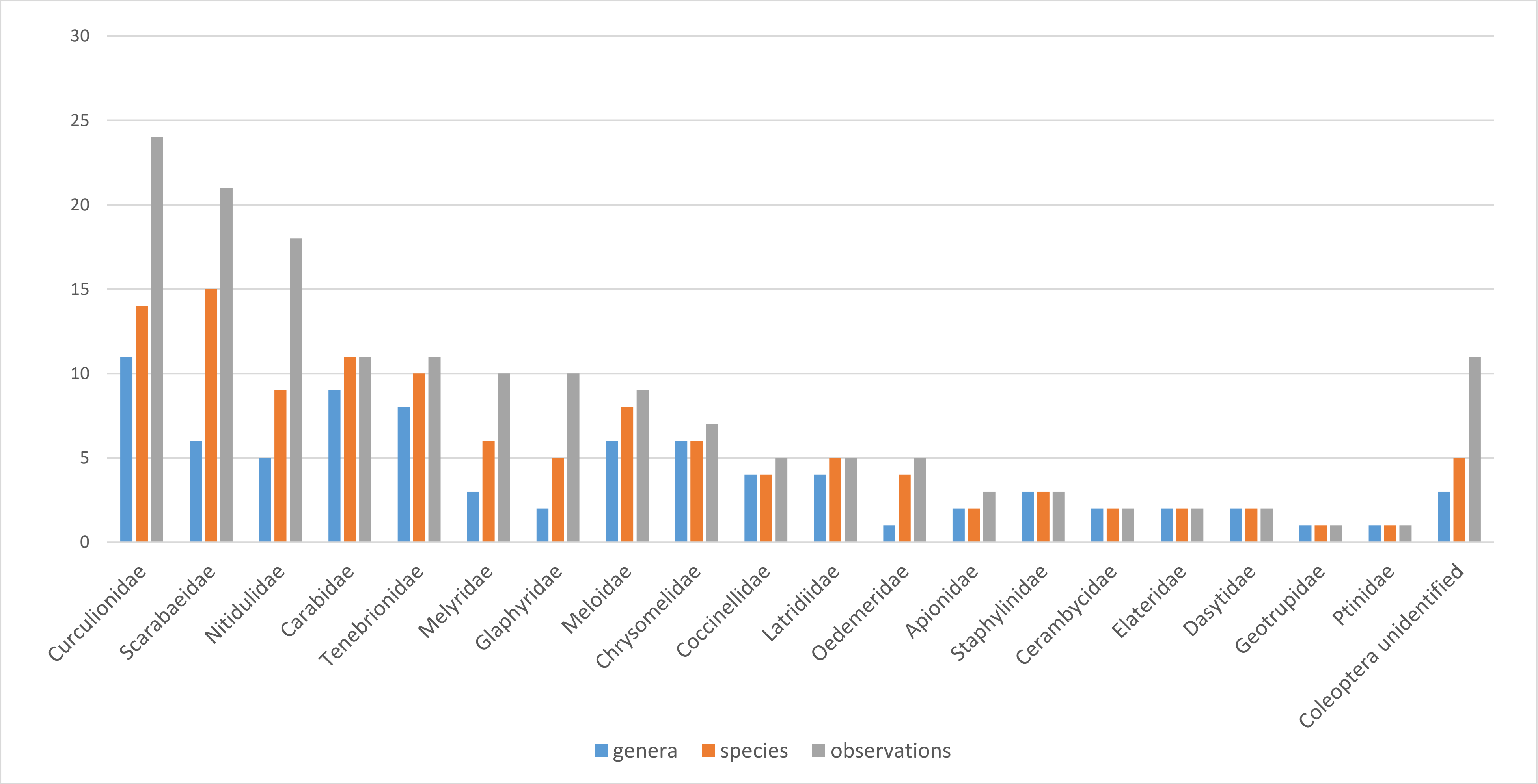

Figure S1.7. Number of genera, species and observations in families of Coleoptera.

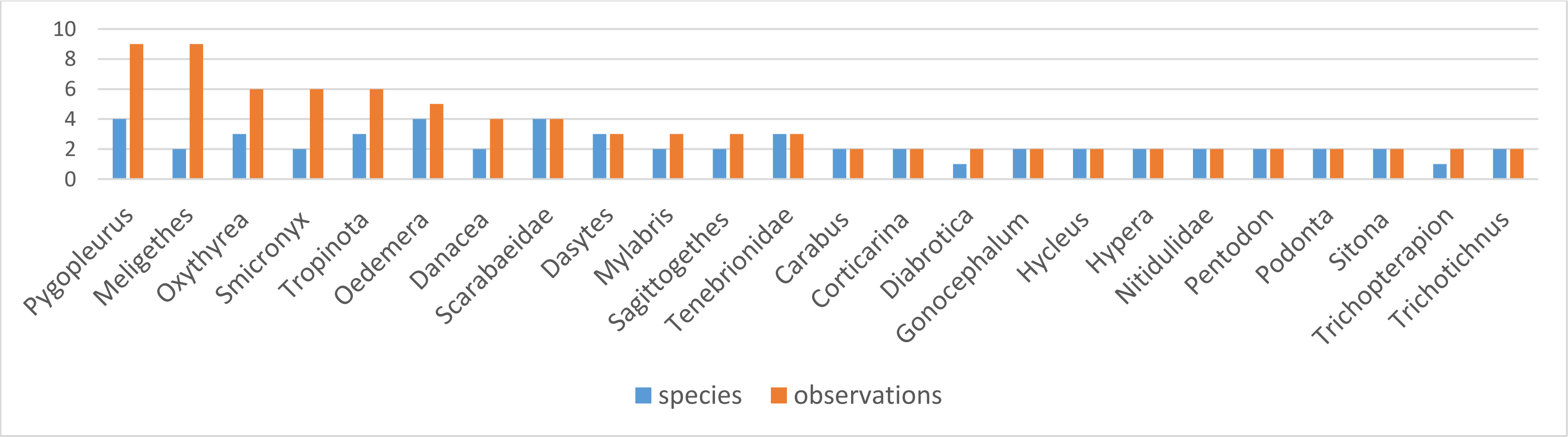

Figure S1.8. Number of species and observations (from 2 to 9) in the most numerous genera of Coleoptera.

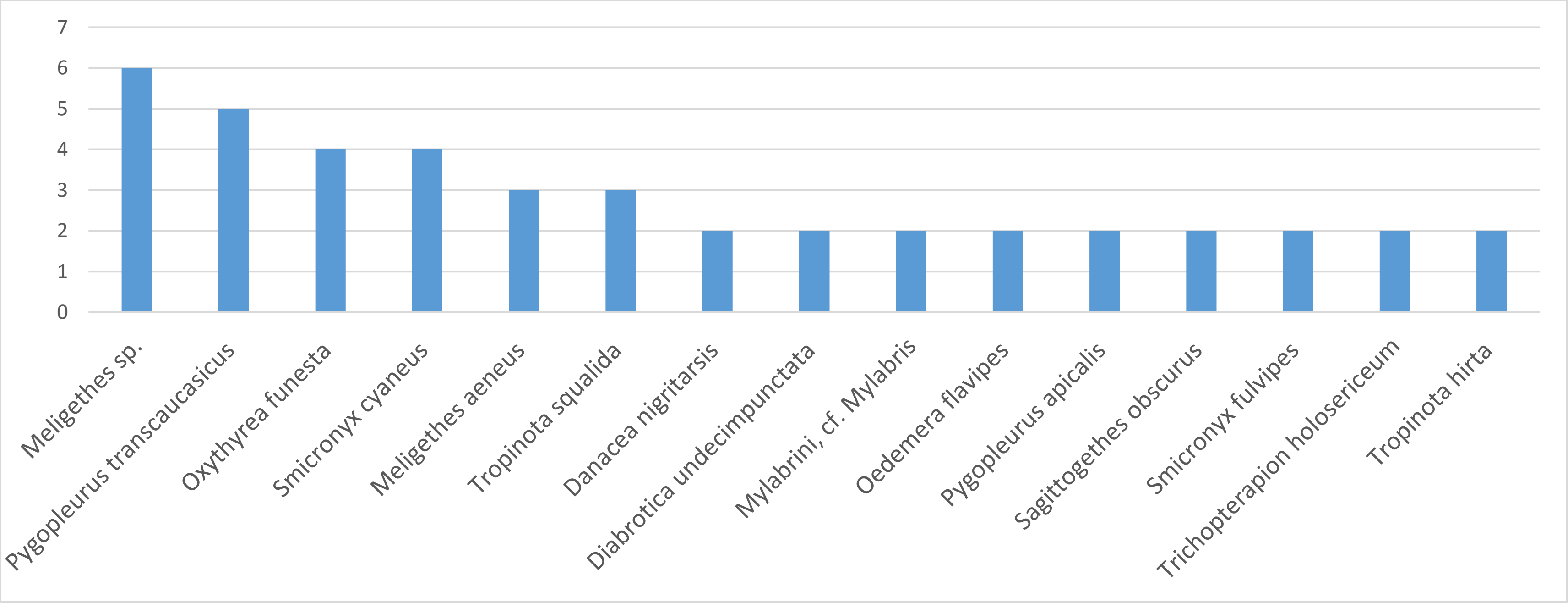

Figure S1.9. The most numerous observations (from 2 to 6) of species in Coleoptera.

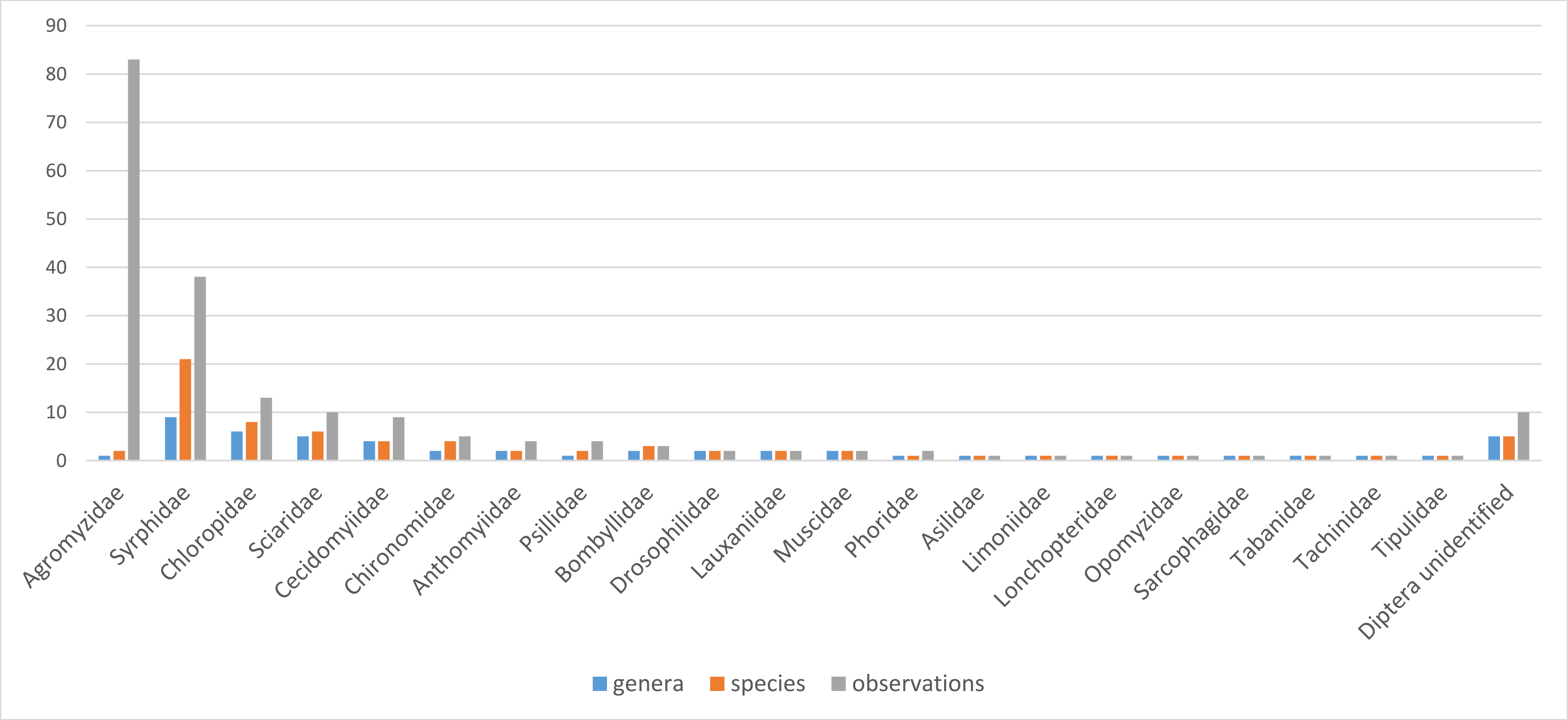

Figure S1.10. Number of genera, species and observations in families of Diptera.

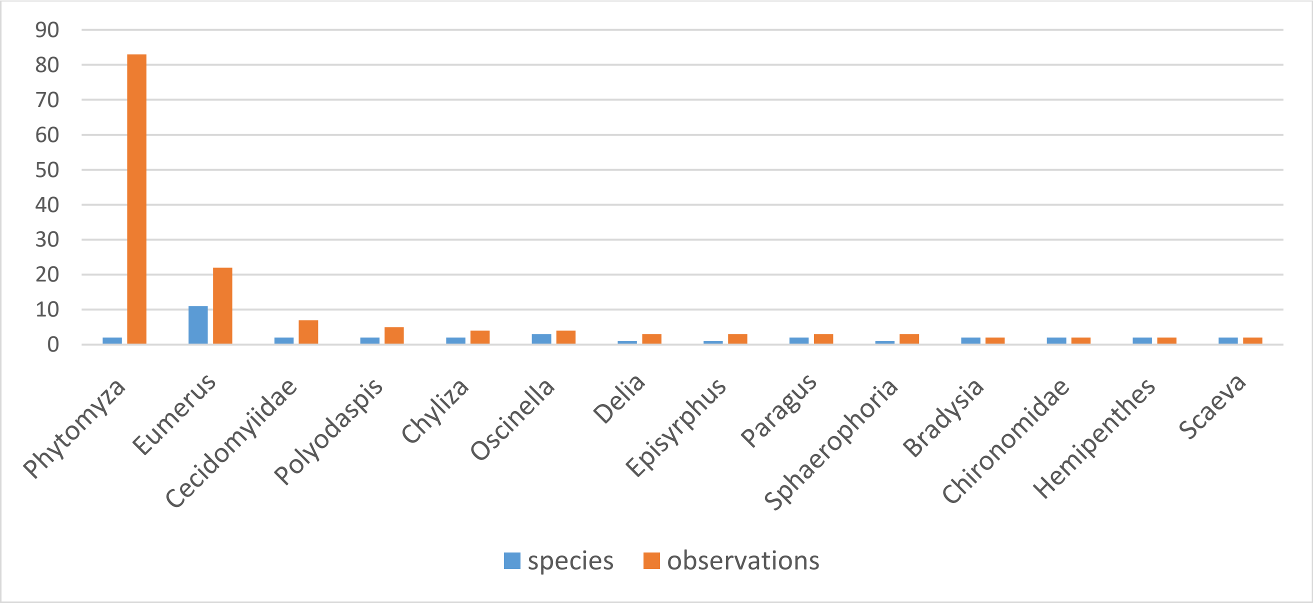

Figure S1.11. Number of species and observations (from 2 to 83) in the most numerous genera of Diptera.

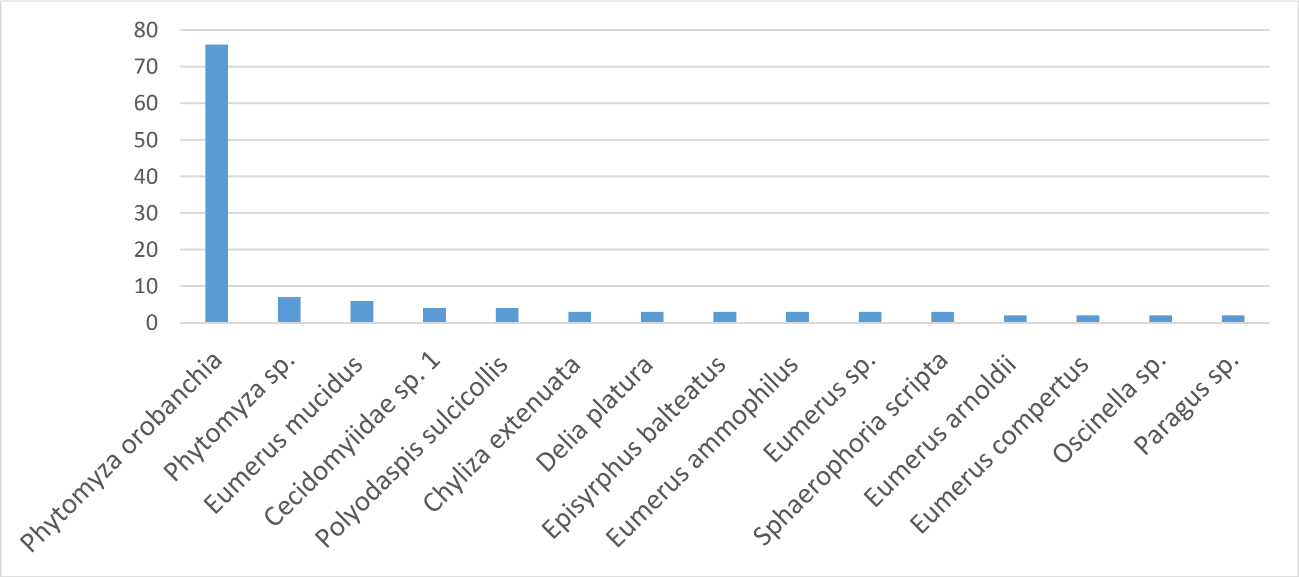

Figure S1.12. The most numerous observations (from 2 to 76) of species in Diptera.

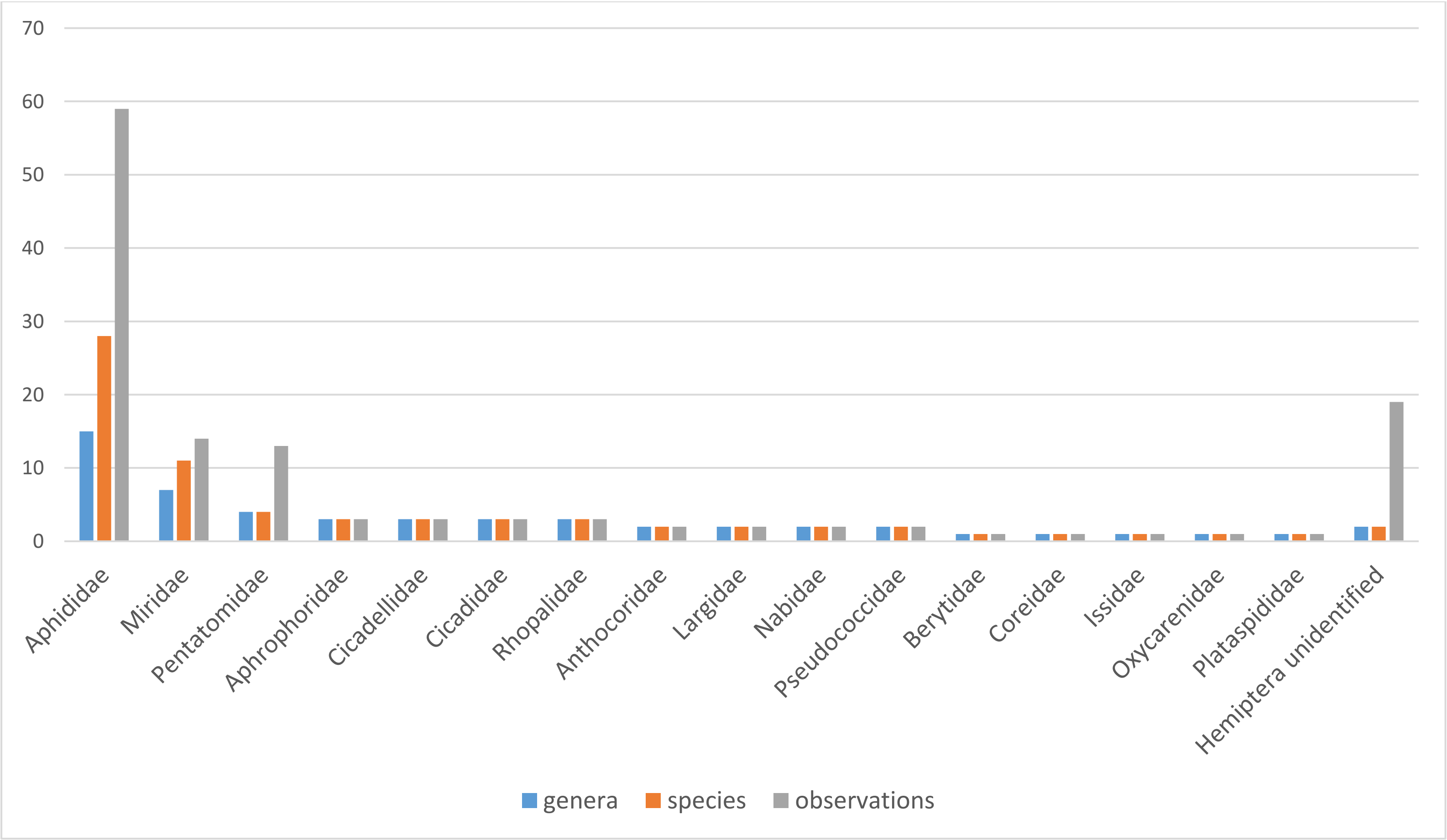

Figure S1.13. Number of genera, species and observations in families of Hemiptera.

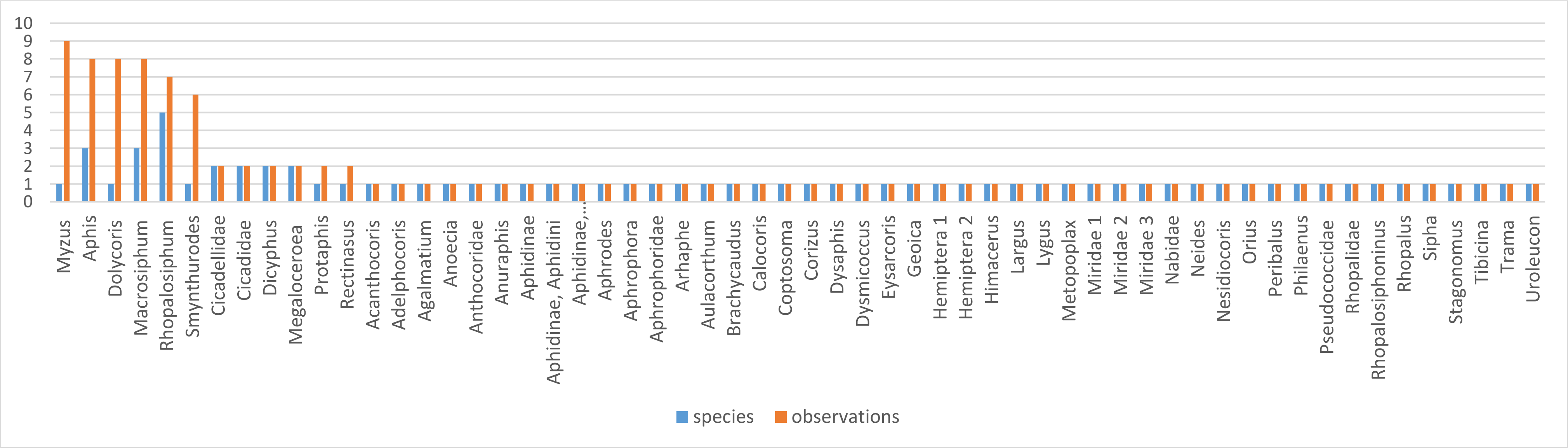

Figure S1.14. Number of species and observations of genera in Hemiptera.

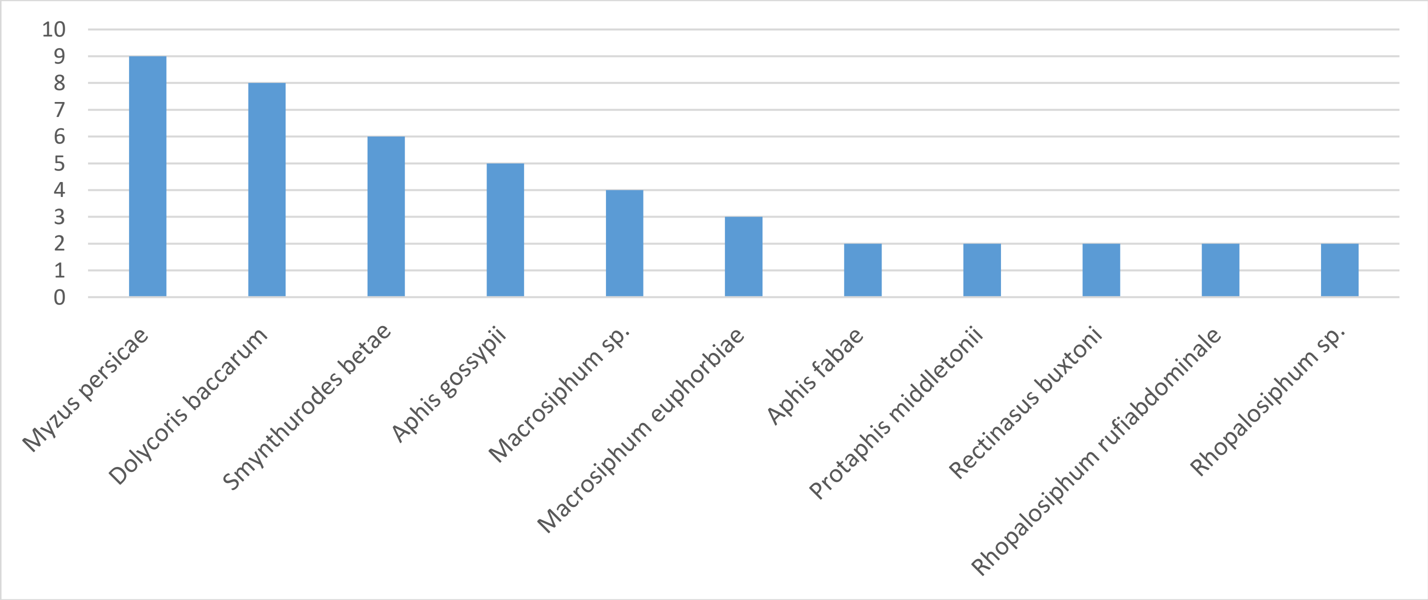

Figure S1.15. The most numerous observations (from 2 to 9) of species in Hemiptera.

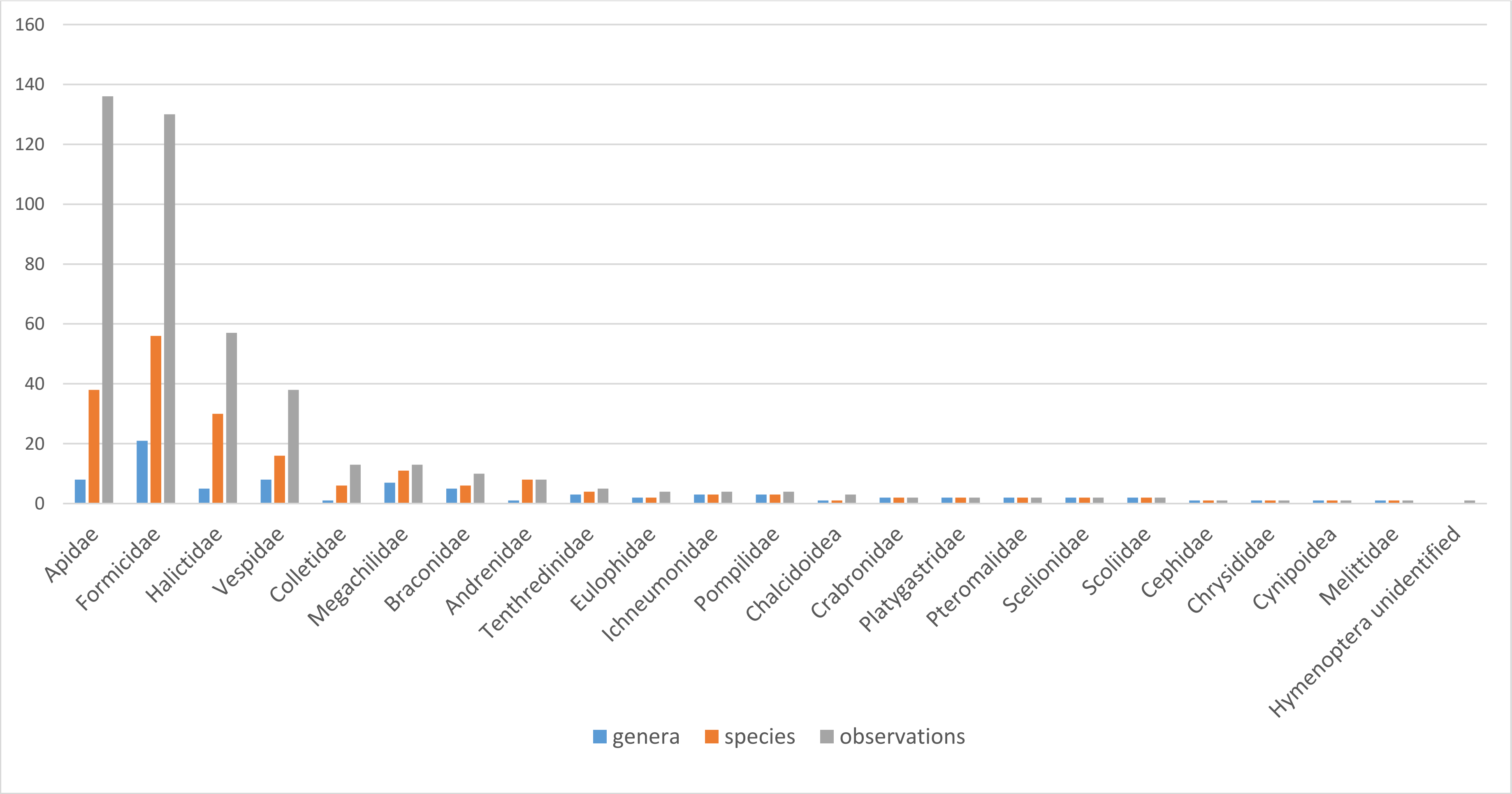

Figure S1.16. Number of genera, species and observations in families of Hymenoptera.

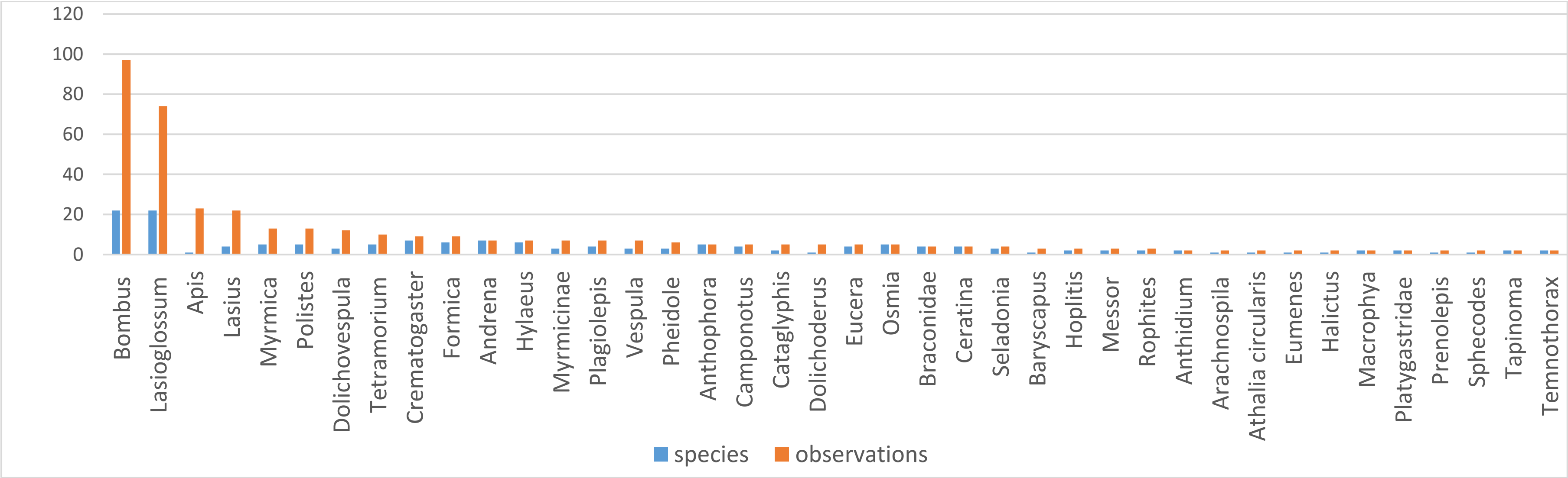

Figure S1.17. Number of species and observations (from 2 to 97) in the most numerous genera of Hymenoptera.

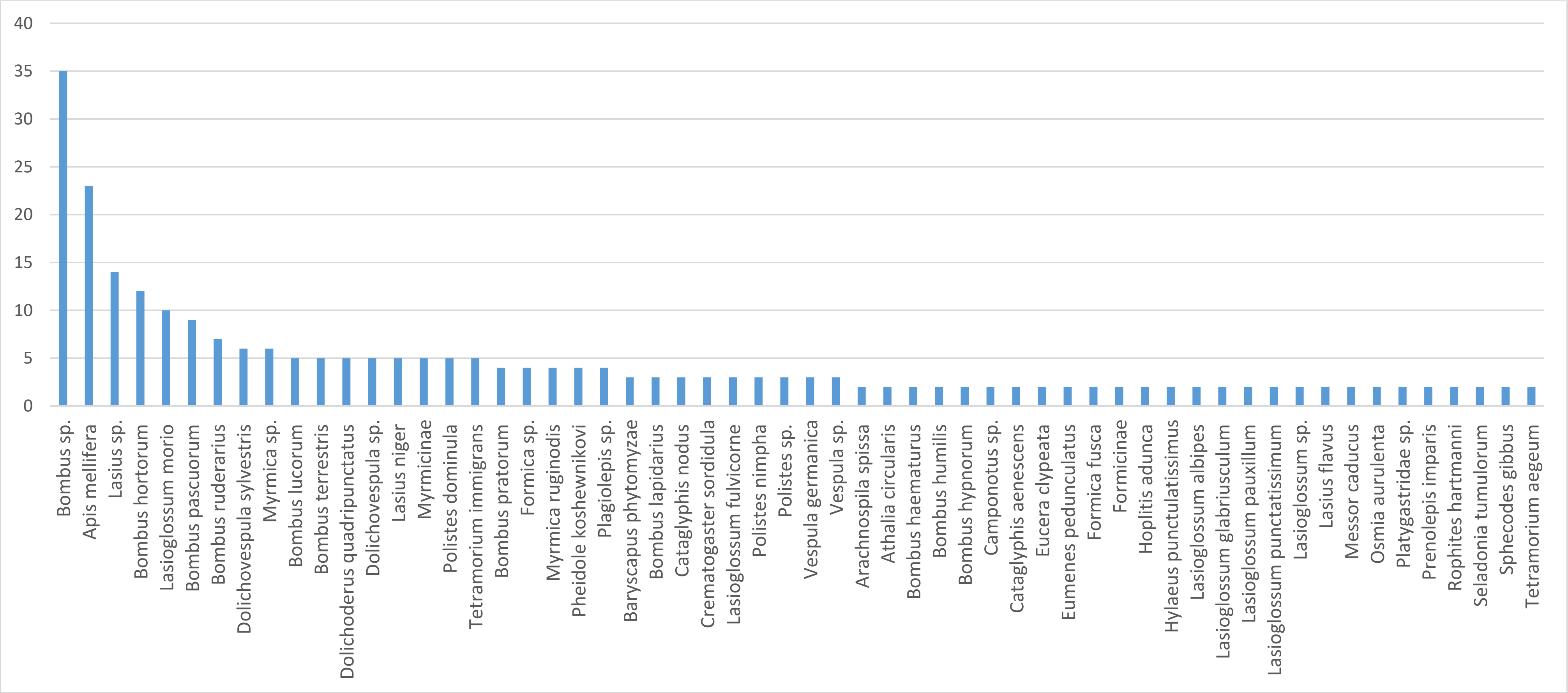

Figure S1.18. The most numerous observations (from 2 to 35) of species in Hymenoptera.

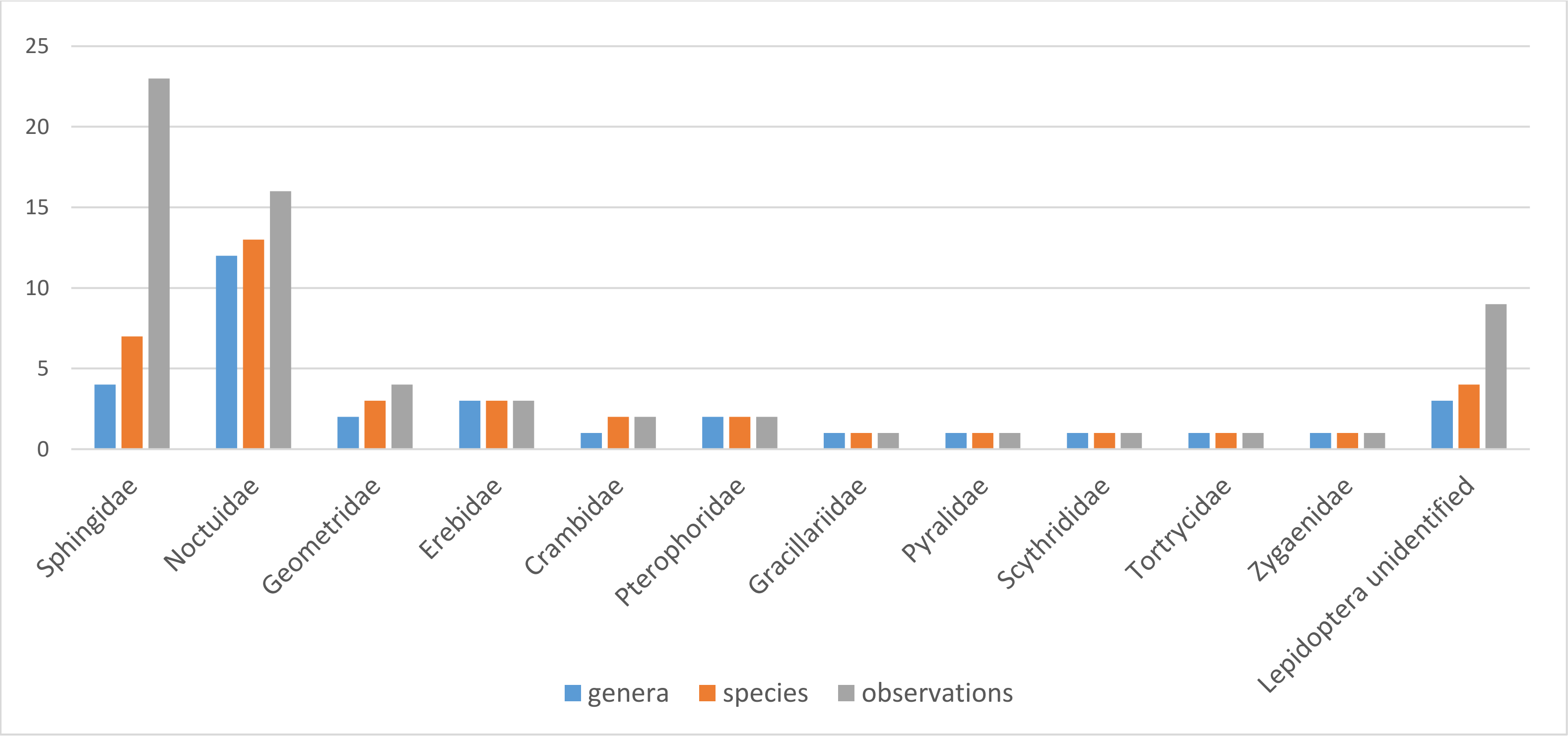

Figure S1.19. Number of genera, species and observations in families of Lepidoptera.

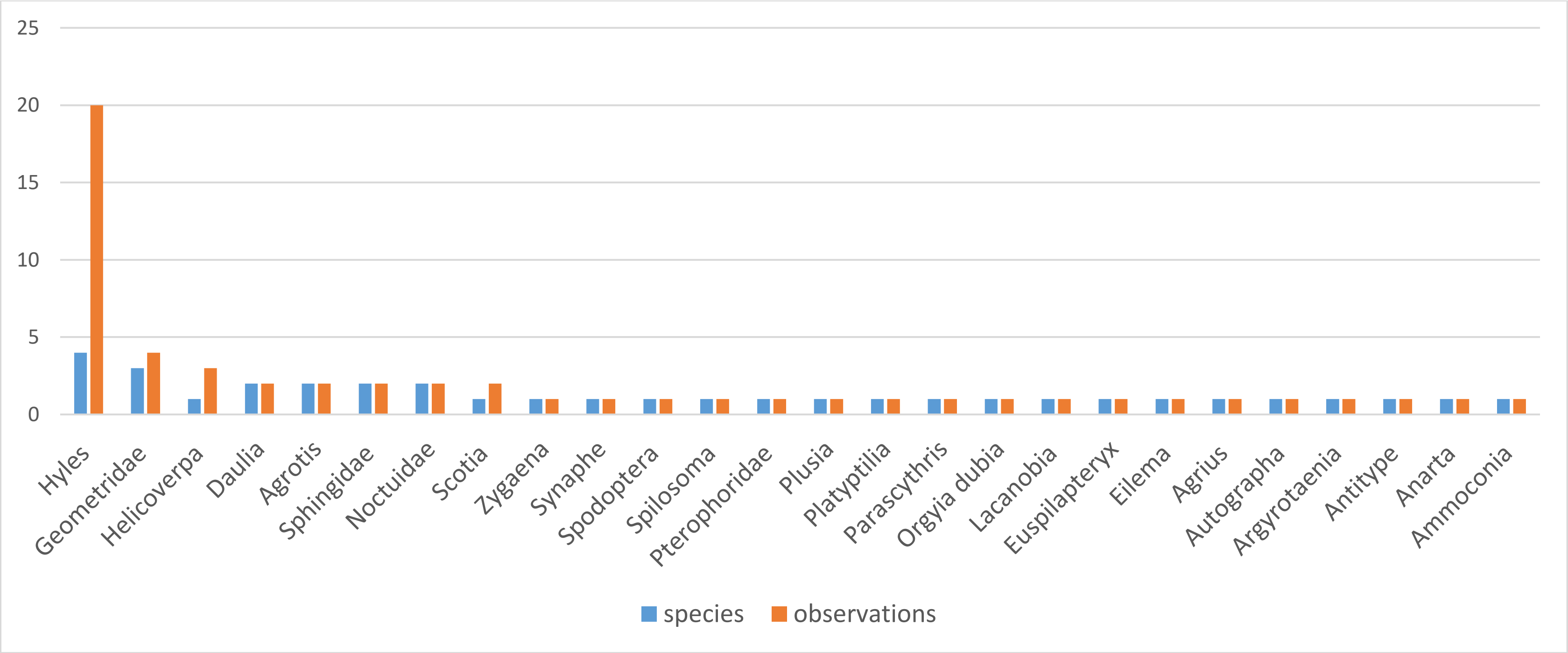

Figure S1.20. Number of species and observations in genera of Lepidoptera.

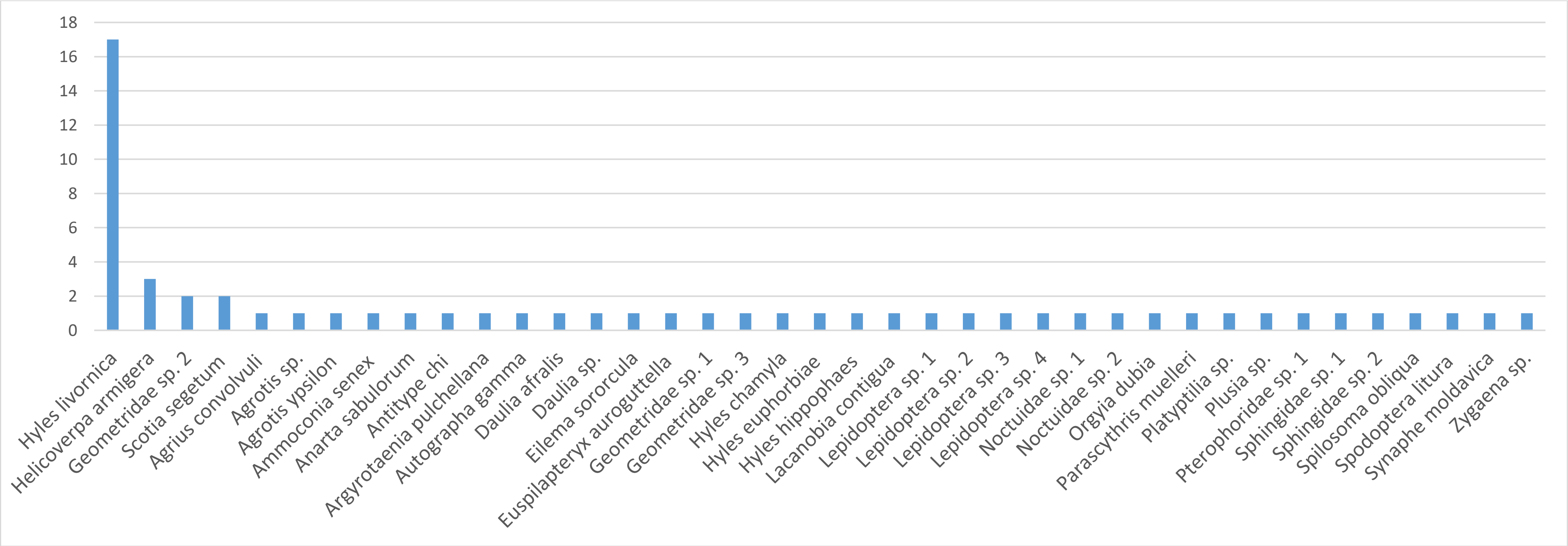

Figure S1.21. Number of observations of species in Lepidoptera.

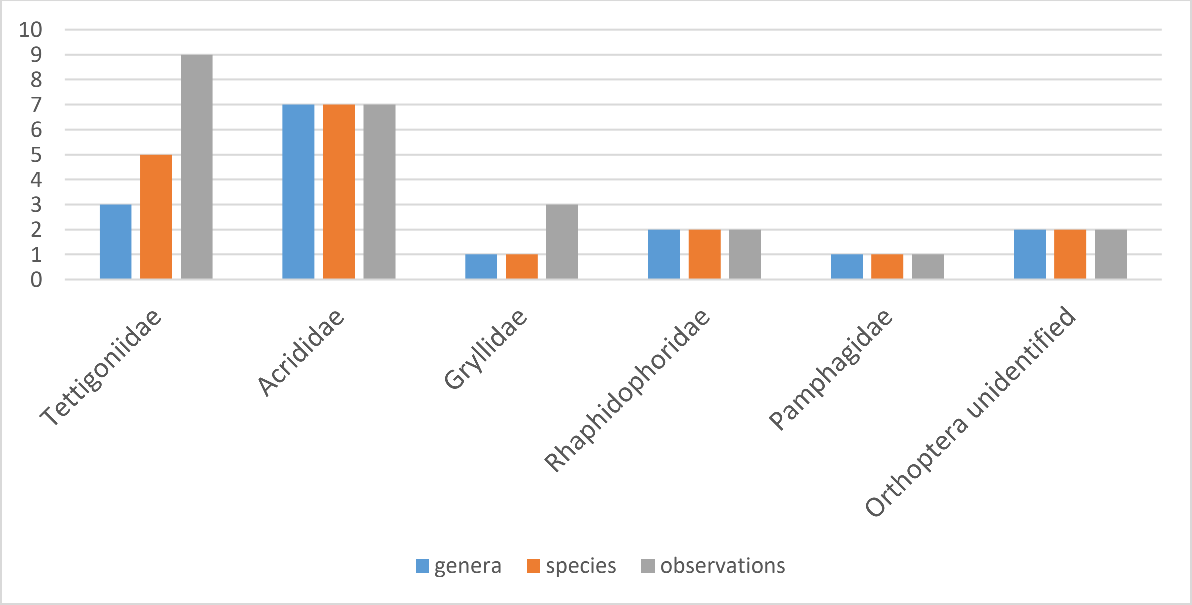

Figure S1.22. Number of genera, species and observations in families of Orthoptera.

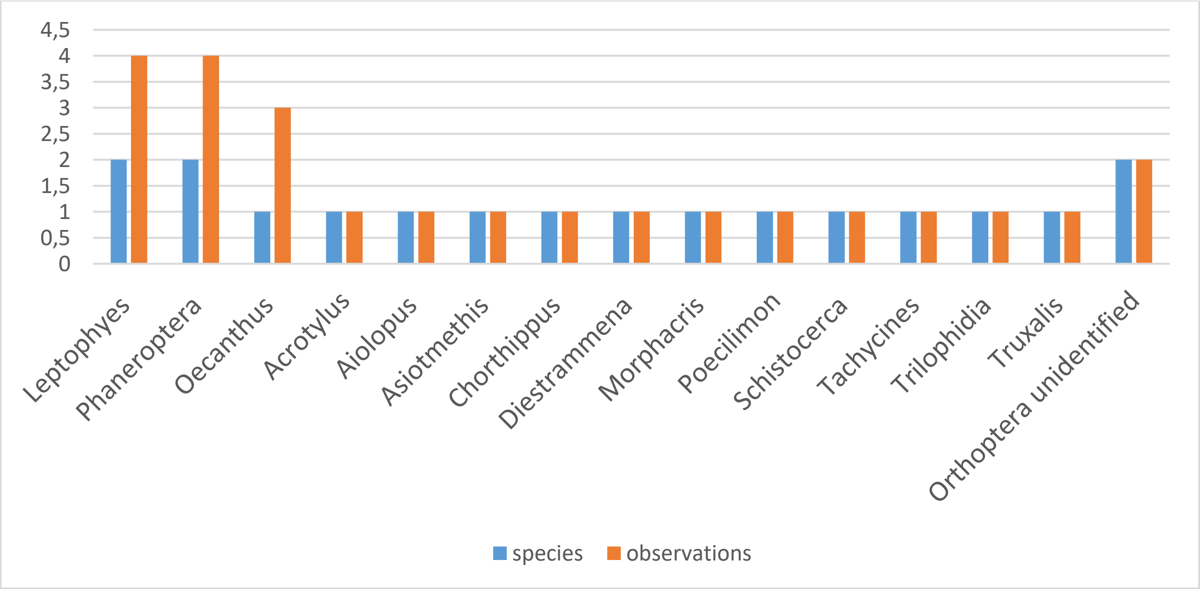

Figure S1.23. Number of species and observations in genera of Orthoptera.

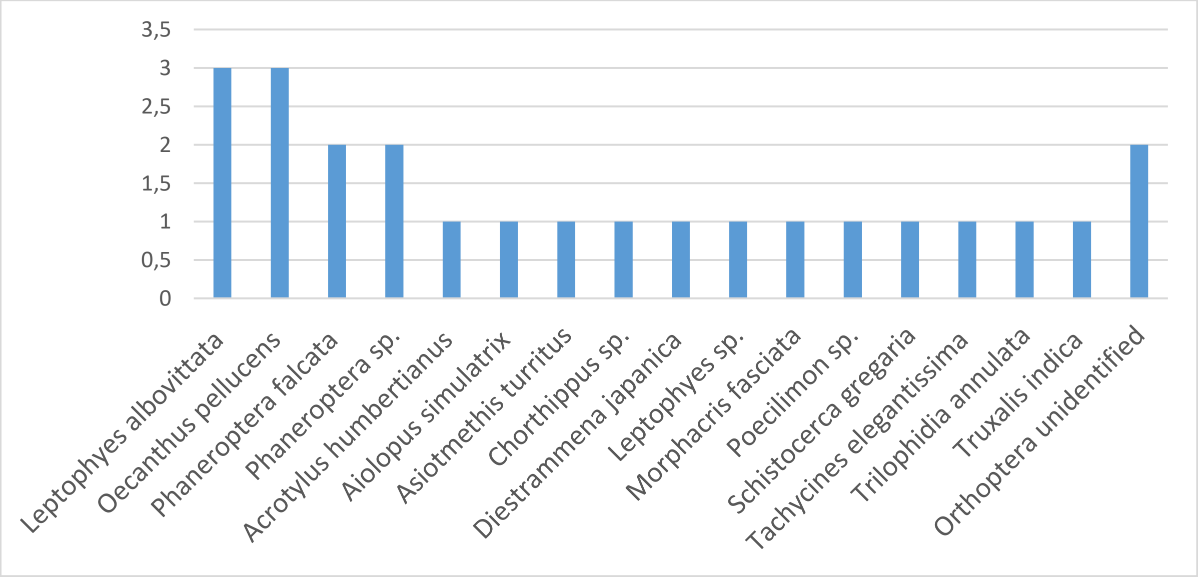

Figure S1.24. Number of observations of species in Orthoptera.

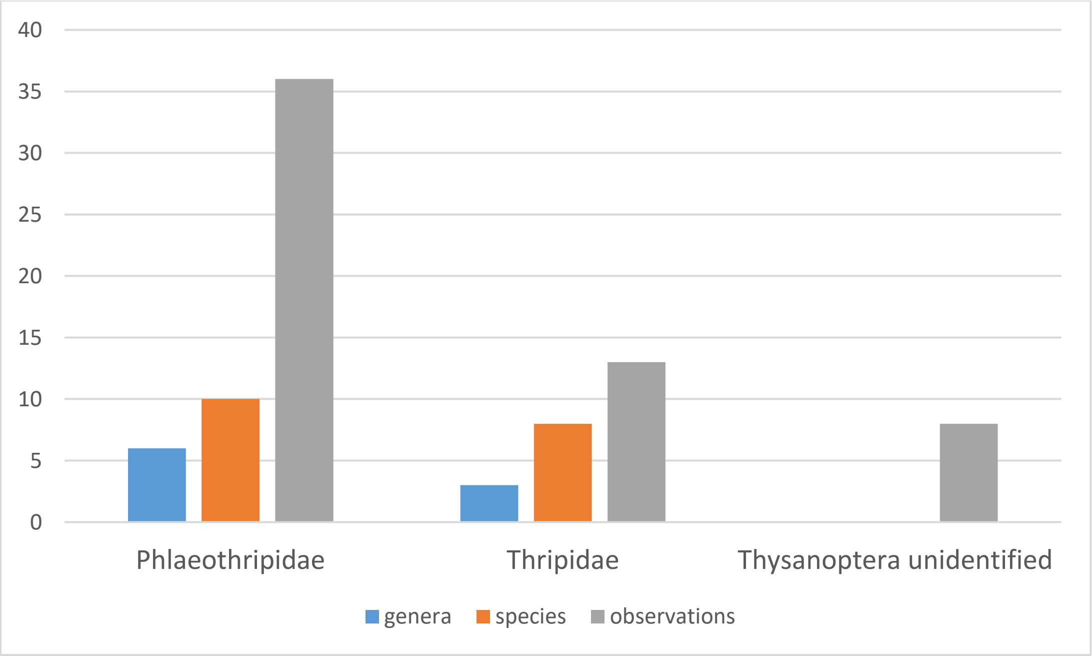

Figure S1.25. Number of genera, species and observations of families in Thysanoptera.

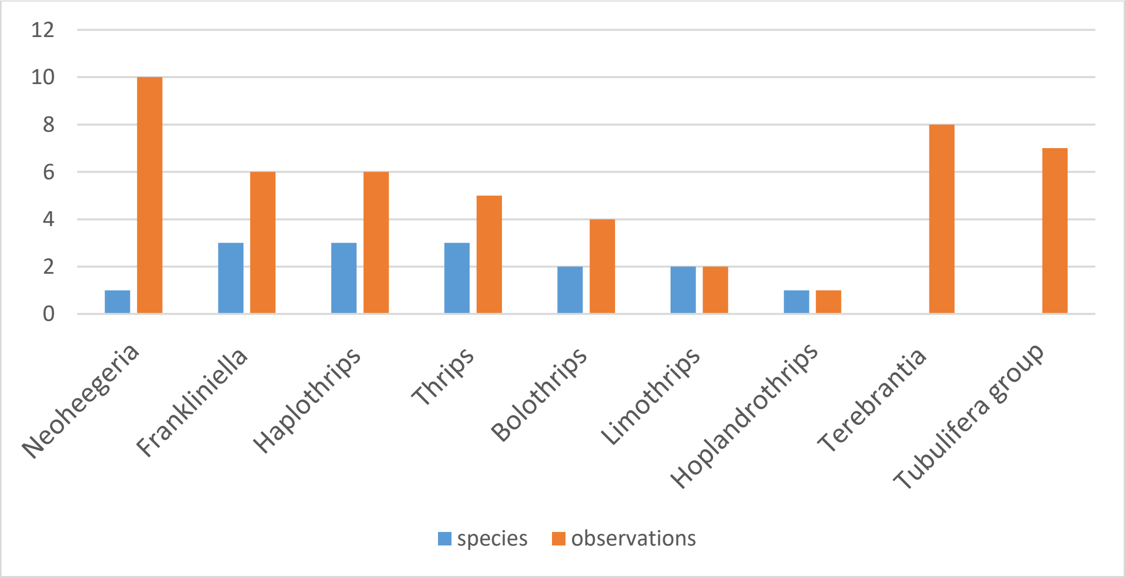

Figure S1.26. Number of species and observations of genera in Thysanoptera.

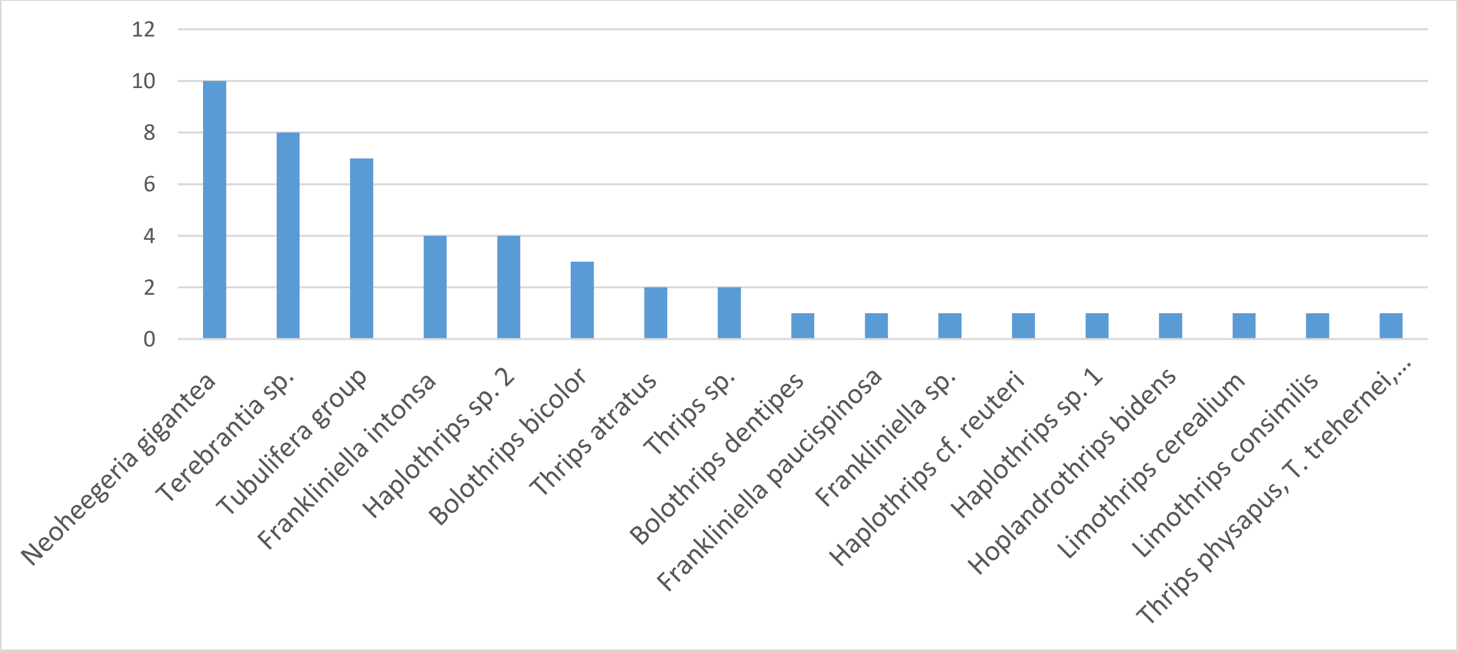

Figure S1.27. Number of observations of species in Thysanoptera.

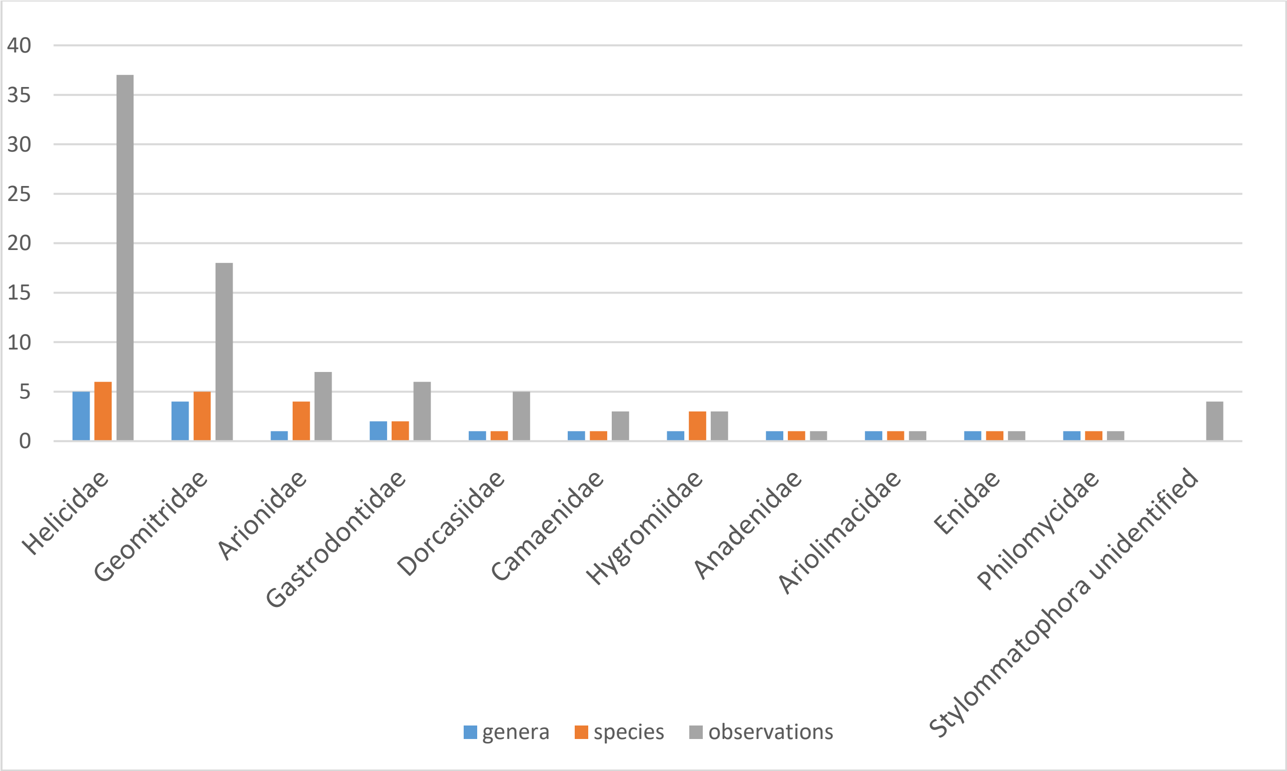

Figure S1.28. Number of genera, species and observations in families of Stylommatophora (Mollusca).

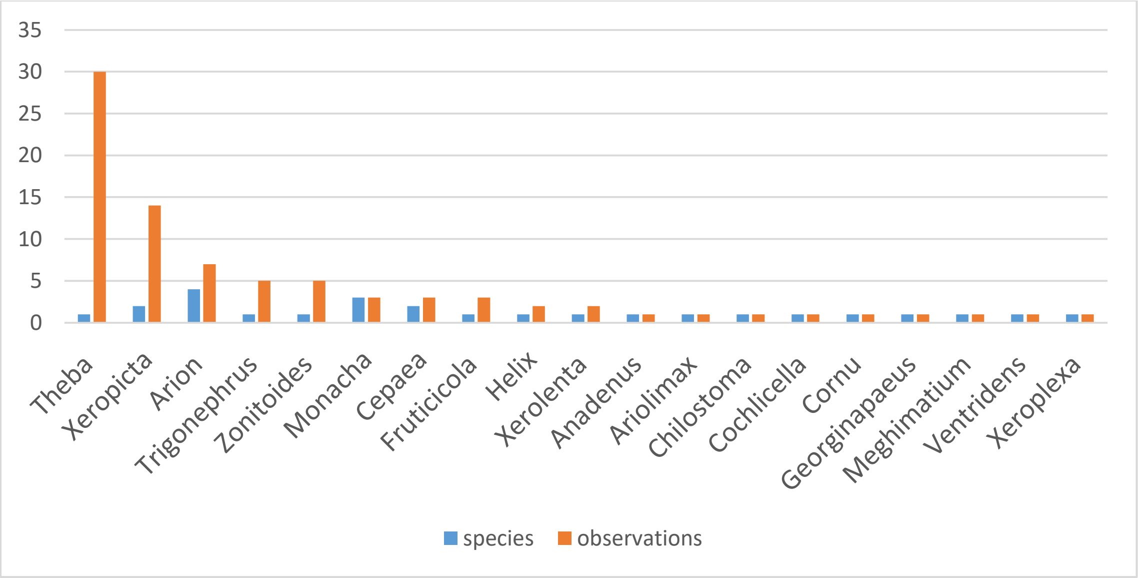

Figure S1.29. Number of species and observations of genera in Stylommatophora (Mollusca).

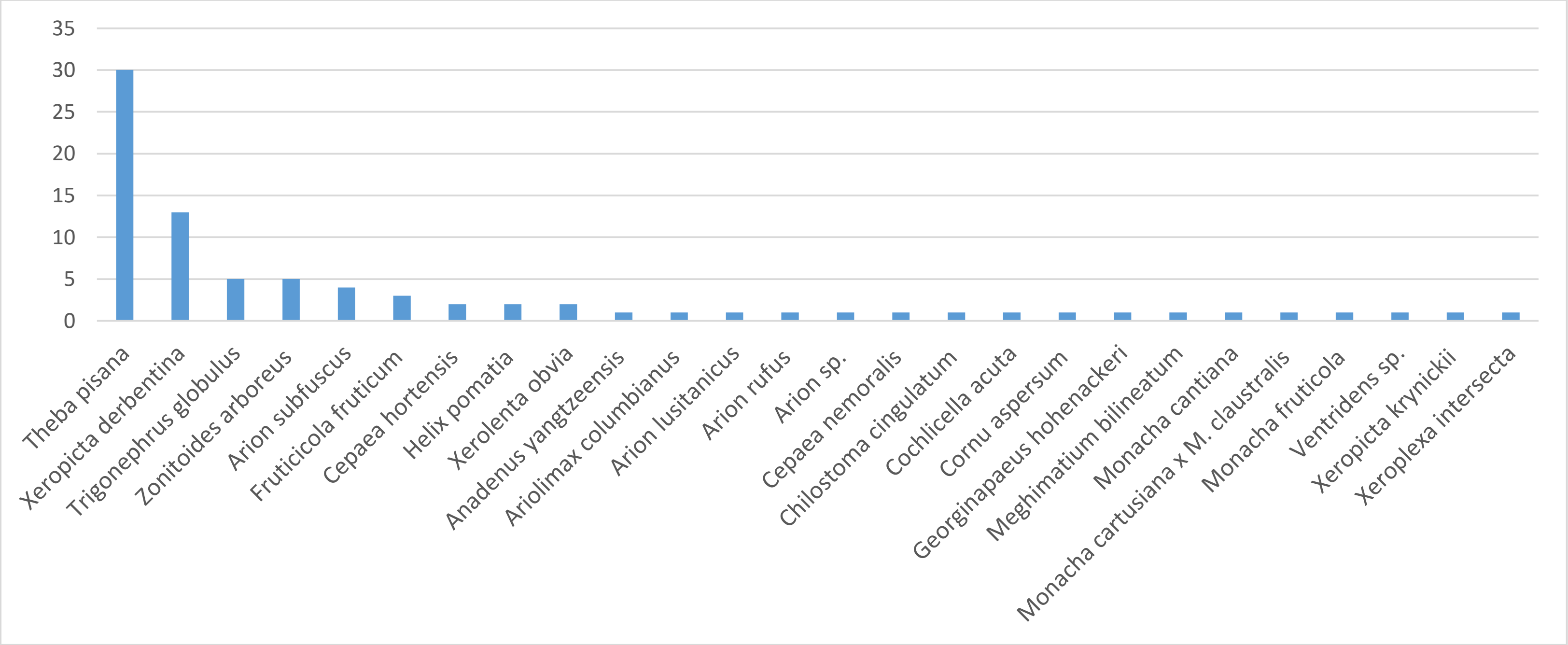

Figure S1.30. Number of observations of species in Stylommatophora (Mollusca).

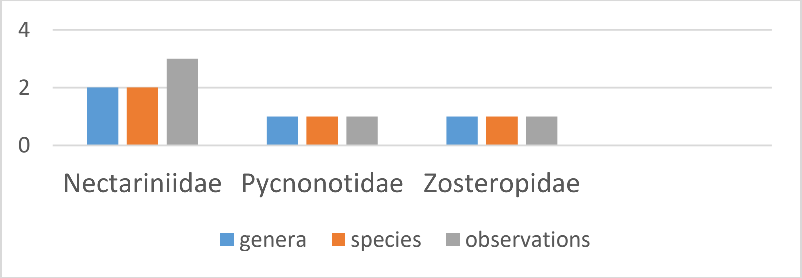

Figure S1.31. Number of genera, species and observations of families in Passeriformes (Aves).

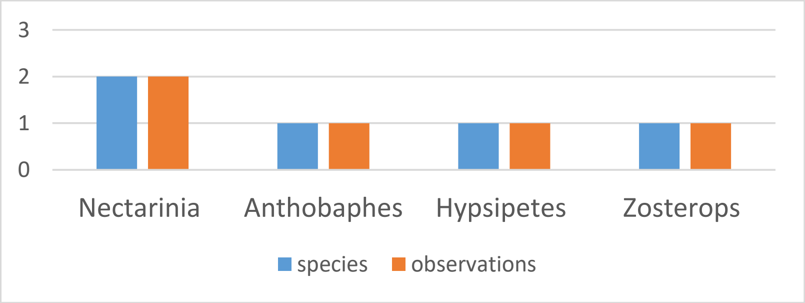

Figure S1.32. Number of species and observations in genera of Passeriformes (Aves).

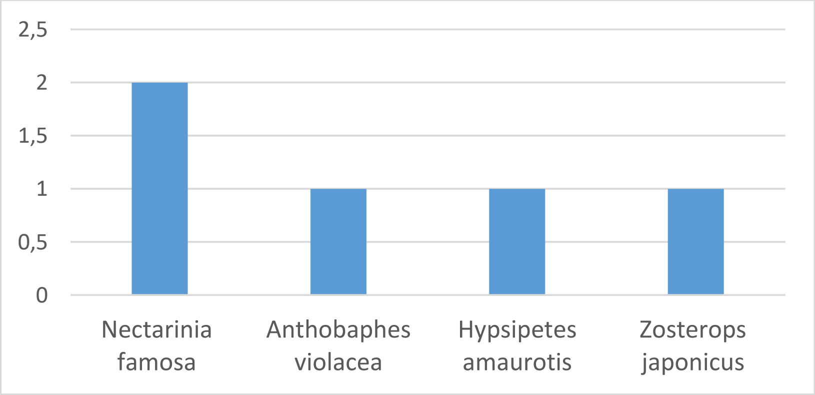

Figure S1.33. Number of observations of species in Passeriformes (Aves).

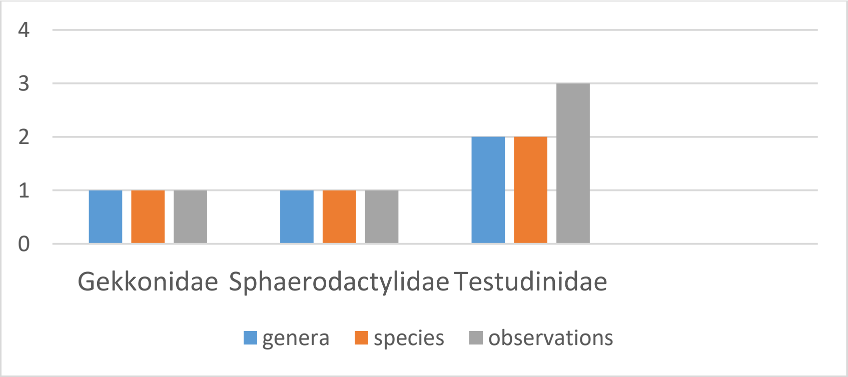

Figure S1.34. Number of families, genera, species and observations in Reptilia.

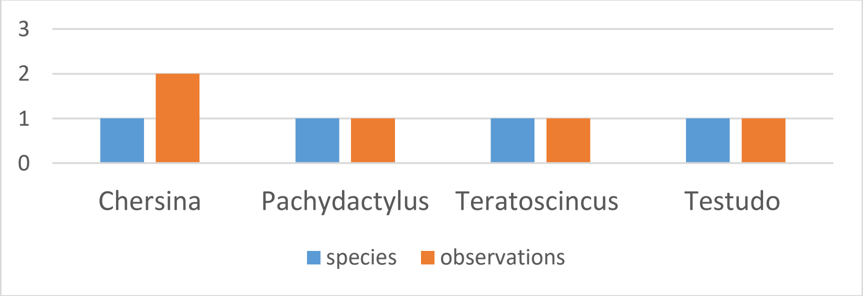

Figure S1.35. Number of species and observations in genera of Reptilia.

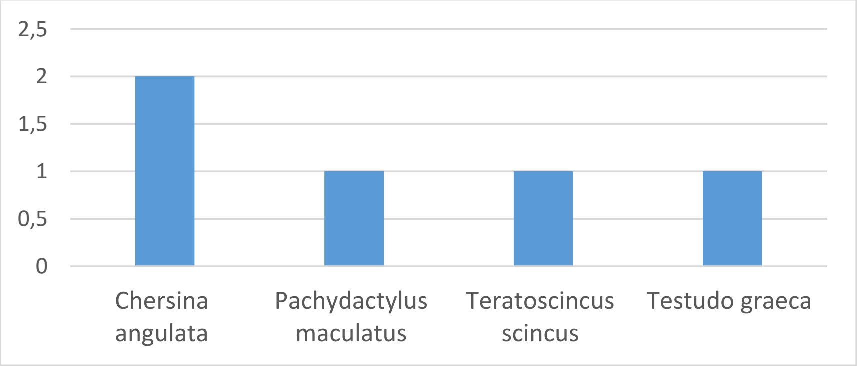

Figure S1.36. Number of observations of species in Reptilia.

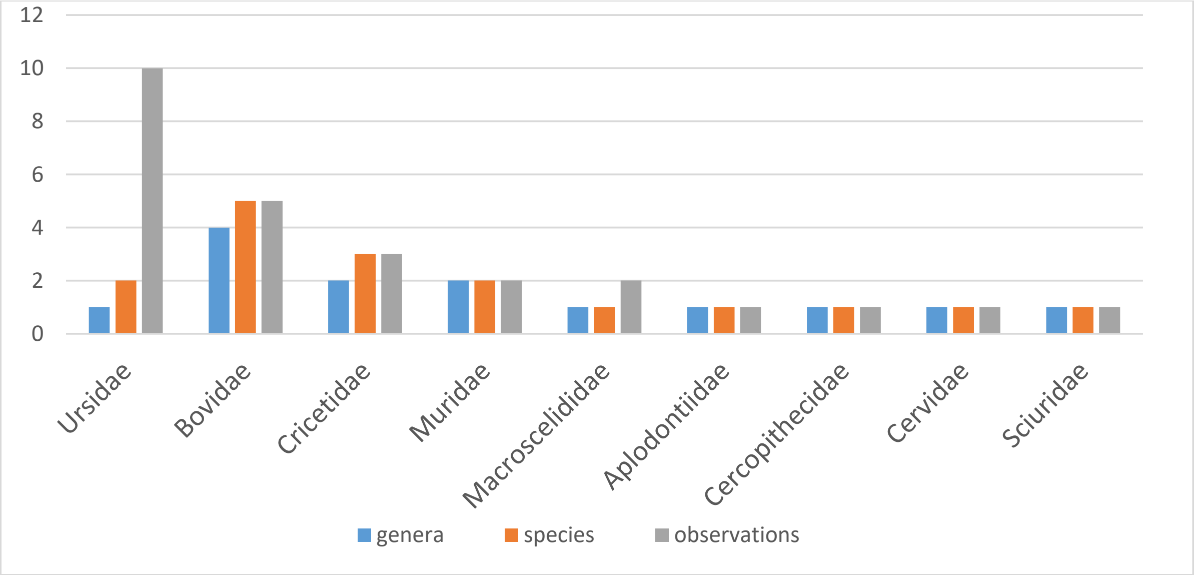

Figure S1.37. Number of genera, species and observations in families of Mammalia.

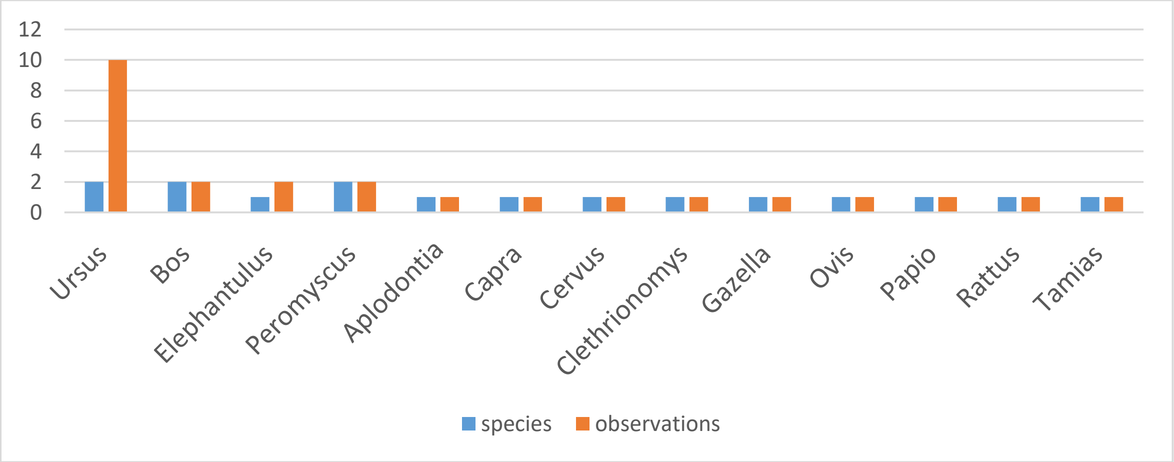

Figure S1.38. Number of species and observations in genera of Mammalia.

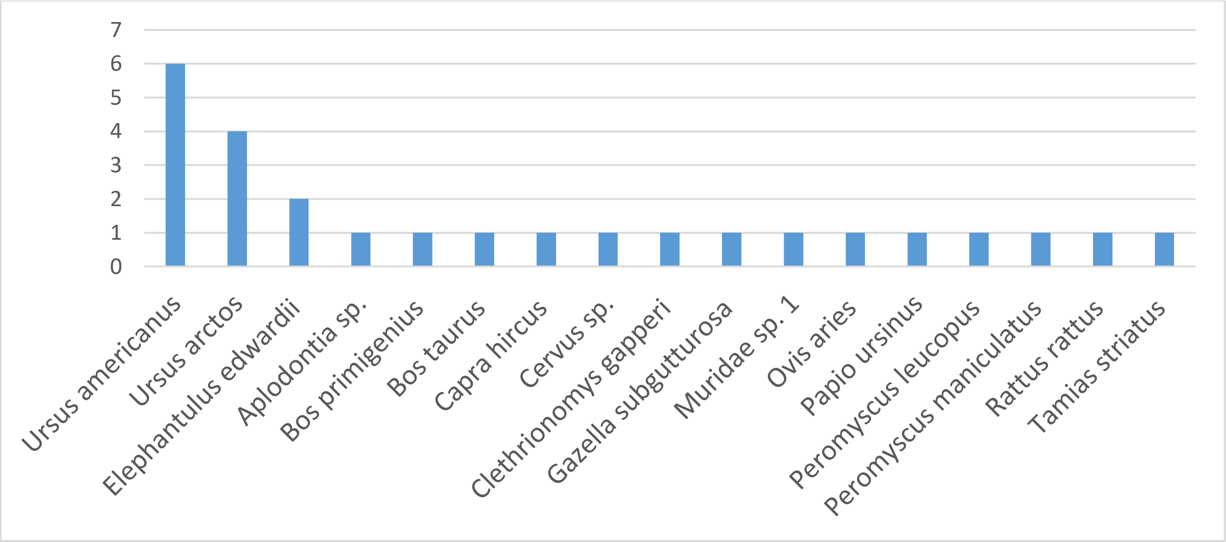

Figure S1.39. Number of observations of species of Mammalia.
