# Supplementary material for: A multiverse of trophic networks and coevolutionary trajectories among holoparasitic Orobanchaceae and their animal associates: a global perspective
Source: PhytoKeys. 2026 Jun 2;275:209–97. doi: 10.3897/phytokeys.275.192014 (PMC13250618; doi:10.3897/phytokeys.275.192014)
Supplement: Supplementary material 2 — Supplementary photographic boards of orders of recorded fauna [file phytokeys-275-209_article-192014__-s002.pdf]

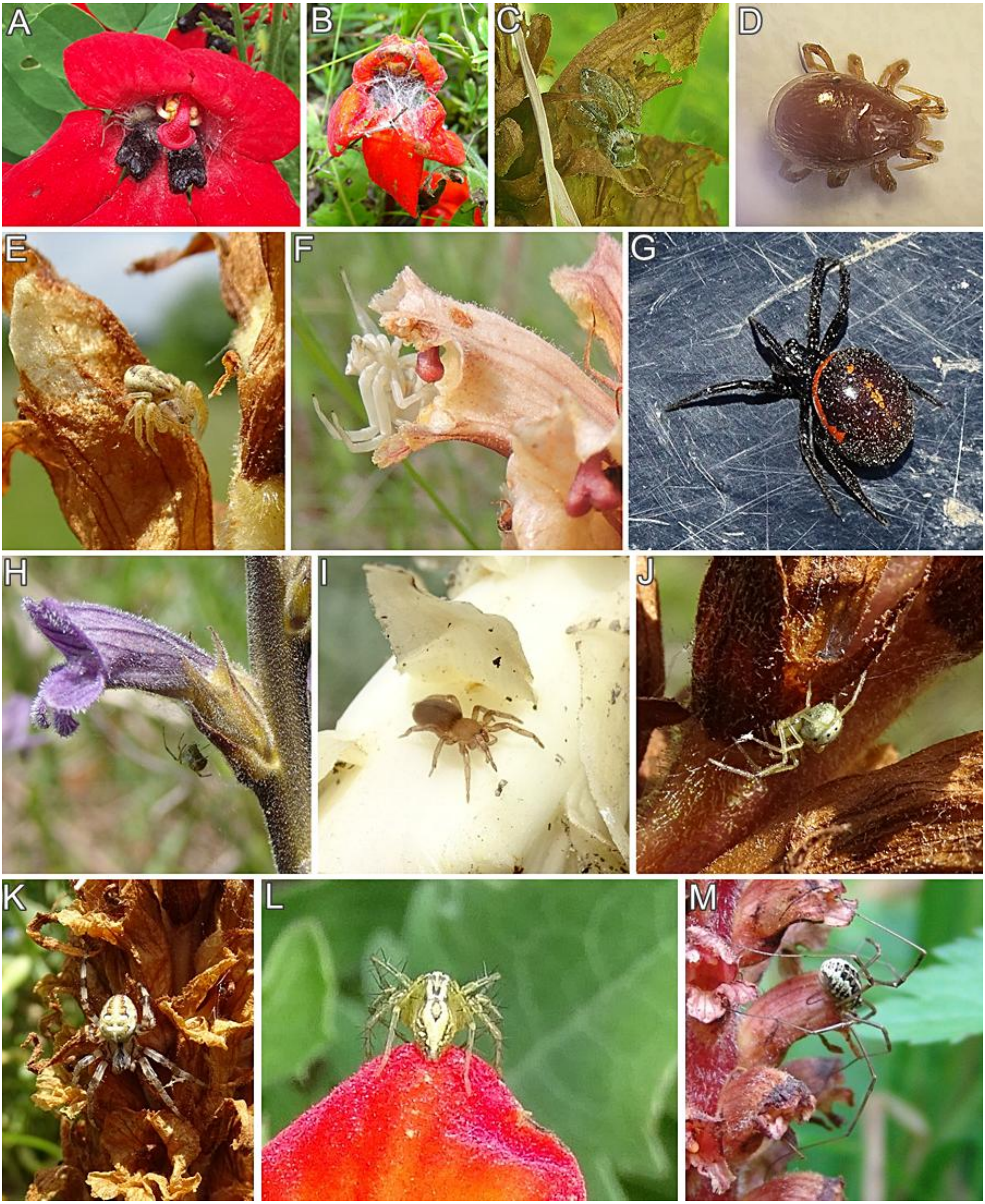

Figure S2.1. Representative species of Arachnida. A: *Cheiracanthium pennyi* (Cheiracanthiidae) on *Phelypaea tournefortii*, Armenia; B: silk retreat of *Cheiracanthium* sp. (Cheiracanthiidae) on *P. coccinea*, Georgia; C: *Evarcha falcata* (Salticidae) on *Orobanche alsatica*, Poland; D: Pergamasinae (Parasitidae, Mesostigmata) from *O. alsatica*, Poland; E: *Xysticus cristatus* (Thomisidae) on *O. caryophyllacea*, Poland; F: *Thomisus onustus* (Thomisidae) on *O. alba*, Bulgaria; G: *Stetoda paykulliana* (Theridiidae) on *P. portoilicitana*, Armenia; H: *Phylloneta impressa* (Theridiidae) on *Phelipanche purpurea*, Poland; I: *Cheiracanthium* sp. (Cheiracanthiidae) on *Cistanche fissa*, Azerbaijan; J: *Enoplognatha ovata* (Theridiidae) on *O. lutea*, Poland; K: *Araneus quadratus* (Araneidae) on *O. schelkovnikovii*, Armenia; L: *Oxyopes lineatus* (Oxyopidae) on *P. coccinea*, Georgia; M: Opiliones on *O. flava*, Poland; Phot. R. Piwowarczyk (A –E, G–M), A. Mátis (F).

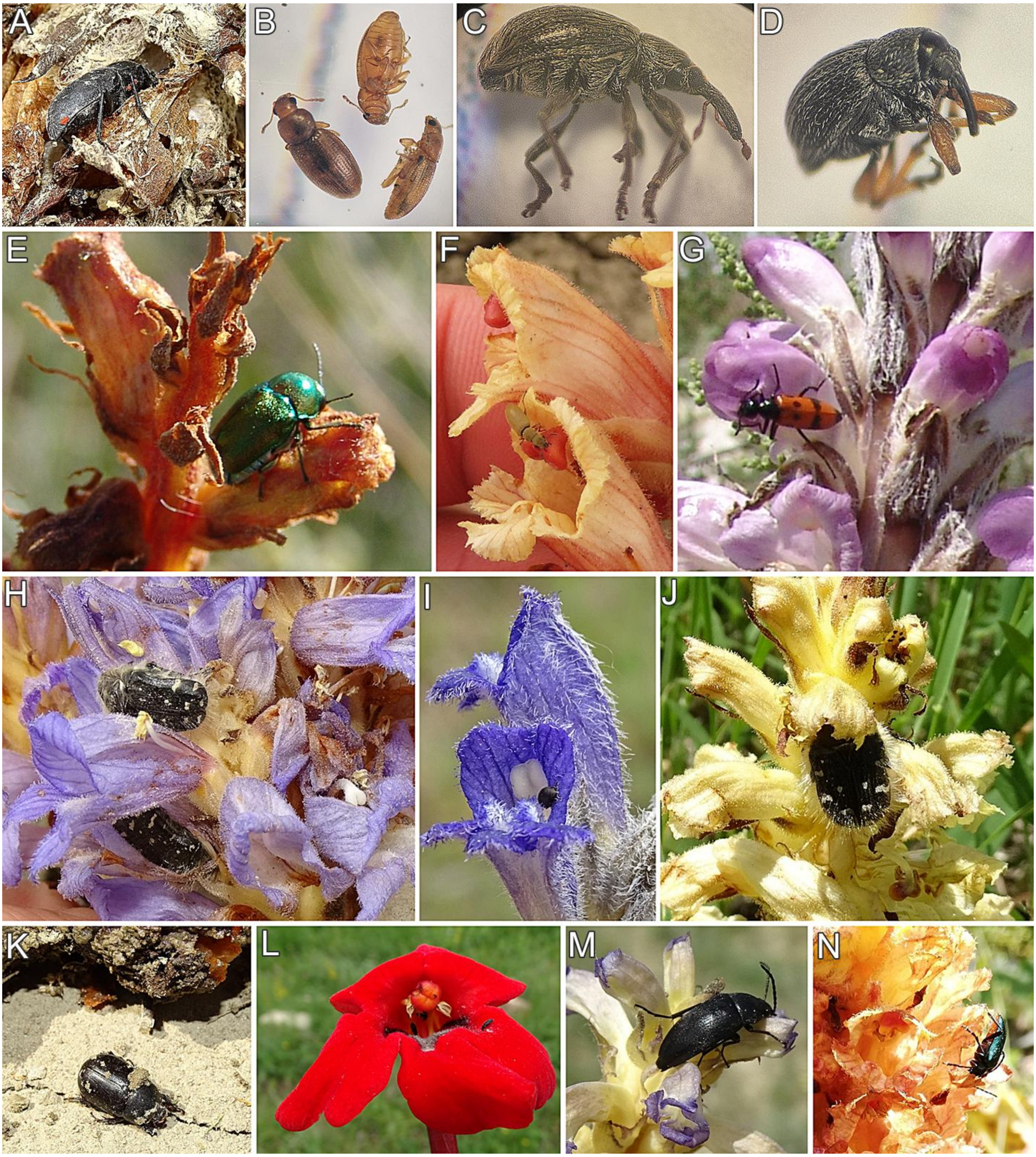

Figure S2.2. Representative species of Coleoptera. A: cf. *Hedyphanes* sp. (Tenebrionidae) with attached red predatory mites from Parasitidae (Mesostigmata) on *Cistanche fissa*, Azerbaijan; B: *Corticaria obscura*, *Corticarina truncatella* (Latridiidae) from *Orobanche picridis*, Poland; C: *Trichopterapion holosericeum* (Apionidae) from *O. laxissima*, Georgia; D: *Exapion difficile* (Apionidae) from *O. alsatica*, Poland; E: *Cryptocephalus* sp. (Chrysomelidae) on *O. gracilis*, Austria; F: *Danacea nigritaris* (Melyridae) on *O. alba*, Romania; G: *Hycleus scabiosae* (Meloidae) on *C. armena*, Armenia; H: *Tropinota senicula* (Scarabaeidae) on *Phelipanche caesia*, Azerbaijan; I: *Sagittogethes disctinctus* (Nitidulidae) on *P. pulchella*, Armenia; J: *Oxythyrea* sp. (Scarabaeidae) on *O. caryophyllacea*, Georgia; K: *Pentodon* sp. (Scarabaeidae) on *C. flava*, Azerbaijan; L: Coleoptera on *Phelypaea coccinea*, Armenia; M: *Podonta* sp. (Tenebrionidae) on *O. cumana*, Georgia; N: *Gaurotes virginea* (Cerambycidae) on *O. flava*, Romania. Phot. R. Piwowarczyk (A –E, G–M), A. Mátis (F, N).

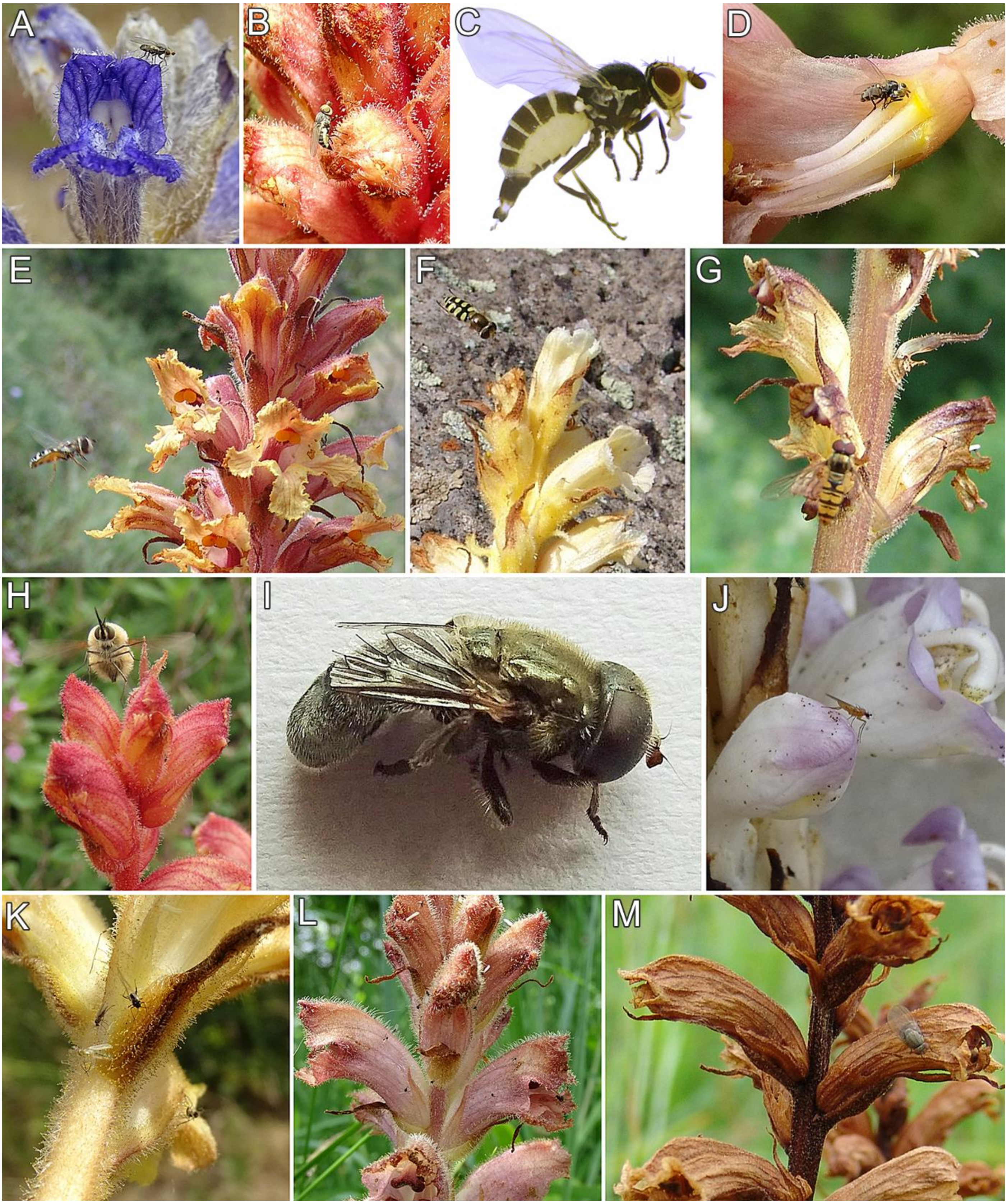

Figure S2.3. Representative species of Diptera. *Phytomyza orobanchia* (Agromyzidae) on A: *Phelipanche pulchella*, Armenia; B, D: *Orobanche centaurina*, Poland; C: *P. ramosa*, Poland; E: *Scaeva pyrastri* (Syrphidae) on *O. kurdica*, Armenia; F: *Eupeodes* cf. *luniger* (Syrphidae) on *Orobanche* sp., Armenia; G: *Episyrphus balteatus* (Syrphidae) on *O. reticulata*, Poland; H: *Bombylius cinerascens* (Bombyliidae) on *O. alba*, Romania; I: *Eumerus mucidus* (Syrphidae) on *Cistanche armena*, Armenia; J: *Lonchoptera* sp. (Lonchopteridae) on *C. flava*, Azerbaijan; K: Chironomidae and *Pseudolycoriella* cf. *morenae* (Sciaridae) trapped in glandular trichomes of *O. caryophyllacea*, Georgia; L: *Sciaridae* trapped in glandular trichomes of *O. caryophyllacea*, Poland; M: *Minettia* sp. (Lauxaniidae) on *O. lutea*, Poland. Phot. R. Piwowarczyk (A –B, D–G, I–M), Ł. Mielczarek (C), A. Mátis (H).

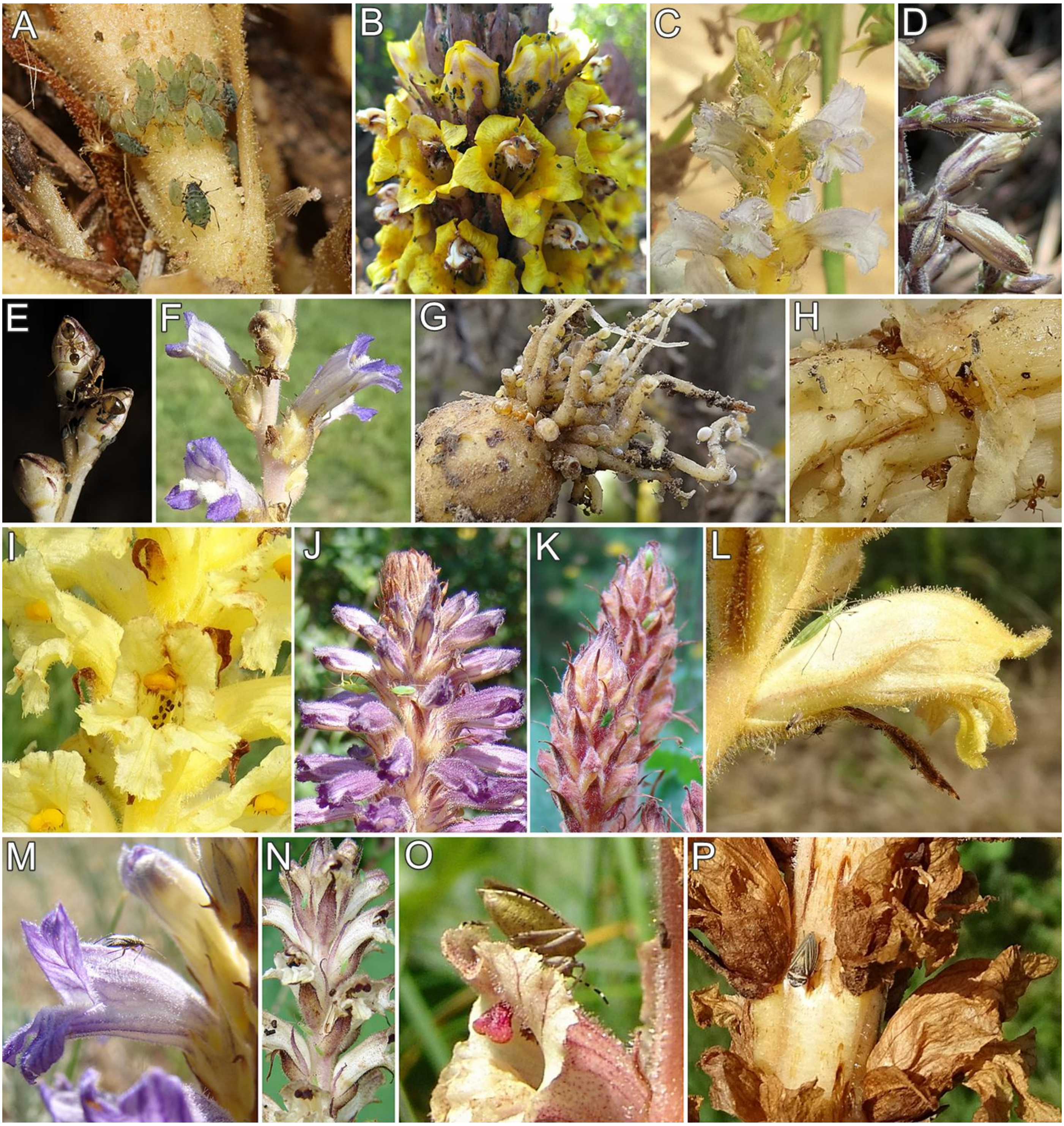

Figure S2.4. Representative species of Hemiptera. A: *Protaphis middletonii* (Aphididae) on *Orobanche* s.l., USA; B: Aphidini (Aphididae) on *Cistanche tubulosa*, India; C: *Macrosiphum euphorbiae* (Aphididae) on *Phelipanche ramosa*, Poland; D: *Macrosiphum* sp. (Aphididae) on *Aphyllon pinorum*, USA; E: *Dysaphis* aff. *middletoni* (Aphididae) with *Prenolepis* cf. *imparis* (Formicidae), trophobiosis, on *Epifagus virginiana*, USA; F: *Rhopalosiphum* sp. (Aphididae) on *P. aegyptiaca*, Georgia; G: *Smynthurodes betae* (Aphididae) with *Lasius niger* (Formicidae), trophobiosis, on haustoria of *P. ramosa*, Poland; H: *Rectinasus buxtoni* (Aphididae) with *Pheidole koshewnikovi* (Formicidae), trophobiosis, on underground stem of *C. fissa*, Azerbaijan; I: *Rhopalosiphum* sp. (Aphididae) on filaments and style in flower of *O. alsatica*, Poland; J: Hemiptera larvae on *P. lavandulacea*, Montenegro; K: cf. *Calocoris* sp. (Miridae) on *O. flava*, Poland; L: *Megaloceroea* sp. on *O. caryophyllacea*, Georgia; M: Miridae on *P. arenaria*, North Macedonia; N: Miridae on *O. reticulata*, Poland; O: Pentatomidae on *O. alba*, Poland; P: Cicadellidae on *O. schelkovnikovii*, Armenia. Phot. R. Piwowarczyk (C, F–L, N–P), Z. Nikolov (M), (A) photo by James Bailey from the iNaturalist web-site (https://www.inaturalist.org/photos/9344217), (B) photo by wonderviewer from the iNaturalist web-site (https://www.inaturalist.org/photos/16935937), (D) photo by ikap from the iNaturalist web-site (https://www.inaturalist.org/observations/229828025), (E) photo by Kimberly R Fleming from the iNaturalist web-site (https://www.inaturalist.org/photos/252434997), A, B, D, E: distributed under the terms of the Creative Commons CC BY-NC 4.0 license (https://creativecommons.org/licenses/by-nc/4.0/).

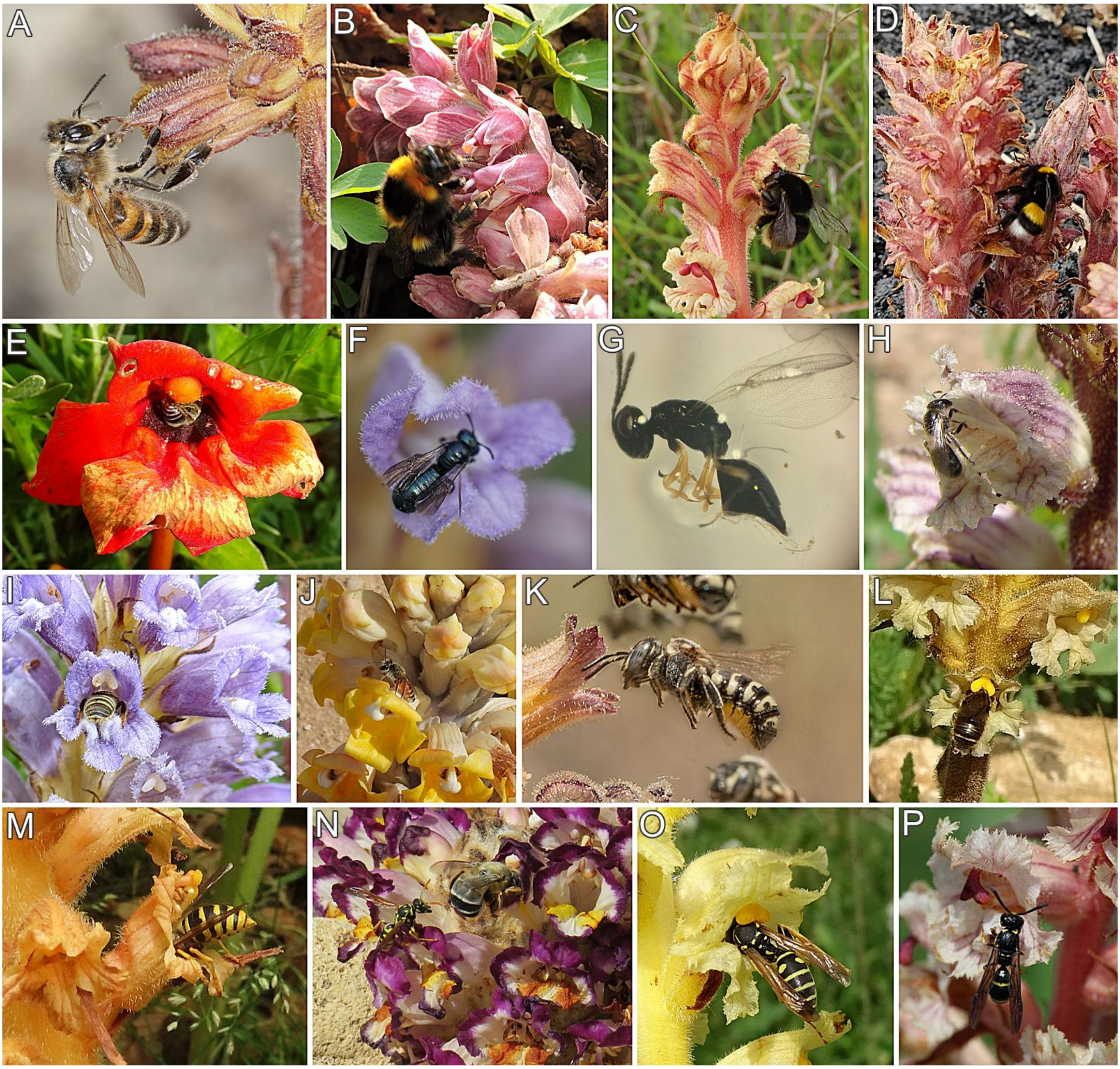

Figure S2.5. Representative species of Hymenoptera (Aculeata). A: *Apis mellifera* (Apidae) on *Orobanche laxissima*, Russia; B: *Bombus hortorum* (Apidae) on *Lathraea squamaria*, Romania; C: *B. humilis* (Apidae) on *O. alba*, Romania; D: *Bombus* sp. on *O. rapum-genistae*, Sicily; E: *Eucera* cf. *taurica* (Apidae) on *Phelypaea coccinea*, Georgia; F: *Ceratina cyanea* (Apidae) on *Phelipanche arenaria*, Poland; G: Pteromalidae from *O. hederae*, Georgia; H: *Lasioglossum* sp. on *O. artemisiae-campestris*, North Macedonia; I: *Rophites hartmanni* (Halictidae) on *P. arenaria*, Poland; J: *Andrena* sp. (Andrenidae) on *Cistanche tubulosa*, Qatar; K: *Dianthidium* sp. (Megachilidae) on *Aphyllon arizonicum*, USA; L: *Osmia aurulenta* (Megachilidae) on *O. lutea*, Poland; M: *Dolichovespula sylvestris* (Vespidae) on *O. flava*, Romania; N: *Polistes* sp. (Vespidae) and *Anthophora* sp. (Apidae) on *C. mauritanica*, Morocco; O: *P. dominula* (Vespidae) on *O. alsatica*, Poland; P: *Symmorphus gracilis* (Vespidae) on *O. alba*, Crimea. Phot. R. Piwowarczyk (E, G, I, L, O), A. Mátis (B, C, M), A. Fateryga (A, P), K. Wiśniewska (F), F. Bioret (D), Z. Nikolov (H), (J) photo by upandiyan from the iNaturalist web-site (https://www.inaturalist.org/photos/268521577, modified – cropped), (K) photo by Will Pearce from the iNaturalist web-site (https://www.inaturalist.org/photos/463255372), (N) photo by mimounbachiri from the iNaturalis web-site (https://www.inaturalist.org/photos/180577864), J, K, N: distributed under the terms of the Creative Commons CC BY-NC 4.0 license (https://creativecommons.org/licenses/by-nc/4.0/).

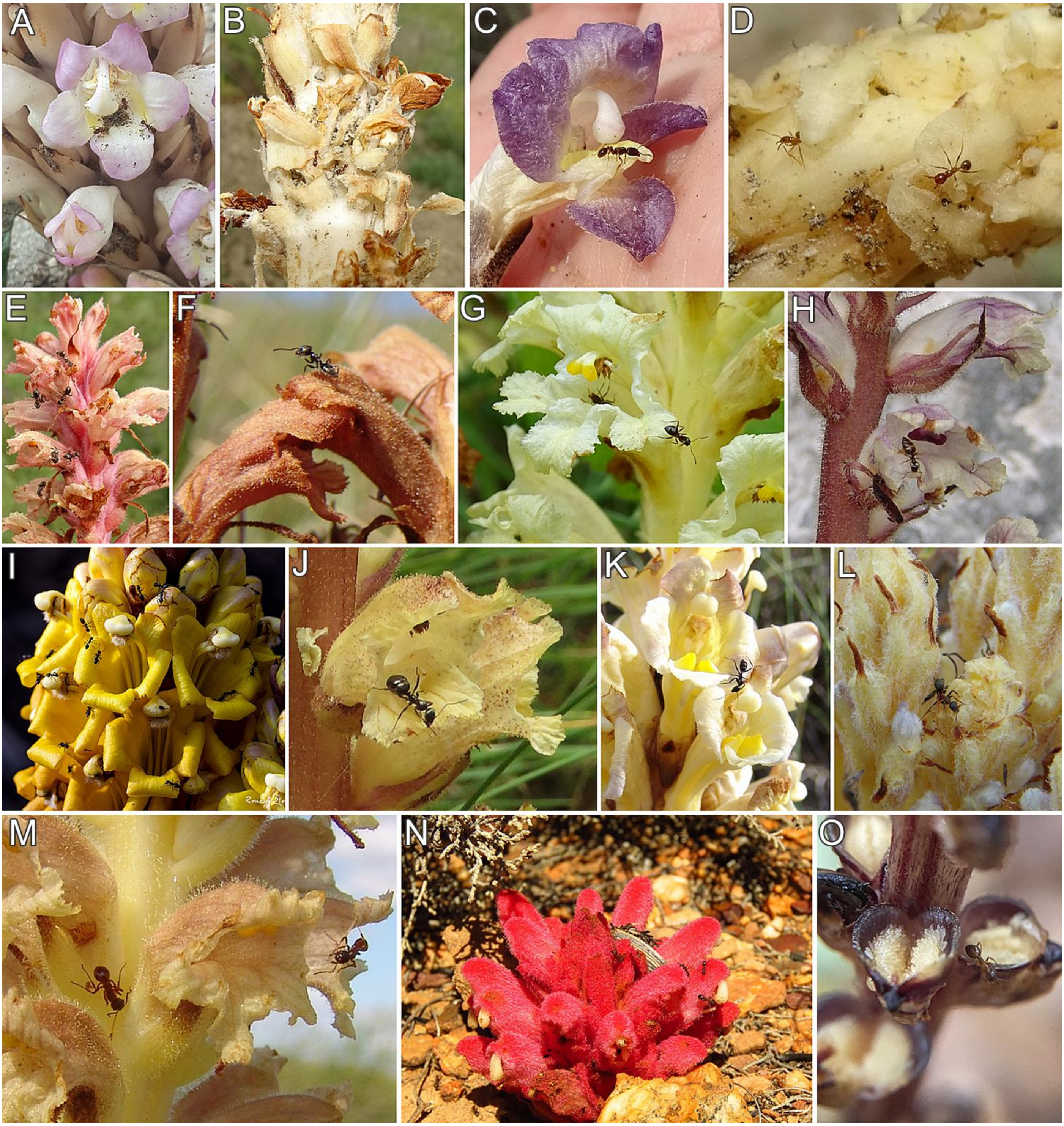

Figure S2.6. Representative species of Hymenoptera (Formicidae). A: *Tetramorium immigrans* on *Cistanche flava*, Azerbaijan; B, C: temporary colonies of *T. immigrans* in inflorescences of *C. fissa*, Azerbaijan; D: *Pheidole koshewnikovi* on underground stems of *C. fissa*, Azerbaijan; E: *Lasius flavus* on *Orobanche centaurina*, Poland; F: *Lasius* sp. on *O. teucrii*, Austria; G: *Lasius* sp. on *O. grossheimii*, Armenia; H: *Plagiolepis* sp. on *O. minor*, Montenegro; I: *Crematogaster* sp. on *C. tubulosa*, India; J: *Formica gagates* on *O. reticulata*, Romania; K: *Formica* sp. on *C. phelypaea*, Spain; L: *F. rufibarbis* complex on *O. coerulescens*, Poland; M: *Myrmica* sp. on *O. elatior*, Poland; N: *Anoplolepis* sp. on *Hyobanche sanguinea*, South Africa; O: *Nylanderia* on open fruits with seeds of *Epifagus virginiana*, USA. Phot. R. Piwowarczyk (A–H, K–M), A. Mátis (J), (I) photo by Ramesh Shenai Jr. from the iNaturalist web-site (https://www.inaturalist.org/photos/87768119, modified – cropped), (N) photo by Marienne de Villiers from the iNaturalist web-site (https://www.inaturalist.org/photos/28890854), (O) photo by Misha Zitser from the iNaturalist web-site (https://www.inaturalist.org/photos/435793625), I, N, O: distributed under the terms of the Creative Commons CC BY-NC 4.0 license (https://creativecommons.org/licenses/by-nc/4.0/).

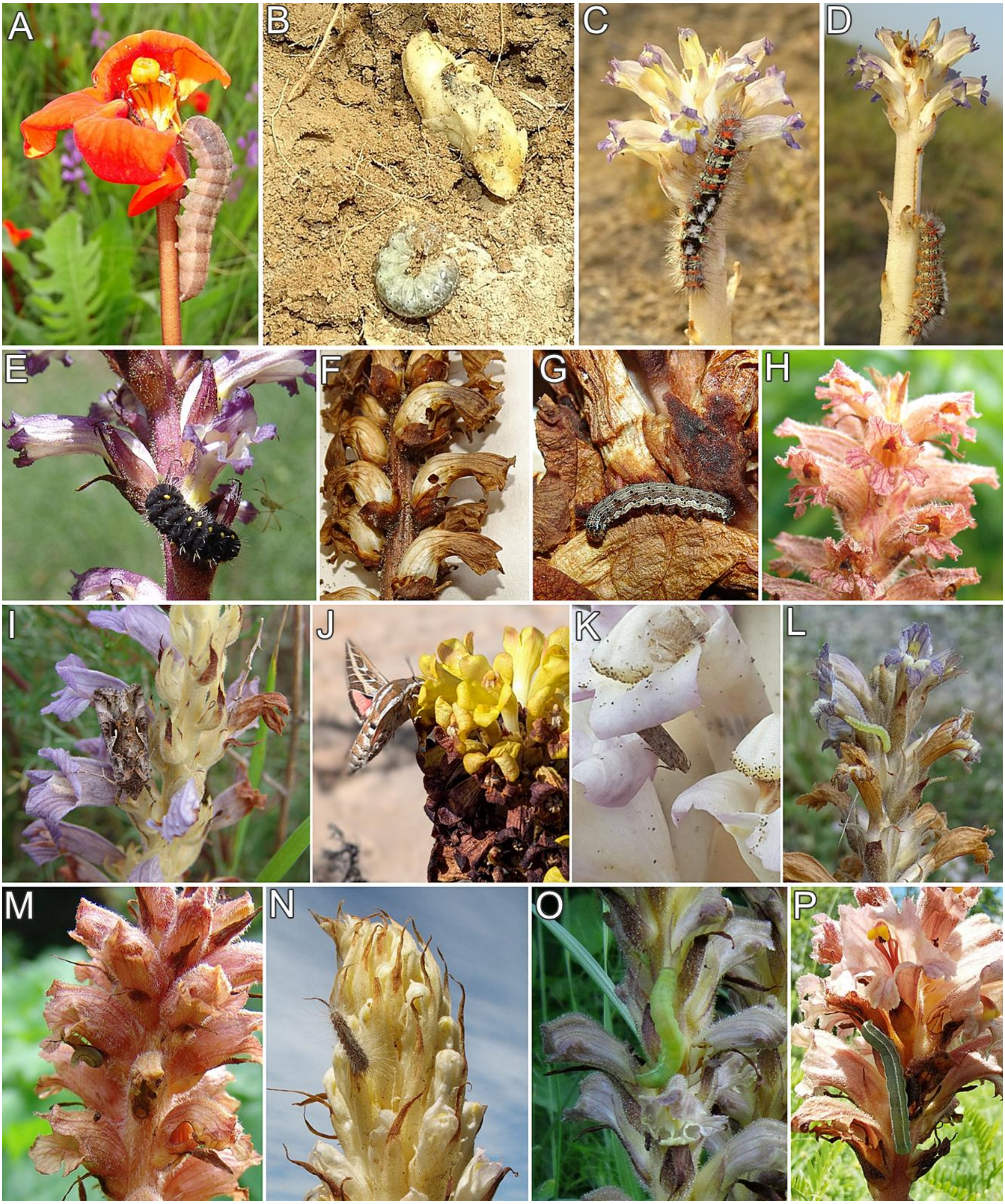

Figure S2.7. Representative species of Lepidoptera. A: *Ammoconia senex* (Noctuidae) on *Phelypaea coccinea*, Georgia; B: *Anarta sabulorum* (Noctuidae) on tuber of *Cistanche fissa*, Azerbaijan; C, D: *Orgyia dubia* (Erebidae) on *Orobanche cumana*, Azerbaijan; E: *Zygaena* sp. (Zygaenidae) on *O. grenieri*, Georgia; F: holes eaten by *Helicoverpa armigera* (Noctuidae) in the fruits of *O. cumana*, Azerbaijan; G: *Helicoverpa armigera* (Noctuidae) on *C. phelypaea*, Spain; H: Geomitridae on *O. flava*, Ukraine; I: *Autographa gamma* (Noctuidae) on *Phelipanche arenaria*, Poland; J: *Hyles livornica* (Sphingidae) pollinating *C. phelypaea*, Canary Islands; K: Noctuidae on *C. flava*, Azerbaijan; L: Lepidoptera on *P. cernua*, Armenia; M: *Lepidoptera* on *O. flava*, Slovakia; N: Pterophoridae on *O. kotschyi*, Kazakhstan; O: Noctuidae on *O. lutea*, Romania; P: *Antitype chi* (Noctuidae) on *O. lutea*, Austria. Phot. R. Piwowarczyk (A–I, K–M), C. Thorogood (J), A. Mátis (O); (P) photo by Andreas Berger from the iNaturalist web-site (https://www.inaturalist.org/photos/132318222), distributed under the terms of the Creative Commons CC BY-NC 4.0license (https://creativecommons.org/licenses/by-nc/4.0/); (M) photo by Vladimir Kolbintsev.

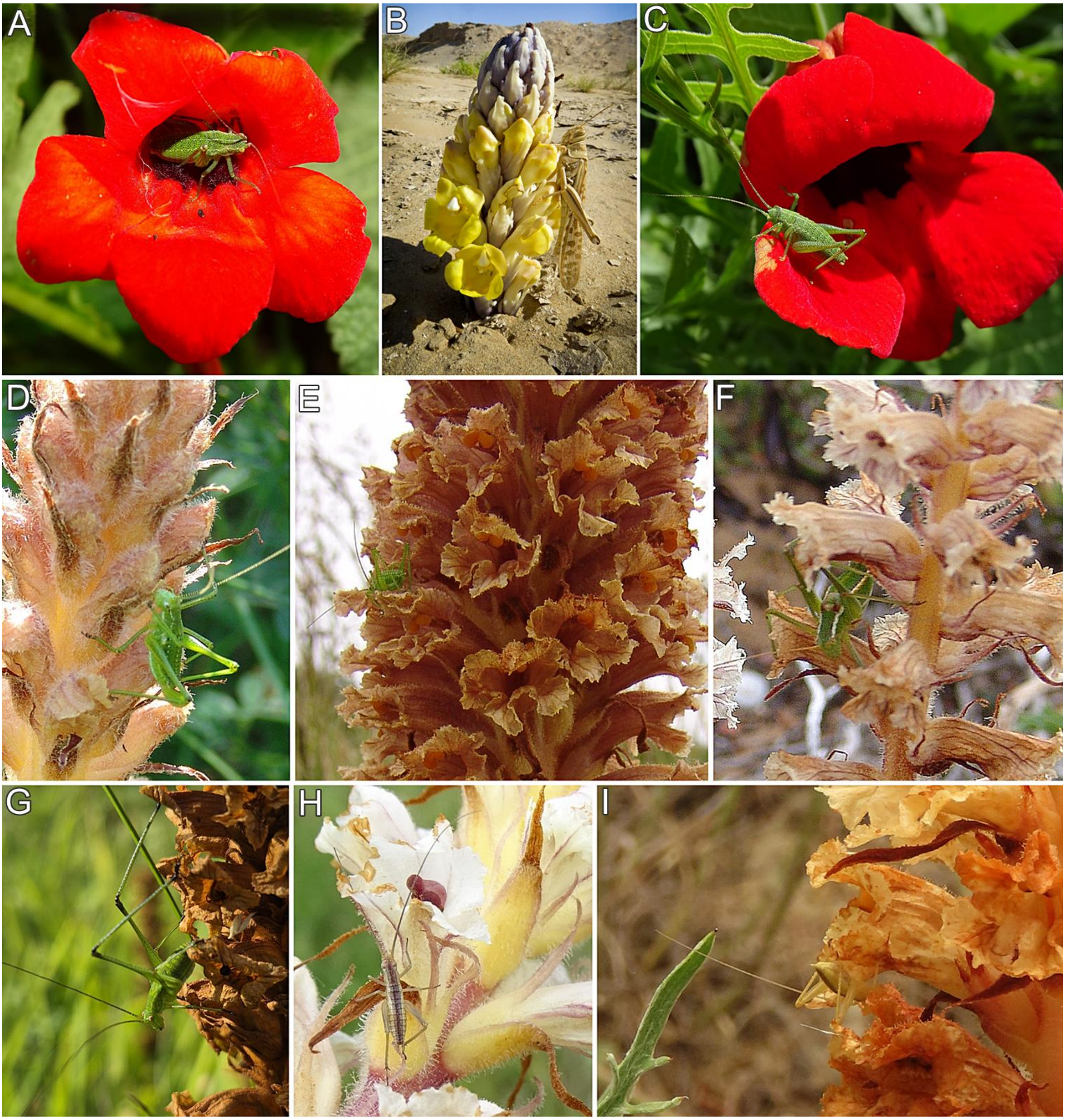

Figure S2.8. Representative species of Orthoptera. A, C: *Leptophyes* sp. (Tettigoniidae) on *Phelypaea coccinea*, Georgia; B: *Schistocerca gregaria* (Acrididae) on *Cistanche lutea*, Oman; D: *L. albovittata* (Tettigoniidae) on *Orobanche bartlingii*, Poland; E: *L. albovittata* (Tettigoniidae) on *O. elatior*, Poland; F: *Poecilimon* sp. (Tettigoniidae) on *O. minor*, Portugal; G: *Phaneroptera falcata* (Tettigoniidae) on *O. alsatica*, Poland; H: juvenile *Oecanthus pellucens* (Gryllidae) on *O. picridis*, Poland; I: *O. pellucens* (Gryllidae) on *O. centaurina*, Romania. Phot. R. Piwowarczyk (A–H), (B) photo by S. Pilloni from the iNaturalist web-site (https://www.inaturalist.org/photos/465315593), distributed under the terms of the Creative Commons CC BY-NC 4.0 license (https://creativecommons.org/licenses/by-nc/4.0/), A. Mátis (I).

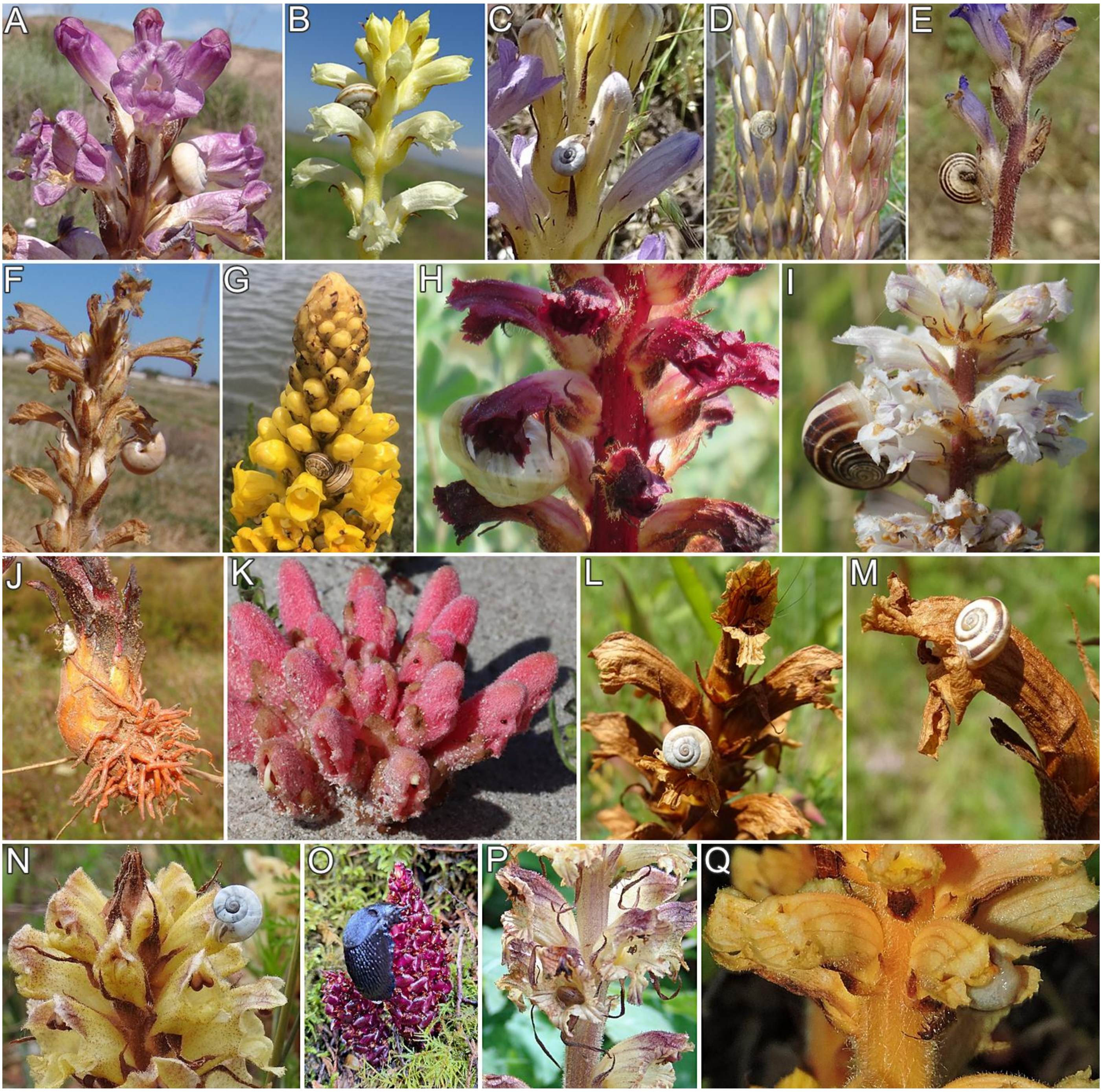

Figure S2.9. Representative species of Stylommatophora. *Xeropicta derbentina* (Geomitridae) on A: *Cistanche armena*, Armenia; B: *Orobanche cumana*, Ukraine; C: *Phelipanche arenaria*, Georgia; D: *C. flava*, Azerbaijan; E: *Phelipanche* sp., Armenia; *Theba pisana* (Helicidae) on F: *P. nana*, Crete; G: *C. phelypaea*, Portugal; H: *O. sanguinea*, Italy; I: *Cepaea hortensis* (Helicidae) on *O. crenata*, Italy; J: *Cochlicella acuta* (Geomitridae) on *O. foetida*, Portugal; K: *Zonitoides arboreus* (Gastrodontidae) on *Hyobanche sanguinea*, South Africa; L, M: *Xerolenta obvia* (Geomitridae) on *O. lutea*, Poland; N: *Chilostoma cingulatum* (Helicidae) on *O. reticulata*, France; O: *Arion* sp. (Arionidae) on *Kopsiopsis hookeri*, Canada; P: slug in flower of *O. reticulata*, Poland; Q: slug in flower of *O. flava*, Romania. Phot. R. Piwowarczyk (A–F, J, L, M, P), A. Mátis (Q), (G) photo by mjdrumond from the iNaturalist web-site (https://www.inaturalist.org/photos/131512991, modified – cropped), (H) photo by Stephen Moores from the iNaturalist web-site (https://www.inaturalist.org/photos/28023990, modified – cropped), (I) photo by mick_photo from the iNaturalist web-site (https://www.inaturalist.org/photos/223719581, modified – cropped), (N) photo by jltasset from the iNaturalist web-site (https://www.inaturalist.org/photos/311925333, modified – cropped), (K) photo by Tony Rebelo from the iNaturalist web-site (https://www.inaturalist.org/photos/319711535, modified – cropped), (O) photo by odf from the iNaturalist web-site (https://www.inaturalist.org/photos/384481976, modified – cropped), (G, H, I, N, O) distributed under the terms of the Creative Commons CC BY-NC 4.0 license (https://creativecommons.org/licenses/by-nc/4.0/), (K) distributed under the terms of the Creative Commons CC BY-SA license (https://creativecommons.org/licenses/by-sa/4.0/).

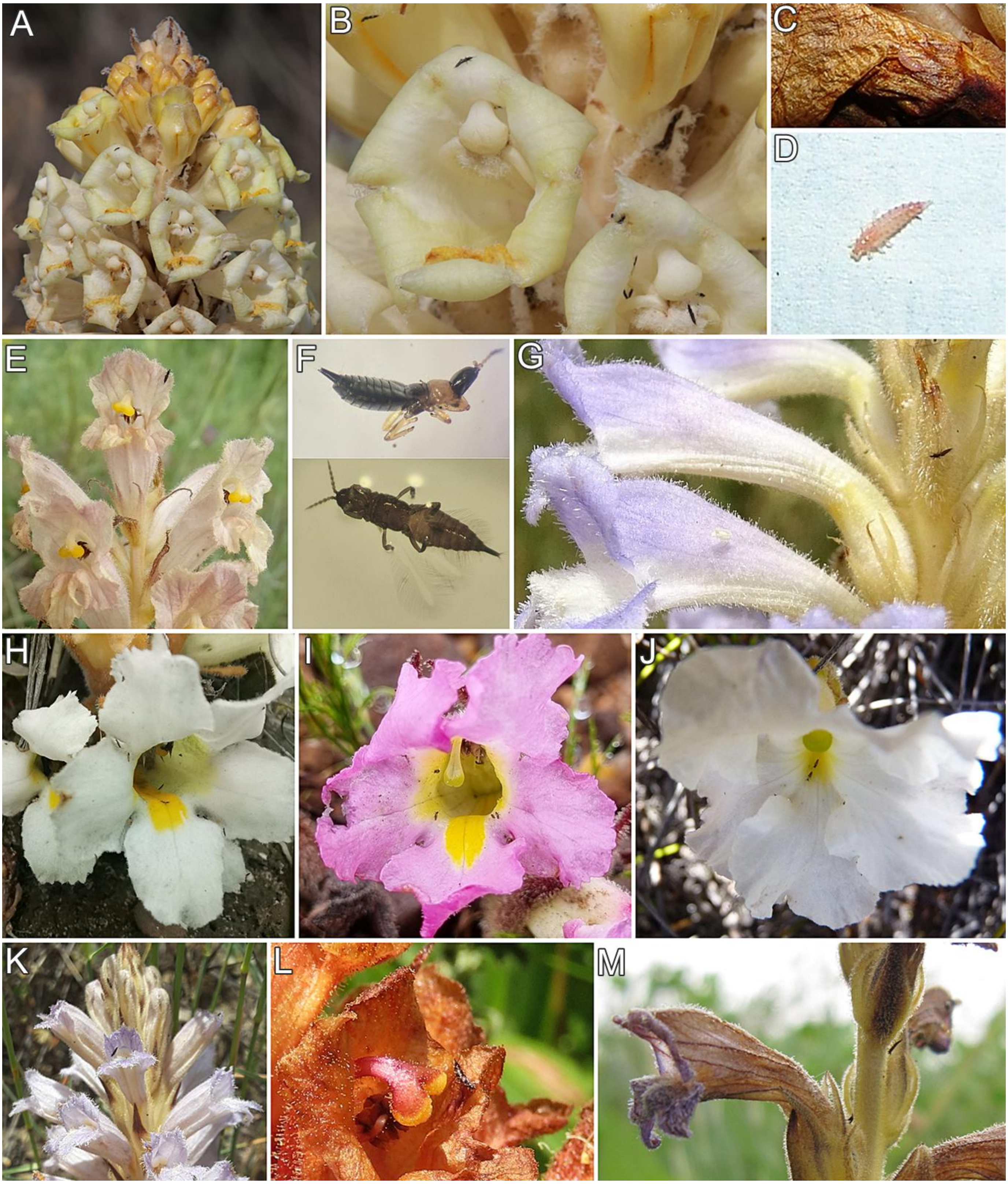

Figure S2.10. Representative species of Thysanoptera. A, B: Tubulifera group (Phlaeothripidae) on *Cistanche* cf. *trivalvis*, Uzbekistan; C, D: Phlaeothripidae larva on *C. phelypaea*, Spain; E: cf. Tubulifera (Phlaeothripidae) on *Orobanche caryophyllacea*, Georgia; F: *Haplothrips* sp. (Phlaeothripidae) from *O. laxissima*, Georgia; G: cf. Tubulifera (Phlaeothripidae) on *Phelipanche nana*, Georgia; H, J: cf. Terebrantia (Phlaeothripidae) on *Harveya capensis*, South Africa; I: cf. Terebrantia (Phlaeothripidae) on *H. purpurea*, South Africa; K: cf. Terebrantia (Phlaeothripidae) on *Phelipanche* sp., Armenia; L: Tubulifera group (Phlaeothripidae) on *O. alba*, Turkey; M: cf. Tubulifera (Phlaeothripidae) on *P. purpurea*, Romania. Phot. R. Piwowarczyk (C–G, K), A. Fateryga (A, B), A. Mátis (L, M), (H) photo by brendanj from the iNaturalist web-site (https://www.inaturalist.org/photos/191587686, modified – cropped), (I) photo by Santie Gouws from the iNaturalist web-site (https://www.inaturalist.org/photos/174404834, modified – cropped), (J) photo by Marge from the iNaturalist web-site (https://www.inaturalist.org/photos/15423003, modified – cropped), (H–J) distributed under the terms of the Creative Commons CC BY-NC 4.0 license (https://creativecommons.org/licenses/by-nc/4.0/).

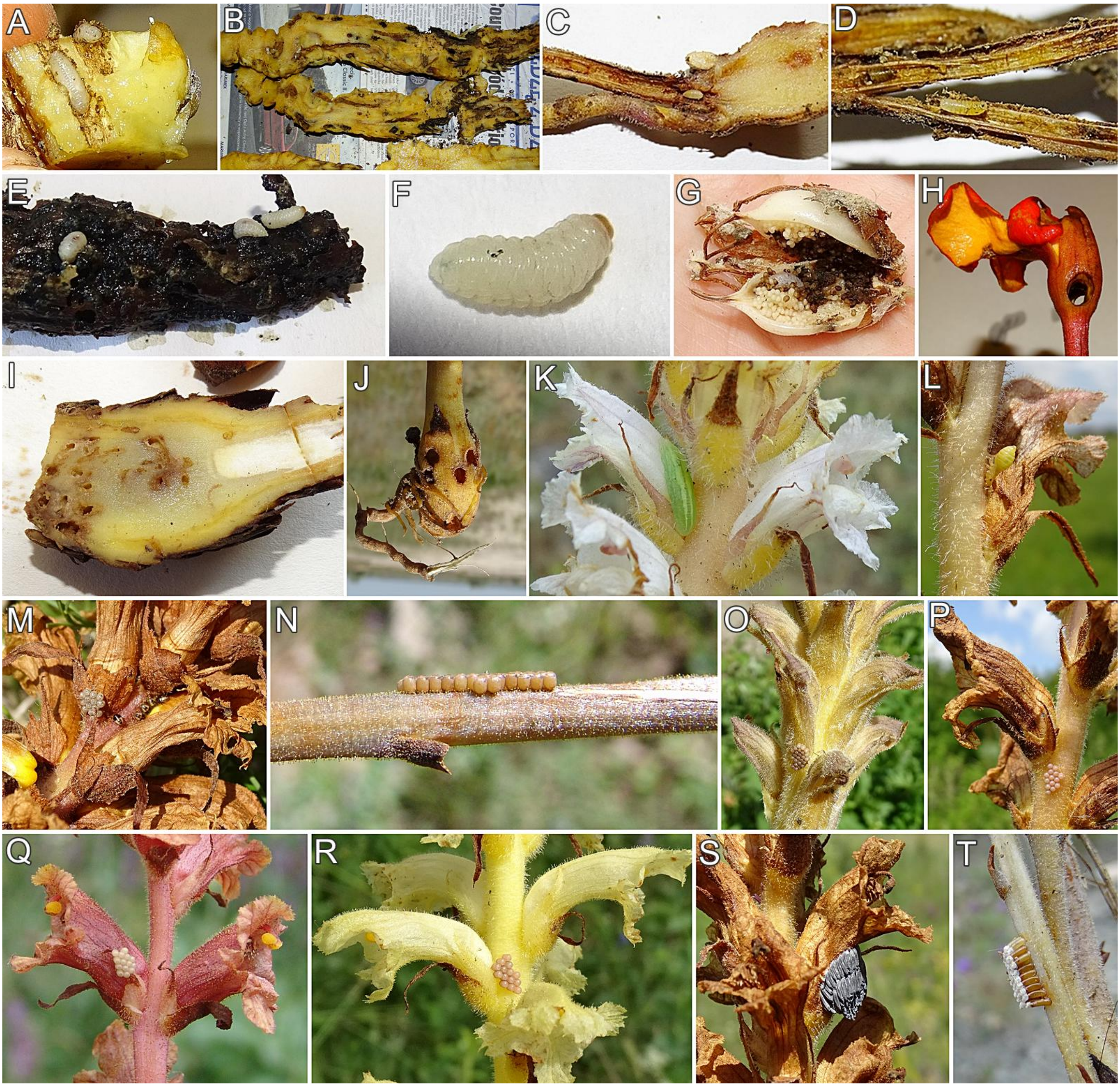

Figure S2.11. Examples of laid eggs, juvenile stages and mining traces of insects on Orobanchaceae tissues. A: larvae of *Eumerus mucidus* (Syrphidae) in stem of *Cistanche fissa*, Azerbaijan; B: traces of mining of *E. mucidus* (Syrphidae) in stem of *C. armena*, Armenia; C, D: traces of mining and larvae of *Phytomyza orobanchia* (Agromyzidae) in stem and tuber of *Phelipanche ramosa*, Poland; E, F: larvae of Curculionidae in stem of *P. caesia*, Azerbaijan; G: larvae of *Smicronyx fulvipes* (Curculionidae) in fruit with seeds of *C. fissa*, Azerbaijan; H: traces of feeding of a Lepidoptera caterpillar in a young fruit of *Phelypaea coccinea*, Georgia; I: traces of mining and larvae of Diptera in tuber of *O. laxissima*, Georgia; J: traces of mining in tuber of *O. cumana*, Ukraine; K: larvae of *Sphaerophoria* cf. *scripta* (Syrphidae) on *O. minor*, Georgia; L: *Philaenus spumarius* (Aphrophoridae) on *O. caryophyllacea*, Poland; M: eggs and juvenile of *Dolycoris baccarum* (Pentatomidae) on *O. lutea*, Poland; Hemiptera eggs on N: *Phelipanche* sp., Armenia; O: *O. cicerbitae*, Georgia; P: *O. lutea*, Poland; Q: *O. alba*, Georgia; R: *O. cicerbitae*, Georgia; S: eggs of *Tabanus* sp. (Tabanidae) on *O. owerinii*, Armenia; T: eggs of cf. Lepidoptera on *P. cernua*, Armenia. Phot. R. Piwowarczyk.
