# Supplementary material for: A multiverse of trophic networks and coevolutionary trajectories among holoparasitic Orobanchaceae and their animal associates: a global perspective
Source: PhytoKeys. 2026 Jun 2;275:209–97. doi: 10.3897/phytokeys.275.192014 (PMC13250618; doi:10.3897/phytokeys.275.192014)
Supplement: Supplementary material 5 — Additional tables [file phytokeys-275-209_article-192014__-s005.pdf]

Table S3. Biodiversity metrics of identified fauna (orders) inhabiting parasitic plant genera.

	Taxa_S	Observations	Dominance_D	Simpson_1-D	Shannon_H	Evenness_e^H/S	Brillouin	Menhinick	Margalef	Equitability_J	Fisher_alpha	Berger-Parker	Chao-1
Aeginetia	7	15	0.253	0.747	1.622	0.723	1.209	1.807	2.216	0.833	5.109	0.400	17
Aphyllon	5	24	0.319	0.681	1.316	0.746	1.095	1.021	1.259	0.818	1.922	0.458	5
Boschniakia	4	13	0.302	0.698	1.266	0.887	0.984	1.109	1.170	0.913	1.974	0.385	4
Christisonia	3	4	0.375	0.625	1.040	0.943	0.621	1.500	1.443	0.946	5.453	0.500	3.5
Cistanche	20	190	0.133	0.867	2.300	0.499	2.147	1.451	3.621	0.768	5.639	0.221	38
Conopholis	8	29	0.237	0.763	1.718	0.696	1.415	1.486	2.079	0.826	3.652	0.414	9.5
Epifagus	4	15	0.378	0.622	1.137	0.779	0.895	1.033	1.108	0.820	1.785	0.533	4
Harveya	6	16	0.250	0.750	1.548	0.784	1.195	1.500	1.803	0.864	3.487	0.375	9
Hyobanche	9	36	0.397	0.603	1.434	0.466	1.174	1.500	2.232	0.653	3.852	0.611	11
Kopsiopsis	4	9	0.383	0.617	1.149	0.789	0.814	1.333	1.365	0.829	2.759	0.556	4.5
Lathraea	9	59	0.344	0.656	1.459	0.478	1.279	1.172	1.962	0.664	2.959	0.542	12
Orobanche	23	761	0.193	0.807	2.018	0.327	1.967	0.834	3.316	0.644	4.472	0.364	26
Phacellanthus	5	18	0.346	0.654	1.268	0.711	1.006	1.179	1.384	0.788	2.293	0.500	5.5
Phelipanche	12	125	0.165	0.836	2.019	0.628	1.873	1.073	2.278	0.813	3.270	0.272	13.5
Phelypaea	7	27	0.317	0.683	1.434	0.600	1.174	1.347	1.820	0.737	3.066	0.482	13
Xylanche	1	1	1	0	0	1	0	1	0		0	1	1

Table S4. Taxonomic composition of fauna associated with parasitic plant genera, expressed as the proportion (%) of observations for each taxonomic group.

	Aeginetia	*Aphyllon*	*Boschniakia*	*Christisonia*	*Cistanche*	*Conopholis*	*Epifagus*	*Harveya*	*Hyobanche*	*Kopsiopsis*	*Lathraea*	*Orobanche*	*Phacellanthus*	*Phelipanche*	*Phelypaea*	*Xylanche*
**Hymenoptera**	40.0	29.2	23.1	50.0	22.1	41.4	53.3	6.3	8.3	55.6	54.2	36.4	27.8	27.2	11.1	0.0
**Stylommatophora**	0.0	0.0	30.8	0.0	5.3	3.4	0.0	18.8	61.1	22.2	16.9	3.4	0.0	6.4	0.0	100.0
**Coleoptera**	6.7	12.5	7.7	0.0	11.1	3.4	13.3	25.0	0.0	0.0	11.9	10.8	50.0	13.6	48.1	0.0
**Hemiptera**	0.0	45.8	0.0	25.0	3.2	13.8	6.7	0.0	0.0	0.0	3.4	10.1	0.0	20.8	3.7	0.0
**Diptera**	6.7	8.3	0.0	25.0	19.5	6.9	0.0	0.0	0.0	0.0	6.8	13.9	0.0	12.0	0.0	0.0
**Araneae**	0.0	0.0	0.0	0.0	6.8	0.0	26.7	6.3	0.0	0.0	1.7	12.4	0.0	7.2	25.9	0.0
**Thysanoptera**	6.7	4.2	0.0	0.0	6.8	0.0	0.0	37.5	0.0	0.0	0.0	3.4	0.0	6.4	3.7	0.0
**Lepidoptera**	26.7	0.0	0.0	0.0	13.7	0.0	0.0	6.3	0.0	0.0	1.7	3.8	0.0	1.6	3.7	0.0
**Carnivora**	0.0	0.0	38.5	0.0	0.5	13.8	0.0	0.0	0.0	0.0	0.0	0.0	0.0	0.0	0.0	0.0
**Rodentia**	0.0	0.0	0.0	0.0	0.0	13.8	0.0	0.0	2.8	11.1	0.0	0.1	0.0	0.0	0.0	0.0

**Orthoptera**	0.0	0.0	0.0	0.0	1.1	0.0	0.0	0.0	0.0	0.0	0.0	2.4	11.1	0.8	3.7	0.0
**Nematoda**	0.0	0.0	0.0	0.0	0.0	0.0	0.0	0.0	0.0	11.1	0.0	0.0	0.0	0.8	0.0	0.0
**Artiodactyla**	0.0	0.0	0.0	0.0	0.5	3.4	0.0	0.0	2.8	0.0	0.0	0.0	0.0	2.4	0.0	0.0
**Passeriformes**	0.0	0.0	0.0	0.0	0.0	0.0	0.0	0.0	8.3	0.0	0.0	0.3	0.0	0.0	0.0	0.0
**Isopoda**	0.0	0.0	0.0	0.0	2.6	0.0	0.0	0.0	0.0	0.0	0.0	0.1	5.6	0.0	0.0	0.0
**Blattodea**	6.7	0.0	0.0	0.0	0.0	0.0	0.0	0.0	0.0	0.0	0.0	0.3	0.0	0.0	0.0	0.0
**Odonata**	6.7	0.0	0.0	0.0	0.0	0.0	0.0	0.0	0.0	0.0	0.0	0.0	0.0	0.0	0.0	0.0
**Testudines**	0.0	0.0	0.0	0.0	0.5	0.0	0.0	0.0	5.6	0.0	0.0	0.0	0.0	0.0	0.0	0.0
**Dermaptera**	0.0	0.0	0.0	0.0	0.0	0.0	0.0	0.0	0.0	0.0	0.0	0.1	5.6	0.0	0.0	0.0
**Macroscelidea**	0.0	0.0	0.0	0.0	0.0	0.0	0.0	0.0	5.6	0.0	0.0	0.0	0.0	0.0	0.0	0.0
**Sarcoptiformes**	0.0	0.0	0.0	0.0	3.2	0.0	0.0	0.0	0.0	0.0	0.0	0.4	0.0	0.0	0.0	0.0
**Squamata**	0.0	0.0	0.0	0.0	0.5	0.0	0.0	0.0	2.8	0.0	0.0	0.0	0.0	0.0	0.0	0.0
**Primates**	0.0	0.0	0.0	0.0	0.0	0.0	0.0	0.0	2.8	0.0	0.0	0.0	0.0	0.0	0.0	0.0
**Mesostigmata**	0.0	0.0	0.0	0.0	0.5	0.0	0.0	0.0	0.0	0.0	1.7	0.4	0.0	0.0	0.0	0.0
**Collembola**	0.0	0.0	0.0	0.0	0.0	0.0	0.0	0.0	0.0	0.0	1.7	0.4	0.0	0.0	0.0	0.0
**Neuroptera**	0.0	0.0	0.0	0.0	0.5	0.0	0.0	0.0	0.0	0.0	0.0	0.0	0.0	0.8	0.0	0.0
**Psocodea**	0.0	0.0	0.0	0.0	0.5	0.0	0.0	0.0	0.0	0.0	0.0	0.3	0.0	0.0	0.0	0.0
**Anura**	0.0	0.0	0.0	0.0	0.5	0.0	0.0	0.0	0.0	0.0	0.0	0.0	0.0	0.0	0.0	0.0
**Trombidiformes**	0.0	0.0	0.0	0.0	0.5	0.0	0.0	0.0	0.0	0.0	0.0	0.0	0.0	0.0	0.0	0.0
**Polyxenida**	0.0	0.0	0.0	0.0	0.0	0.0	0.0	0.0	0.0	0.0	0.0	0.4	0.0	0.0	0.0	0.0
**Crassiclitellata**	0.0	0.0	0.0	0.0	0.0	0.0	0.0	0.0	0.0	0.0	0.0	0.3	0.0	0.0	0.0	0.0
**Opiliones**	0.0	0.0	0.0	0.0	0.0	0.0	0.0	0.0	0.0	0.0	0.0	0.1	0.0	0.0	0.0	0.0
**Plecoptera**	0.0	0.0	0.0	0.0	0.0	0.0	0.0	0.0	0.0	0.0	0.0	0.1	0.0	0.0	0.0	0.0
**Spirobolida**	0.0	0.0	0.0	0.0	0.0	0.0	0.0	0.0	0.0	0.0	0.0	0.1	0.0	0.0	0.0	0.0
**Propotion of observations (%)**			0.1-20			20.1-40			40.1-60			60.1-80			>80.1	

Table S5. Proportional distribution (%) of observations of fauna associated with parasitic plant genera, showing (A) visited plant parts, (B) developmental stages of fauna, and (C) ecological roles of fauna.

**A**	*Aeginetia*	*Aphyllon*	*Boschniakia*	*Christisonia*	*Cistanche*	*Conopholis*	*Epifagus*	*Harveya*	*Hyobanche*	*Kopsiopsis*	*Lathraea*	*Orobanche*	*Phacellanthus*	*Phelipanche*	*Phelypaea*	*Xylanche*
**flowers**	85.7	87.0	66.7	100.0	62.8	92.6	93.3	100.0	80.0	100.0	91.1	88.9	16.7	78.8	100.0	100.0
**flowers. stem**	0.0	8.7	0.0	0.0	3.9	0.0	0.0	0.0	0.0	0.0	3.6	1.9	61.1	5.9	0.0	0.0
**flowers. stem. fruits/seeds**	0.0	0.0	0.0	0.0	0.6	3.7	0.0	0.0	0.0	0.0	0.0	1.9	0.0	0.8	0.0	0.0
**fruits/seeds**	7.1	0.0	0.0	0.0	5.0	3.7	6.7	0.0	0.0	0.0	5.4	2.2	16.7	0.0	0.0	0.0

**stem**	7.1	4.3	0.0	0.0	3.9	0.0	0.0	0.0	0.0	0.0	0.0	3.0	0.0	3.4	0.0	0.0
**underground stem and tuber**	0.0	0.0	25.0	0.0	23.9	0.0	0.0	0.0	0.0	0.0	0.0	1.9	0.0	8.5	0.0	0.0
**whole plant or parts**	0.0	0.0	8.3	0.0	0.0	0.0	0.0	0.0	20.0	0.0	0.0	0.1	5.6	2.5	0.0	0.0
**Proportion of observations (%)**			0.1-20			20.1-40			40.1-60			60.1-80			>80.1	

**B**	*Aeginetia*	*Aphyllon*	*Boschniakia*	*Christisonia*	*Cistanche*	*Conopholis*	*Epifagus*	*Harveya*	*Hyobanche*	*Kopsiopsis*	*Lathraea*	*Orobanche*	*Phacellanthus*	*Phelipanche*	*Phelypaea*	*Xylanche*
**adult**	73.3	91.7	100.0	100.0	68.1	89.7	92.9	93.8	97.2	100.0	96.4	77.3	100.0	81.8	92.6	100.0
**adult. larva**	0.0	0.0	0.0	0.0	12.4	0.0	7.1	0.0	0.0	0.0	0.0	3.1	0.0	6.6	0.0	0.0
**adult. larva. eggs**	0.0	0.0	0.0	0.0	0.0	0.0	0.0	0.0	0.0	0.0	0.0	0.3	0.0	0.8	0.0	0.0
**eggs**	0.0	0.0	0.0	0.0	0.0	0.0	0.0	0.0	0.0	0.0	0.0	2.3	0.0	1.7	0.0	0.0
**exuvium**	0.0	4.2	0.0	0.0	0.0	0.0	0.0	0.0	0.0	0.0	0.0	0.4	0.0	0.0	0.0	0.0
**juvenile**	0.0	0.0	0.0	0.0	4.9	0.0	0.0	6.3	2.8	0.0	0.0	10.0	0.0	3.3	0.0	0.0
**larva**	26.7	4.2	0.0	0.0	14.6	10.3	0.0	0.0	0.0	0.0	3.6	6.6	0.0	5.8	7.4	0.0
**Proportion of observations (%)**			0.1-20			20.1-40			40.1-60			60.1-80			>80.1	

**C**	*Aeginetia*	*Aphyllon*	*Boschniakia*	*Christisonia*	*Cistanche*	*Conopholis*	*Epifagus*	*Harveya*	*Hyobanche*	*Kopsiopsis*	*Lathraea*	*Orobanche*	*Phacellanthus*	*Phelipanche*	*Phelypaea*	*Xylanche*
**anthophylous**	26.7	33.3	23.1	0.0	22.9	30.8	33.3	15.4	26.3	55.6	60.8	35.2	0.0	34.2	55.6	0.0
**carnivorous**	6.7	0.0	0.0	0.0	9.0	0.0	26.7	7.7	5.3	0.0	3.9	14.8	0.0	8.3	25.9	0.0
**detritivorous**	0.0	0.0	0.0	0.0	3.7	0.0	0.0	0.0	0.0	0.0	0.0	2.5	10.0	0.0	0.0	0.0
**fungivorous**	0.0	0.0	0.0	0.0	0.0	0.0	0.0	0.0	0.0	0.0	0.0	0.3	0.0	0.0	0.0	0.0
**hemizoophagous**	0.0	0.0	0.0	0.0	0.0	0.0	0.0	0.0	0.0	0.0	3.9	1.4	0.0	1.7	0.0	0.0
**omnivorous**	20.0	9.5	0.0	100.0	16.5	15.4	20.0	7.7	15.8	0.0	9.8	8.8	50.0	6.7	0.0	0.0
**parasitoids**	0.0	0.0	0.0	0.0	0.0	0.0	0.0	0.0	0.0	0.0	0.0	3.4	0.0	0.0	0.0	0.0
**phytophagous**	40.0	57.1	76.9	0.0	37.8	46.2	20.0	53.8	47.4	44.4	13.7	27.3	10.0	43.3	18.5	100.0
**phytophagous. granivorous**	6.7	0.0	0.0	0.0	4.8	7.7	0.0	0.0	0.0	0.0	5.9	3.8	30.0	0.8	0.0	0.0
**resting**	0.0	0.0	0.0	0.0	5.3	0.0	0.0	15.4	5.3	0.0	2.0	2.5	0.0	5.0	0.0	0.0
**Proportion of observations (%)**			0.1-20			20.1-40			40.1-60			60.1-80			>80.1	
